# Design, synthesis, docking, and anticancer evaluations of new thiazolo[3,2-*a*] pyrimidines as topoisomerase II inhibitors

**DOI:** 10.1080/14756366.2023.2175209

**Published:** 2023-02-12

**Authors:** Mona S. El-Zoghbi, Samiha A. El-Sebaey, Hanan A. AL-Ghulikah, Eman A. Sobh

**Affiliations:** a Department of Pharmaceutical Chemistry, Menoufia University, Menoufia, Egypt; b Department of Pharmaceutical Organic Chemistry, Al-Azhar University, Nasr City, Cairo, Egypt; c Department of Chemistry, College of Science, Princess Nourah bint Abdulrahman University, Riyadh, Saudi Arabia

**Keywords:** Topoisomerase II inhibitiors, thiazolopyrimidine, anticancer, cell cycle, apoptosis

## Abstract

New thiazolopyrimidine derivatives **2, 3a-d, 4a-c, 5, 6a-c,** and **7a,b** were synthesised. All prepared compounds were evaluated by MTT cytotoxicity assay against three human tumour cell lines. Compounds **3c, 3d, 4c, 6a, 6b,** and **7b** exhibited potent to strong anticancer activity that was nearly comparable or superior to Doxorubicin. Compounds exhibiting significant cytotoxicity were further selected to study their inhibitory activity on the Topo II enzyme. Compound **4c** was the most potent Topo II inhibitor with an IC_50_ value of 0.23 ± 0.01 µM, which was 1.4-fold and 3.6-fold higher than the IC_50_ values of Etoposide and Doxorubicin. Furthermore, compound **4c** showed significant cell cycle disruption and apoptosis on A549 cells compared to control cells. Molecular docking of the most active compounds illustrated proper fitting to the Topo II active site, suggesting that our designed compounds are promising candidates for the development of effective anticancer agents acting through Topo II inhibition.

## Graphical Abstract

**Figure:**
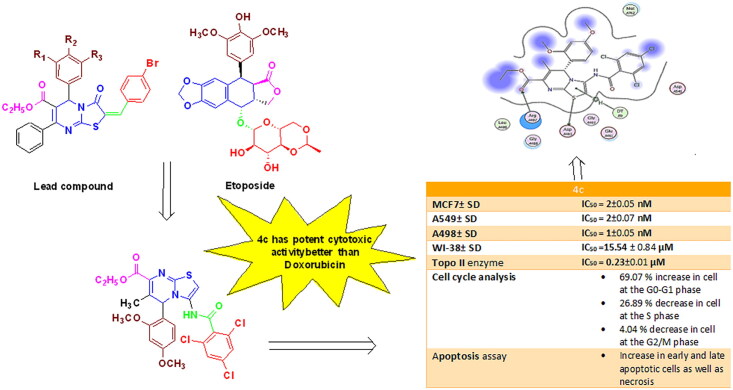

## 1. Introduction

**Figure 1.**
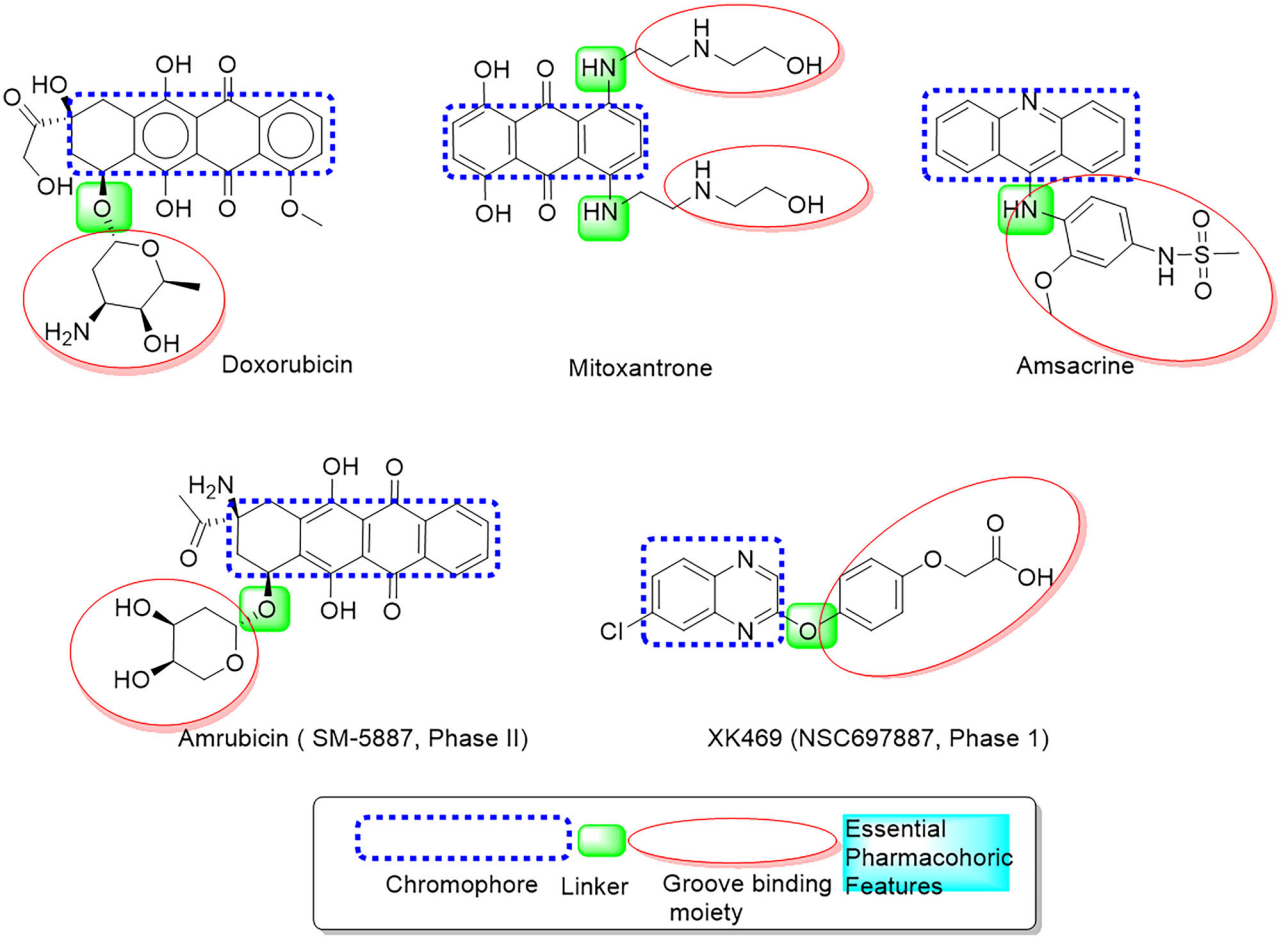
Basic features of some reported Topo II inhibitors.

Cancer is a serious health problem expected to affect approximately 22 million people worldwide by 2030. It is now the second main cause of morbidity and mortality after heart disease^1^, implying an urgent need for new and effective anticancer drugs. DNA intercalators are a type of DNA-damaging agent consisting of flat aromatic molecules that link two base pairs in a DNA molecule and cause DNA structural modifications such as DNA chain extension and partial unwinding. Numerous DNA intercalators utilised clinically in the therapy of malignancies act by inhibiting DNA and RNA synthesis or DNA damage *via* the inhibiting Topo I or Topo II^2–5^. Human DNA topoisomerase II (Topo II) is a significant target in the therapy of a wide range of tumours due to its critical role in initiating, controlling, and modifying topological DNA issues throughout cell growth, differentiation, and survival^6^. DNA topoisomerase II inhibitors are widely used in clinics for treating various solid tumours and haematological disorders^7^. Many anticancer drugs both inhibit Topo II and intercalate DNA, making them known as intercalative Topo II inhibitors. Extensive research confirmed that Intercalating Topo II inhibitors are well-established in inducing apoptosis^8^. Several Topo II inhibitors have already been used as anticancer drugs, including Doxorubicin, Mitoxantrone, and Amsacrine^9^, as well as XK469, a quinoxaline derivative (NSC697887, phase I), and Amrubicin, a 9-aminoanthracycline derivative (SM-5887, phase II), which were identified as selective Topo II inhibitors in clinical trials^10^. They all share an essential pharmacophore, which includes a planar aromatic system linked to a groove-binding side chain via a different linker, as illustrated in Figure 1. A thiazolopyrimidine is an isostere of purine that can be found in many drug skeletons. A thiazolo[3,2-*a*]pyrimidine scaffolds have drawn a lot of attention in the literature as DNA cleavage agents and antineoplastics^11–14^. It was found that their activity was due to their ability to intercalate with DNA, the primary target in cancer cells fighting^15^^,^^16^. The most effective clinical treatments involve the use of small molecules that primarily interact with nucleic acids through one of the following modes: Incorporating groove binding, intercalation, or electrostatic interactions^17^^,^^18^.

**Figure 2.**
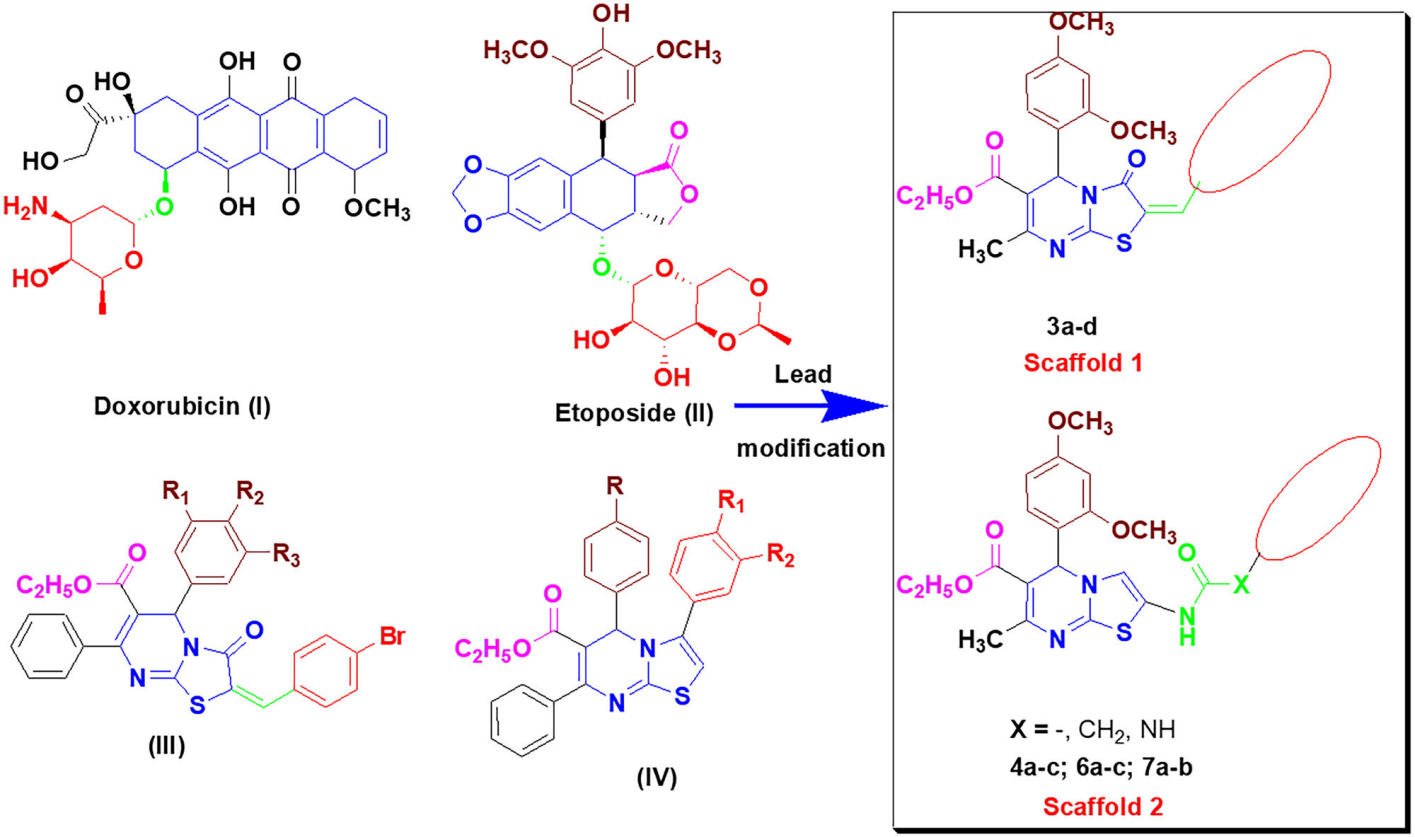
Rationale for designing new Topo II inhibitors.

Doxorubicin (**I**) and Etoposide (**II**) are two of the most important topoisomerase II inhibitors, serving as lead compounds for further research and development^19^. Structural features of both Doxorubicin and Etoposide had a planar fused aromatic system connected to a non-coplanar sugar or amino-sugar side chain. This side chain occupied the DNA minor groove, resulting in more stability and stronger binding. In addition, Etoposide possesses an extra trioxygenated phenyl moiety appended opposite the sugar ring. Some features have been proven to be important in several anticancer agents. Among these features, methoxy groups, ethyl carboxylate moiety, and amide linkers were discovered to boost antitumor activity^20–23^. On the other hand, a series of 3-oxo thiazolo[3,2-*a*]pyrimidines **(III)** were found to be significant topoisomerase II inhibitors^24^. Furthermore, thiazolo[3,2-*a*]pyrimidines **(IV)** have been reported to have excellent topoisomerase II inhibitory activity^25^ (Figure 2). In light of the above data, we developed two new series of 3-oxo-3,5-dihydrothiazolo[3,2-*a*]pyrimidine and thiazolo[3,2-*a*]pyrimidine analogues in a planar system with a 2,4-dimethoxy phenyl moiety as the first groove-binding side chain. The chemical modifications of the synthesised compounds were carried out on the pyrimidine-2-thione nucleus, compound **1**, by two pathways. The first was accomplished by the preparation of thiazalopyrimidin-3-one **3a**, followed by the insertion of polyoxygenated benzylidene moieties at C_2_ of the thiazolopyrimidine-3-one moiety *via* the methylene linker **3b–d** (**Scaffold 1**) to ensure stable binding of thiazolopyrimidine to the enzyme’s active site. The second pathway was performed on compound **2** by inserting various amide linkers **4a–c, 6a–c,** and **7a,b** (**Scaffold 2**). The choice of the variable substituents was based on the following criteria: a) The amino group, which acts as a hydrogen bond donor, and the carbonyl oxygen, which acts as a hydrogen bond acceptor, could form additional bindings with the receptor (**6a-c**); b) The relatively high lipophilicity of substituted phenyl moieties (**4a-c** and **7a,b**), which can pass nuclear membranes, aiming for strong and selective DNA intercalating activity. Furthermore, all the synthesised compounds possess basic nitrogen atoms that can be protonated at physiological pH to form cationic centres, increasing the affinity and selectivity of these compounds. Moreover, some significant features, including ethyl carboxylate functionality, a phenyl ring substituted with methoxy groups, and the presence of a linker, were preserved to discover more potent and selective Topo II inhibitors and anticancer agents.

## 2. Experimental

### 2.1. Chemistry

Chemicals and reagents were purchased from Aldrich (Sigma-Aldrich) and used without further purification. TLC was used to monitor the reactions, which were carried out on silica gel glass plates containing 60 GF-254 and visualised with UV light or an iodine indicator. Shimadzu IR 435 spectrophotometer (KBr, cm^−1^) was used to determine IR spectra. For structural elucidation of synthesised compounds, ^1^H-NMR (400 MHz) and ^13^C-NMR (100 MHz) spectra were recorded on the Bruker spectrophotometers. Deuterated dimethylsulphoxide (DMSO-d*_6_*) was used as a solvent, and TMS as an internal reference. Chemical shifts were quantified on δ scale in a ppm at the Micro Analytical Centre, Faculty of Pharmacy, Cairo University, Egypt. The synthesised compounds’ mass spectra and elemental analyses were done in the regional centre for mycology and biotechnology at Al-Azhar University on Shimadzu Qp-2010 plus spectrometer. The results agree with the calculations based on the calculated values within the experimental error. The melting points (M.P) of the synthesised compounds were evaluated on the Stuart apparatus, which was uncorrected.

#### 2.1.1. Synthesis of ethyl 4–(2,4-dimethoxyphenyl)-6-methyl-2-thioxo-1,2,3,6-tetrahydropyrimidine-5-carboxylate (1)

A mixture of ethyl acetoacetate (1.3 g, 10 mmol), 2,4-dimethoxybenzaldehyde (1.66 g, 10 mmol), thiourea (1.14 g, 15 mmol), and zinc chloride (0.27 g, 2 mmol) was fused at 80 °C for 4 h in the presence of glacial acetic acid (2 ml). Upon completion of the reaction, the mixture became turbid, and the product solidified. Then, cold aqueous ethanol of 40% (3–5 ml) was added, and the resulting mixture was stirred for a few minutes in an ice bath. The product was filtered, dried, and crystallised from ethanol giving compound **1**.

Yield 85%, mp 145–147 °C, **IR** (KBr, cm^−1^): 3313, 3295 (two NH), 3078 (C-H aromatic), 2943–2889 (C-H aliphatic), 1731 (C = O), 1604 (C = N), 1570 (C = C). **^1^H-NMR** (DMSO-*d_6_*, 400 MHz, *δ* ppm)**:** 1.06 (t, 3H, *J* = 7.08 Hz, OCH_2_CH_3_), 2.29 (s, 3H, CH_3_), 3.74, 3.80 (2s, 6H, two OCH_3_), 3.96 (q, 2H, *J* = 7.08 Hz, OCH_2_CH_3_), 5.42 (s, 1H, C_4_-H), 6.46 (d, 1H, *J* = 8.44 Hz, 2,4-(OCH_3_)_2_-C_6_H_3_-C_5_-H), 6.55 (s, 1H, 2,4-(OCH_3_)_2_-C_6_H_3_-C_3_-H), 6.93 (d, 1H, *J* = 8.44 Hz, 2,4-(OCH_3_)_2_-C_6_H_3_-C_6_-H), 9.18 (s, 1H, N_3_-H, D_2_O exchangeable), 10.18 (s, 1H, N_1_-H, D_2_O exchangeable). **^13^C-NMR** (DMSO-*d_6_*, 100 MHz, *δ* ppm): 14.43 (CH_3_), 17.47 (OCH_2_CH_3_), 49.51 (OCH_2_CH_3_), 55.66, 55.96 (two OCH_3_), 59.79 (C-_6_), 98.76, 100.09, 105.02, 123.70, 128.96, 145.34, 158.14, 160.69 (ArCs), 165.69 (C = O), 174.45 (C = S). **Anal. Calcd.** (%) for C_16_H_20_N_2_O_4_S (336): C, 57.13, H, 5.99, N, 8.33. **Found**: C, 57.40, H, 6.03, N, 8.25.

#### 2.1.2. Synthesis of ethyl 3-amino-5–(2,4-dimethoxyphenyl)-7-methyl-5*H*-thiazolo[3,2-*a*] pyrimidine-6-carboxylate (2)

A solution of pyrimidine derivative **1** (0.34 g, 1 mmol) and chloroacetonitrile (0.11 g, 1.5 mmol) in *N,N*-dimethylformamide (15 ml) was heated under reflux for 10 h. After cooling, the reaction mixture was poured into ice-cold water. The formed solid was filtered, dried, and crystallised from ethanol.

Yield 70%, mp 172–174 °C, **IR** (KBr, cm^−1^): 3420, 3230 (NH_2_), 3080 (C-H aromatic), 2930 (C-H aliphatic), 1733 (C = O), 1604 (C = N), 1575 (C = C). **^1^H-NMR** (DMSO-*d_6_*, 400 MHz, *δ* ppm)**:** 1.15 (t, 3H, *J* = 7.08 Hz, OCH_2_CH_3_), 2.29 (s, 3H, CH_3_), 3.73, 3.80 (2s, 6H, two OCH_3_), 4.01 (q, 2H, *J* = 7.08 Hz, OCH_2_CH_3_), 6.08 (s, 1H, C_5_-H), 6.47–6.48 (m, 1H, 2,4-(OCH_3_)_2_-C_6_H_3_-C_5_-H), 6.52 (s, 1H, 2,4-(OCH_3_)_2_-C_6_H_3_-C_3_-H), 7.24 (s, 2H, NH_2_, D_2_O exchangeable), 7.34 (d, 1H, *J* = 8.16 Hz, 2,4-(OCH_3_)_2_-C_6_H_3_-C_6_-H), 7.77 (s, 1H, thiazole-C_2_-H). **^13^C-NMR** (DMSO-*d_6_*, 100 MHz, *δ* ppm): 14.32 (CH_3_), 18.91 (OCH_2_CH_3_), 52.60 (OCH_2_CH_3_), 55.60, 56.54 (two OCH_3_), 60.19 (C-_5_), 99.05 (C-_2_), 105.13, 106.83, 114.59, 116.61, 120.94, 127.32, 131.97, 151.12, 158.98, 160.96 (ArCs), 165.74 (C = O). **Anal. Calcd.** (%) for C_18_H_21_N_3_O_4_S (375): C, 57.58, H, 5.64, N, 11.19. **Found**: C, 57.62, H, 5.82, N, 11.30.

#### 2.1.3. Synthesis of compounds (3a-d)

##### 2.1.3.1. Ethyl 5–(2,4-dimethoxyphenyl)-7-methyl-3-oxo-3,5-dihydro-5*H*-thiazolo[3,2-*a*] pyrimidine-6-carboxylate (3a)

An equimolar mixture of pyrimidine derivative **1** (0.34 g, 1 mmol) and monochloroacetic acid (0.14 g, 1.5 mmol) was heated under reflux in glacial acetic acid (10 ml) containing anhydrous sodium acetate (0.12 g, 1.5 mmol) and acetic anhydride (3 ml) for 6 h. After cooling, the resulting solid product was filtered and crystallised from ethanol.

Yield 65%, mp 195–197 °C, **IR** (KBr, cm^−1^): 3080 (C-H aromatic), 2930 (C-H aliphatic), 1688, 1733 (2 C = O), 1610 (C = N), 1595 (C = C). **^1^H-NMR** (DMSO-*d_6_*, 400 MHz, *δ* ppm)**:** 1.15 (t, 3H, *J* = 7.12 Hz, OCH_2_CH_3_), 2.32 (s, 3H, CH_3_), 3.74, 3.80 (2s, 6H, two OCH_3_), 3.82 (s, 2H, CH_2_), 4.04 (q, 2H, *J* = 7.12 Hz, OCH_2_CH_3_), 5.94 (s, 1H, C_5_-H), 6.51 (d, 1H, *J* = 8.44 Hz, 2,4-(OCH_3_)_2_-C_6_H_3_-C_5_-H), 6.56 (s, 1H, 2,4-(OCH_3_)_2_-C_6_H_3_-C_3_-H), 7.16 (d, 1H, *J* = 8.44 Hz, 2,4-(OCH_3_)_2_-C_6_H_3_-C_6_-H). **^13^C-NMR** (DMSO-*d_6_*, 100 MHz, *δ* ppm): 14.33 (CH_3_), 19.46 (OCH_2_CH_3_), 34.24 (CH_2_), 53.88 (OCH_2_CH_3_), 55.81, 56.14 (two OCH_3_), 60.82 (C-_5_), 99.27, 105.39, 106.50, 119.22, 120.17, 130.38, 132.51, 159.36, 161.51 (ArCs), 164.73, 170.90 (two C = O). **Anal. Calcd.** (%) for C_18_H_20_N_2_O_5_S (376): C, 57.43, H, 5.36, N, 7.44. **Found**: C, 57.60, H, 5.55, N, 7.35.

##### 2.1.3.2. Ethyl 2-(substituted benzylidene)-5–(2,4-dimethoxyphenyl)-7-methyl-3-oxo-3,5-dihydro-5*H*-thiazolo[3,2-*a*]pyrimidine-6-carboxylate (3b-d)

To a solution of pyrimidine derivative **1** (0.34 g, 1 mmol), the selected aromatic aldehyde (1 mmol) and monochloroacetic acid (0.1 g, 1 mmol) in glacial acetic acid (15 ml) containing anhydrous sodium acetate (0.16 g, 2 mmol) and acetic anhydride (5 ml) were heated under reflux for 6 h. After cooling, the resulting solid product was filtered and crystallised from ethanol.

###### 2.1.3.2.1. Ethyl 5–(2,4-dimethoxyphenyl)-2–(4-methoxybenzylidene)-7-methyl-3-oxo-2,3-dihydro-5*H*-thiazolo[3,2-*a*]pyrimidine-6-carboxylate (3b)

Yield 72%, mp 175–177 °C, **IR** (KBr, cm^−1^): 3060 (C-H aromatic), 2910 (C-H aliphatic), 1685, 1740 (2 C = O), 1612 (C = N), 1585 (C = C). **^1^H-NMR** (DMSO-*d_6_*, 400 MHz, *δ* ppm)**:** 1.16 (t, 3H, *J* = 7.08 Hz, OCH_2_CH_3_), 2.29 (s, 3H, CH_3_), 3.73, 3.80 (2s, 6H, two OCH_3_), 3.83 (s, 3H, OCH_3_), 4.02 (q, 2H, *J* = 7.08 Hz, OCH_2_CH_3_), 6.10 (s, 1H, C_5_-H), 6.49 (d, 1H, *J* = 8.40 Hz, 2,4-(OCH_3_)_2_-C_6_H_3_-C_5_-H), 6.52 (s, 1H, 2,4-(OCH_3_)_2_-C_6_H_3_-C_3_-H), 7.10 (d, 2H, *J* = 8.80 Hz, 4-(OCH_3_)-C_6_H_4_-C_3,5_-H), 7.16 (d, 1H, *J* = 8.40 Hz, 2,4-(OCH_3_)_2_-C_6_H_3_-C_6_-H), 7.55 (d, 2H, *J* = 8.80 Hz, 4-(OCH_3_)-C_6_H_4_-C_2,6_-H), 7.66 (s, 1H, methine-CH). **^13^C-NMR** (DMSO-*d_6_*, 100 MHz, *δ* ppm): 14.38 (CH_3_), 22.81 (OCH_2_CH_3_), 53.05 (OCH_2_CH_3_), 55.65, 55.96, 56.05 (three OCH_3_), 60.34 (C-_5_), 99.17, 105.25, 107.66, 115.41, 117.14, 120.59, 125.89, 131.98, 132.46, 149.82, 150.93 (methine-C), 155.75, 159.05, 161.11, 161.51 (ArCs), 164.74, 165.73 (two C = O). **Anal. Calcd.** (%) for C_26_H_26_N_2_O_6_S (494): C, 63.14, H, 5.30, N, 5.66. **Found**: C, 63.31, H, 5.55, N, 5.80.

###### 2.1.3.2.2. Ethyl 2–(2,4-dimethoxybenzylidene)-5–(2,4-dimethoxyphenyl)-7-methyl-3-oxo-2,3-dihydro-5*H*-thiazolo[3,2-*a*]pyrimidine-6-carboxylate (3c)

Yield 65%, mp 200–202 °C, **IR** (KBr, cm^−1^): 3060 (C-H aromatic), 2910 (C-H aliphatic), 1685, 1740 (C = O), 1612 (C = N), 1585 (C = C). **^1^H-NMR** (DMSO-*d_6_*, 400 MHz, *δ* ppm)**:** 1.15 (t, 3H, *J* = 7.10 Hz, OCH_2_CH_3_), 2.29 (s, 3H, CH_3_), 3.70, 3.73 (2s, 6H, two OCH_3_), 3.84, 3.86 (2s, 6H, two OCH_3_), 4.01 (q, 2H, *J* = 7.10 Hz, OCH_2_CH_3_), 6.07 (s, 1H, C_5_-H), 6.48 (d, 1H, *J* = 8.44 Hz, 2,4-(OCH_3_)_2_-C_6_H_3_-C_5_-H), 6.52 (s, 1H, 2,4-(OCH_3_)_2_-C_6_H_3_-C_3_-H), 6.67 (s, 1H, 2,4-(OCH_3_)_2_-benzylidene-C_6_H_3_-C_3_-H), 6.71 (d, 1H, *J* = 8.64 Hz, 2,4-(OCH_3_)_2_-benzylidene-C_6_H_3_-C_5_-H), 7.14 (d, 1H, *J* = 8.44 Hz, 2,4-(OCH_3_)_2_-C_6_H_3_-C_6_-H), 7.55 (d, 1H, *J* = 8.64 Hz, 2,4-(OCH_3_)_2_-benzylidene-C_6_H_3_-C_6_-H), 7.78 (s, 1H, methine-CH). **^13^C-NMR** (DMSO-*d_6_*, 100 MHz, *δ* ppm): 14.37 (CH_3_), 22.83 (OCH_2_CH_3_), 52.90 (OCH_2_CH_3_), 55.64, 56.12, 56.30, 56.42 (four OCH_3_), 60.29 (C-_5_), 99.08, 105.24, 106.98, 107.48, 114.72, 115.30, 116.90, 120.74, 127.42, 130.88, 131.92, 151.07 (methine-C), 156.14, 159.01, 160.12, 161.08, 163.66 (ArCs), 164.95, 165.76 (two C = O). **Anal. Calcd.** (%) for C_27_H_28_N_2_O_7_S (524): C, 61.82, H, 5.38, N, 5.34. **Found**: C, 61.60, H, 5.44, N, 5.48.

###### 2.1.3.2.3. Ethyl 5–(2,4-dimethoxyphenyl)-7-methyl-3-oxo-2–(3,4,5-trimethoxy benzylidene)-2,3-dihydro-5*H*-thiazolo[3,2-*a*]pyrimidine-6-carboxylate (3d)

Yield 83%, mp 190–192 °C, **IR** (KBr, cm^−1^): 3063 (C-H aromatic), 2905 (C-H aliphatic), 1693, 1735 (2 C = O), 1618 (C = N), 1582 (C = C). **^1^H-NMR** (DMSO-*d_6_*, 400 MHz, *δ* ppm)**:** 1.16 (t, 3H, *J* = 7.12 Hz, OCH_2_CH_3_), 2.29 (s, 3H, CH_3_), 3.70, 3.73 (2s, 9H, three OCH_3_), 3.83 (br. s, 6H, two OCH_3_), 4.01 (q, 2H, *J* = 7.12 Hz, OCH_2_CH_3_), 6.07 (s, 1H, C_5_-H), 6.48 (d, 1H, *J* = 8.44 Hz, 2,4-(OCH_3_)_2_-C_6_H_3_-C_5_-H), 6.53 (s, 1H, 2,4-(OCH_3_)_2_-C_6_H_3_-C_3_-H), 6.86 (s, 2H, 3,4,5-(OCH_3_)_3_-C_6_H_2_-C_2,6_-H), 7.17 (d, 1H, *J* = 8.44 Hz, 2,4-(OCH_3_)_2_-C_6_H_3_-C_6_-H), 7.61 (s, 1H, methine-CH). **^13^C-NMR** (DMSO-*d_6_*, 100 MHz, *δ* ppm): 14.32 (CH_3_), 22.77 (OCH_2_CH_3_), 53.19 (OCH_2_CH_3_), 55.63, 56.03, 56.36 (five OCH_3_), 60.62 (C-_5_), 99.16, 105.22, 107.73, 107.75, 119.33, 120.44, 128.91, 132.07, 132.65, 134.90, 150.84 (methine-C), 153.62, 155.47, 159.08, 161.15 (ArCs), 164.48, 165.64 (two C = O). **Anal. Calcd.** (%) for C_28_H_30_N_2_O_8_S (554): C, 60.64, H, 5.45, N, 5.05. **Found**: C, 60.59, H, 5.55, N, 5.30.

#### 2.1.4. Synthesis of ethyl 3-(substituted benzamido)-5–(2,4-dimethoxyphenyl)-7-methyl-5*H*-thiazolo[3,2-*a*]pyrimidine-6-carboxylate (4a-c)

An equimolar amount of 3-aminothiazolopyrimidine derivative **2** (0.37 g, 1 mmol) and the appropriate aromatic acid chloride (1 mmol) in pyridine (20 ml) was heated under reflux for 5 h. After cooling, the reaction mixture was poured into ice-cooled water. The formed solid was filtered, dried, and crystallised from the appropriate solvent.

##### 2.1.4.1. Ethyl 3–(4-chlorobenzamido)-5–(2,4-dimethoxyphenyl)-7-methyl-5*H*-thiazolo[3,2-*a*]pyrimidine-6-carboxylate (4a)

Crystallised from ethanol, Yield 55%, mp 150–152 °C, **IR** (KBr, cm^−1^): 3320 (NH), 3014 (C-H aromatic), 2912 (C-H aliphatic), 1693, 1735 (2 C = O), 1618 (C = N), 1582 (C = C). **^1^H-NMR** (DMSO-*d_6_*, 400 MHz, *δ* ppm)**:** 1.19 (t, 3H, *J* = 7.12 Hz, OCH_2_CH_3_), 2.29 (s, 3H, CH_3_), 3.74 (br. s, 6H, two OCH_3_), 3.85 (q, 2H, *J* = 7.12 Hz, OCH_2_CH_3_), 5.41 (s, 1H, C_5_-H), 6.48–6.70 (m, 3H, 2,4-(OCH_3_)_2_-C_6_H_3_-C_3_,_5,6_-H), 7.54 (d, 2H, *J* = 8.36 Hz, 4-Cl-C_6_H_4_-C_2_,_6_-H), 7.75 (s, 1H, C_2_-H), 7.83 (d, 2H, *J* = 8.36 Hz, 4-Cl-C_6_H_4_-C_3,5_-H), 10.22 (s, 1H, NH, D_2_O exchangeable). **^13^C-NMR** (DMSO-*d_6_*, 100 MHz, *δ* ppm): 11.47 (CH_3_), 14.50 (OCH_2_CH_3_), 55.67 (OCH_2_CH_3_), 55.88, 56.22 (two OCH_3_), 56.49 (C-_5_), 81.16 (C-_2_), 99.27, 104.87, 107.34, 109.65, 114.42, 121.01, 127.97, 130.64, 130.68, 132.81, 142.41, 149.82, 158.05, 161.34 (ArCs), 166.42, 177.97 (two C = O). **Anal. Calcd.** (%) for C_25_H_24_ClN_3_O_5_S (513): C, 58.42, H, 4.71, N, 8.18. **Found**: C, 58.55, H, 4.93, N, 8.42.

##### 2.1.4.2. Ethyl 3–(2,4-dichlorobenzamido)-5–(2,4-dimethoxyphenyl)-7-methyl-5*H*-thiazolo[3,2-*a*]pyrimidine-6-carboxylate (4b)

Crystallised from isopropanol, Yield 65%, mp 220–222 °C, **IR** (KBr, cm^−1^): 3340 (NH), 3019 (C-H aromatic), 2920 (C-H aliphatic), 1680, 1737 (2 C = O), 1622 (C = N), 1589 (C = C). **^1^H-NMR** (DMSO-*d_6_*, 400 MHz, *δ* ppm)**:** 1.15 (t, 3H, *J* = 7.26 Hz, OCH_2_CH_3_), 2.30 (s, 3H, CH_3_), 3.73, 3.83 (2s, 6H, two OCH_3_), 4.01 (q, 2H, *J* = 7.26 Hz, OCH_2_CH_3_), 6.08 (s, 1H, C_5_-H), 6.48 (d, 1H, *J* = 8.44 Hz, 2,4-(OCH_3_)_2_-C_6_H_3_-C_5_-H), 6.65 (s, 1H, 2,4-(OCH_3_)_2_-C_6_H_3_-C_3_-H), 7.17 (d, 1H, *J* = 8.44 Hz, 2,4-(OCH_3_)_2_-C_6_H_3_-C_6_-H), 7.25 (d, 1H, *J* = 8.40 Hz, 2,4-(Cl)_2_-C_6_H_3_-C_6_-H), 7.57 (d, 1H, *J* = 8.40 Hz, 2,4-(Cl)_2_-C_6_H_3_-C_5_-H), 7.80 (s, 1H, C_2_-H), 7.94 (s, 1H, 2,4-(Cl)_2_-C_6_H_3_-C_3_-H), 10.25 (s, 1H, NH, D_2_O exchangeable). **^13^C-NMR** (DMSO-*d_6_*, 100 MHz, *δ* ppm): 14.33 (CH_3_), 21.45 (OCH_2_CH_3_), 55.99 (OCH_2_CH_3_), 59.76, 60.18 (two OCH_3_), 60.29 (C-_5_), 99.00 (C-_2_), 99.08, 104.99, 105.18, 114.64, 116.72, 125.37, 128.62, 129.10, 129.32, 131.57, 137.79, 149.82, 159.00, 160.08, 161.06, 164.93 (ArCs), 165.74, 167.26 (two C = O). **Anal. Calcd.** (%) for C_25_H_23_Cl_2_N_3_O_5_S (547): C, 54.75, H, 4.23, N, 7.66. **Found**: C, 54.80, H, 4.00, N, 7.83.

##### 2.1.4.3. Ethyl 5–(2,4-dimethoxyphenyl)-7-methyl-3–(2,4,6-trichlorobenzamido)-5*H*-thiazolo[3,2-*a*]pyrimidine-6-carboxylate (4c)

Crystallised from ethanol, Yield 67%, mp 158–160 °C, **IR** (KBr, cm^−1^): 3335 (NH), 3018 (C-H aromatic), 2922 (C-H aliphatic), 1689, 1740 (2 C = O), 1612 (C = N), 1585 (C = C). **^1^H-NMR** (DMSO-*d_6_*, 400 MHz, *δ* ppm)**:** 1.15 (t, 3H, *J* = 7.26 Hz, OCH_2_CH_3_), 2.29 (s, 3H, CH_3_), 3.71, 3.73 (2s, 6H, two OCH_3_), 4.01 (q, 2H, *J* = 7.26 Hz, OCH_2_CH_3_), 6.09 (s, 1H, C_5_-H), 6.47–6.82 (m, 2H, 2,4-(OCH_3_)_2_-C_6_H_3_-C_3_,_5_-H), 7.34 (d, 1H, *J* = 8.44 Hz, 2,4-(OCH_3_)_2_-C_6_H_3_-C_6_-H), 7.70 (s, 2H, 2,4,6-(Cl)_3_-C_6_H_2_-C_3,5_-H), 7.78 (s, 1H, C_2_-H), 10.15 (s, 1H, NH, D_2_O exchangeable). **^13^C-NMR** (DMSO-*d_6_*, 100 MHz*, δ* ppm): 14.35 (CH_3_), 22.81 (OCH_2_CH_3_), 55.55 (OCH_2_CH_3_), 55.63, 59.92 (two OCH_3_), 60.30 (C-_5_), 81.16 (C-_2_), 99.11, 105.20, 106.90, 128.26, 131.05, 131.93, 132.81, 133.45, 136.96, 142.41, 149.82, 157.45, 159.01, 161.08, (ArCs), 165.75, 177.97 (two C = O). **Anal. Calcd.** (%) for C_25_H_22_Cl_3_N_3_O_5_S (581): C, 51.52, H, 3.80, N, 7.21. **Found**: C, 51.20, H, 4.05, N, 6.99.

#### 2.1.5. Synthesis of ethyl 3–(2-chloroacetamido)-5–(2,4-dimethoxyphenyl)-7-methyl-5*H*-thiazolo[3,2-*a*]pyrimidine-6-carboxylate (5)

To a solution of 3-aminothiazolopyrimidine derivative **2** (0.37 g, 1 mmol) in pyridine (20 ml), 2-chloroacetyl chloride (0.112 g, 1 mmol) was added portion wise, and the reaction mixture was heated under reflux for 5 h. The solid product obtained after pouring on ice was filtered, dried, and crystallised from ethanol affording compound **5**.

Yield 80%, mp 150–152 °C, **IR** (KBr, cm^−1^): 3300 (NH), 3082 (C-H aromatic), 2930 (C-H aliphatic), 1730, 1685 (2 C = O), 1610 (C = N), 1585 (C = C). **^1^H-NMR** (DMSO-*d_6_*, 400 MHz, *δ* ppm)**:** 1.16 (t, 3H, *J* = 7.08 Hz, OCH_2_CH_3_), 2.29 (s, 3H, CH_3_), 3.73, 3.83 (2s, 6H, two OCH_3_), 4.01 (q, 2H, *J* = 7.08 Hz, OCH_2_CH_3_), 5.04 (s, 2H, -NHCH_2_COCl), 6.08 (s, 1H, C_5_-H), 6.48–6.80 (m, 2H, 2,4-(OCH_3_)_2_-C_6_H_3_-C_3_,_5_-H), 6.83 (s, 1H, C_2_-H), 6.93 (d, 1H, *J* = 8.24 Hz, 2,4-(OCH_3_)_2_-C_6_H_3_-C_6_-H), 10.20 (s, 1H, NH, D_2_O exchangeable). **^13^C-NMR** (DMSO-*d_6_*, 100 MHz, *δ* ppm): 14.43 (CH_3_), 22.86 (OCH_2_CH_3_), 55.62 (OCH_2_CH_3_), 59.91, 61.36 (two OCH_3_), 67.87 (NHCH_2_COCl), 77.40 (C-_5_), 98.57 (C-_2_), 99.14, 105.03, 106.64, 129.11, 131.92, 132.03, 157.46, 158.15, 160.98, 161.08 (ArCs), 165.75, 167.26 (two C = O). **Anal. Calcd.** (%) for C_20_H_22_ClN_3_O_5_S (451): C, 53.16, H, 4.91, N, 9.30. **Found**: C, 53.33, H, 5.02, N, 9.05.

#### 2.1.6. Synthesis of ethyl 5–(2,4-dimethoxyphenyl)-3-((2-(substituted oxoethyl)amino)-7-methyl-5*H*-thiazolo[3,2-*a*]pyrimidine-6-carboxylate (6a-c)

A mixture of compound **5** (0.45 g, 1 mmol) and the appropriate amine derivative (1 mmol) in absolute ethanol (20 ml) containing a catalytic amount of triethyl amine (2 drops) was heated under reflux for 6 h. The solid formed after cooling was filtered, dried, and crystallised from toluene.

##### 2.1.6.1. Ethyl 5–(2,4-dimethoxyphenyl)-3-((2–(4-ethylpiperazin-1-yl)-2-oxoethyl)amino)-7-methyl-5*H*-thiazolo[3,2-*a*]pyrimidine-6-carboxylate (6a)

Yield 77%, mp 200–202 °C, **IR** (KBr, cm^−1^): 3300 (NH), 3082 (C-H aromatic), 2930 (C-H aliphatic), 1730, 1685 (2 C = O), 1610 (C = N), 1585 (C = C). **^1^H-NMR** (DMSO-*d_6_*, 400 MHz, *δ* ppm)**:** 0.80 (m, 3H, CH_2_CH_3_), 1.19 (t, 3H, *J* = 7.12 Hz, OCH_2_CH_3_), 2.29 (s, 3H, CH_3_), 2.93 (q, 2H, *J* = 6.64 Hz, CH_2_CH_3_), 3.11 (br. s, 4H, piprazinyl-C_3,5_-H), 3.73–3.78 (m, 4H, piprazinyl-C_2,6_-H), 4.20–4.22 (2s, 6H, two OCH_3_), 4.49 (s, 2H, NHCH_2_C = O), 4.50–4.51 (m, 2H, OCH_2_CH_3_), 5.42 (s, 1H, C_5_-H), 6.43–6.62 (m, 2H, 2,4-(OCH_3_)_2_-C_6_H_3_-C_3_,_5_-H), 7.01 (s, 1H, C_2_-H), 7.14–7.26 (m, 1H, 2,4-(OCH_3_)_2_-C_6_H_3_-C_6_-H), 10.19 (s, 1H, NH, D_2_O exchangeable). **^13^C-NMR** (DMSO-*d_6_*, 100 MHz, *δ* ppm): 9.01 (CH_3_), 11.47 (CH_2_CH_3_), 14.35 (OCH_2_CH_3_), 22.64 (NHCH_2_C = O), 41.82, 43.29 (piprazinyl CH_2_ & CH_2_CH_3_), 55.55 (OCH_2_CH_3_), 57.39 (two OCH_3_), 63.73 (C-_5_), 80.91 (C-_2_), 102.09, 109.77, 112.77, 122.11, 123.70, 126.62, 134.79, 140.63, 146.30, 152.14, 154.81, 158.14, 160.69, 165.69 (ArCs), 170.16, 172.99 (two C = O). **Anal. Calcd.** (%) for C_26_H_35_N_5_O_5_S (529): C, 58.96, H, 6.66, N, 13.22. **Found**: C, 58.66, H, 6.42, N, 13.35.

##### 2.1.6.2. Ethyl 5–(2,4-dimethoxyphenyl)-7-methyl-3-((2-morpholino-2-oxoethyl)amino)-5*H*-thiazolo[3,2-*a*]pyrimidine-6-carboxylate (6b)

Yield 62%, mp 210–212 °C, **IR** (KBr, cm^−1^): 3320 (NH), 3090 (C-H aromatic), 2912 (C-H aliphatic), 1739, 1690 (2 C = O), 1614 (C = N), 1585 (C = C). **^1^H-NMR** (DMSO-*d_6_*, 400 MHz, *δ* ppm)**:** 1.16 (t, 3H, *J* = 7.12 Hz, OCH_2_CH_3_), 2.28 (s, 3H, CH_3_), 2.98 (br. s, 4H, morpholinyl-C_3,5_-H), 3.70–3.74 (m, 4H, morpholinyl-C_2,6_-H), 3.78, 3.83 (2s, 6H, two OCH_3_), 3.86 (s, 2H, NHCH_2_C = O), 3.98–4.10 (m, 2H, OCH_2_CH_3_), 5.42 (s, 1H, C_5_-H), 6.43–6.45 (m, 1H, 2,4-(OCH_3_)_2_-C_6_H_3_-C_5_-H), 6.47 (s, 1H, 2,4-(OCH_3_)_2_-C_6_H_3_-C_3_-H), 7.35 (d, 1H, *J* = 8.52 Hz, 2,4-(OCH_3_)_2_-C_6_H_3_-C_6_-H), 7.78 (s, 1H, C_2_-H), 9.10 (s, 1H, NH, D_2_O exchangeable). **^13^C-NMR** (DMSO-*d_6_*, 100 MHz, *δ* ppm): 14.36 (CH_3_), 22.69 (OCH_2_CH_3_), 44.12 (NHCH_2_C = O), 49.52 (morpholinyl-C_3,5_), 52.92 (OCH_2_CH_3_), 55.63, 59.78 (two OCH_3_), 60.28 (morpholinyl-C_2,6_), 62.56 (C-_5_), 80.91 (C-_2_), 99.13, 106.90, 114.70, 116.84, 123.73, 127.37, 131.93, 151.11, 159.01, 161.08 (ArCs), 165.74, 174.48 (two C = O). **Anal. Calcd.** (%) for C_24_H_30_N_4_O_6_S (502): C, 57.36, H, 6.02, N, 11.15. **Found**: C, 57.60, H, 6.33, N, 11.30.

##### 2.1.6.3. Ethyl 5–(2,4-dimethoxyphenyl)-7-methyl-3-((2-oxo-2-(piperazin-1-yl)ethyl)amino)-5*H*-thiazolo[3,2-*a*]pyrimidine-6-carboxylate (6c)

Yield 65%, mp 215–217 °C, **IR** (KBr, cm^−1^): 3305 (NH), 3040 (C-H aromatic), 2918 (C-H aliphatic), 1735, 1680 (2 C = O), 1610 (C = N), 1590 (C = C). **^1^H-NMR** (DMSO-*d_6_*, 400 MHz, *δ* ppm)**:** 1.16 (t, 3H, *J* = 7.12 Hz, OCH_2_CH_3_), 1.94 (s, 1H, CH_3_), 2.26–2.28 (m, 4H, piprazinyl-C_3,5_-H_)_ , 3.09 (s, 2H, NHCH_2_C = O), 3.71–3.73 (m, 4H, piprazinyl-C_2,6_-H), 3.83–3.84. (2s, 6H, two OCH_3_), 4.00–4.02 (m, 2H, OCH_2_CH_3_), 5.41 (s, 1H, C_5_-H), 6.48–6.70 (m, 2H, 2,4-(OCH_3_)_2_-C_6_H_3_-C_3_,_5_-H), 7.79 (s, 1H, C_2_-H), 7.98 (d, 1H, *J* = 8.50 Hz, 2,4-(OCH_3_)_2_-C_6_H_3_-C_6_-H), 8.45 (s, 1H, NH, D_2_O exchangeable), 8.80 (s, 1H, NH, D_2_O exchangeable). **^13^C-NMR** (DMSO-*d_6_*, 100 MHz, *δ* ppm): 11.18 (CH_3_), 14.44 (OCH_2_CH_3_), 46.04 (NHCH_2_C = O), 51.38 (morpholinyl-C_3,5_), 53.38 (OCH_2_CH_3_), 55.63, 60.05 (two OCH_3_), 62.56 (morpholinyl-C_2,6_), 67.87 (C-_5_), 80.41 (C-_2_), 98.42, 99.42, 105.43, 110.27, 115.44, 120.61, 130.29, 140.63, 148.14, 156.14 (ArCs), 166.32, 175.33 (two C = O). **Anal. Calcd.** (%) for C_24_H_31_N_5_O_5_S (501): C, 57.47, H, 6.23, N, 13.96. **Found**: C, 57.60, H, 6.53, N, 13.62.

#### 2.1.7. Synthesis of ethyl 3–(3-(substituted phenyl)ureido)-5–(2,4-dimethoxyphenyl)-7-methyl-5*H*-thiazolo[3,2-*a*]pyrimidine-6-carboxylate (7a,b)

To a solution of compound **2** (0.37 g, 1 mmol) in methylene chloride (5 ml) at 0^°^ C, the selected isocyanate derivative (0.11 mmol) was added dropwise and stirred overnight as the temperature gradually rose to room temperature. Hexane was added to the resulting suspension to complete precipitation. The solid was filtered, dried, and crystallised from ethanol, giving compounds **7a** and **b**.

##### 2.1.7.1. Ethyl 3–(3-(4-chlorophenyl)ureido)-5–(2,4-dimethoxyphenyl)-7-methyl-5*H*-thiazolo[3,2-*a*]pyrimidine-6-carboxylate (7a)

Yield: 80%, mp 180–182 °C, **IR** (KBr, cm^−1^): 3320, 3300 (2 NH), 3014 (C-H aromatic), 2912 (C-H aliphatic), 1693, 1735 (2 C = O), 1618 (C = N), 1582 (C = C). **^1^H-NMR** (DMSO-*d_6_*, 400 MHz, *δ* ppm)**:** 1.16 (t, 3H, *J* = 7.08 Hz, OCH_2_CH_3_), 1.93 (s, 3H, CH_3_), 3.70, 3.73 (2s, 6H, two OCH_3_), 4.12 (q, 2H, *J* = 7.08 Hz, OCH_2_CH_3_), 6.08 (s, 1H, C_5_-H), 6.50 (d, 1H, *J* = 8.56 Hz, 2,4-(OCH_3_)_2_-C_6_H_3_-C_5_-H), 6.65 (s, 1H, 2,4-(OCH_3_)_2_-C_6_H_3_-C_3_-H), 6.71 (d, 1H, *J* = 8.56 Hz, 2,4-(OCH_3_)_2_-C_6_H_3_-C_6_-H), 7.32 (d, 2H, *J* = 8.60 Hz, 4-Cl-C_6_H_4_-C_2,6_-H), 7.48 (d, 2H, *J* = 8.60 Hz, 4-Cl-C_6_H_4_-C_3,5_-H), 7.80 (s, 1H, C_2_-H), 8.99 (s, NH, D_2_O exchangeable), 9.80 (s, NH, D_2_O exchangeable). **^13^C-NMR** (DMSO-*d_6_*, 100 MHz, *δ* ppm): 14.37 (CH_3_), 14.92 (OCH_2_CH_3_), 55.63, 55.95 (two OCH_3_), 60.77 (OCH_2_CH_3_), 67.87 (C-_5_), 80.57 (C-_2_), 99.18, 105.26, 120.05, 120.18, 125.91, 126.37, 129.06, 130.12, 132.29, 138.71, 139.05, 150.30, 152.83, 153.93 (ArCs), 165.65, 176.83 (two C = O). **Anal. Calcd.** (%) for C_25_H_25_ClN_4_O_5_S (528): C, 56.76, H, 4.76, N, 10.59. **Found**: C, 56.44, H, 4.97, N, 10.35.

##### 2.1.7.2. Ethyl 3–(3-(4-chloro-3-(trifluoromethyl)phenyl)ureido)-5–(2,4-dimethoxyphenyl)-7-methyl-5*H*-thiazolo[3,2-*a*]pyrimidine-6-carboxylate (7b)

Yield 75%, mp 200–202 °C, **IR** (KBr, cm^−1^): 3325, 3305 (2 NH), 3030 (C-H aromatic), 2922 (C-H aliphatic), 1685, 1730 (2 C = O), 1612(C = N), 1585 (C = C). **^1^H-NMR** (DMSO-*d_6_*, 400 MHz, *δ* ppm)**:** 1.25 (t, 3H, *J* = 7.08 Hz, OCH_2_CH_3_), 2.24 (s, 3H, CH_3_), 3.84, 3.85 (2s, 6H, two OCH_3_), 3.97 (q, 2H, *J* = 7.08 Hz, OCH_2_CH_3_), 6.10 (s, 1H, C_5_-H), 6.46 (d, 1H, *J* = 8.72 Hz, 2,4-(OCH_3_)_2_-C_6_H_3_-C_5_-H), 6.48– 6.51 (m, 1H, 2,4-(OCH_3_)_2_-C_6_H_3_-C_6_-H), 6.52 (s, 1H, 2,4-(OCH_3_)_2_-C_6_H_3_-C_3_-H), 7.60 (d, 1H, *J* = 8.76 Hz, 3-CF_3_-4-Cl-C_6_H_3_-C_6_-H), 7.67 (d, 1H, *J* = 8.76 Hz, 3-CF_3_-4-Cl-C_6_H_3_-C_5_-H), 8.08 (s, 1H, C_2_-H), 8.09 (s, 1H, 3-CF_3_-4-Cl-C_6_H_3_-C_2_-H), 9.50, 10.10 (2s, 2NH, D_2_O exchangeable). **^13^C-NMR** (DMSO-*d_6_*, 100 MHz, *δ* ppm): 14.38 (CH_3_), 18.78 (OCH_2_CH_3_), 55.59, 55.93 (two OCH_3_), 56.50 (OCH_2_CH_3_), 61.16 (C-_5_), 80.91 (C-_2_), 117.38, 117.44, 121.86, 123.20, 123.69, 124.57 (CF_3_), 126.74, 127.04, 127.35, 127.65, 132.40, 139.37, 152.77, 158.15, 160.85, 164.82, 165.71 (ArCs), 174.44, 176.83 (two C = O), **Anal. Calcd.** (%) for C_26_H_24_ClF_3_N_4_O_5_S (596): C, 52.31, H, 4.05, N, 9.38. **Found**: C, 52.44, H, 4.43, N, 9.03.

### 2.2. Biological screening

#### 2.2.1. Cytotoxicity assay

The MCF7 breast cancer cell line, the A549 non-small cell lung cancer (NSCLC) cell line, and the A498 renal cell carcinoma cell line were the three human tumour tissue lines received from the ATCC. The MTT test was used to evaluate the inhibitory effects of the substances on cell proliferation in the aforementioned cell lines. This colorimetric assay targets the mitochondrial succinate dehydrogenase of living cells to change yellow tetrazolium bromide (MTT) to a purple formazan derivative. Cell lines were cultured in RPMI-1640 medium with 10% foetal bovine serum. The antibiotics of 100 units/mL penicillin and 100 g/mL streptomycin were introduced at 37 °C in an incubator with 5% CO_2_. A 96-well plate with the cell lines was seeded at a density of 1.0 × 10^4^ cells per well for 48 h at 37 °C with 5% CO_2_. Then, the cells were incubated for 24 h and exposed to various chemical concentrations. 20 μL of a 5 mg/mL MTT solution was applied after a 24-h drug treatment period and incubated for 4 h. To dissolve the produced purple formazan, each well receives 100 μL of dimethyl sulfoxide (DMSO). The colorimetric assay is measured and recorded at 570 nm of absorbance using a plate reader (EXL 800, USA). The formula for calculating the relative percentage of cell viability was (A570 of treated samples/A570 of the untreated sample) × 100.

#### 2.2.2. DNA topo II inhibition assay

The ATP-dependent decatenation of kinetoplast DNA (kDNA) obtained from *Crithidia fasciculata*, which consists of a network of numerous minicircles (2.3 Kb) and some maxicircles, was used to measure topoisomerase II inhibition. Reactions took place in 20 μL and comprised topoisomerase II, 200–300 ng of kDNA, and 120 mM KCl with 50 mM Tris-HCl, pH 8, 10 mM MgCl_2_, 0.5 mM ATP, and 0.5 mM dithiothreitol. In preliminary tests, the quantity of topoisomerase II (5 units) was changed to decatenate almost 100% of the kDNA under these assay circumstances. After 30 min of incubation at 37 °C, the reaction samples were stopped by adding 2 ml of a stop buffer containing 10% (w/v) SDS and 2 μL of 0.5 mg/mL proteinase-K and incubated at this temperature for 10 min. The products in the reaction mixture were separated by a 1% agarose gel after the reaction was complete and then could be seen after being stained with ethidium bromide (0.2 μg/mL). The gels were run at 100 V for about 40 min and visualised under UV transillumination.

#### 2.2.3. *In vitro* cytotoxicity on WI-38 human cell line

Cell line cells were provided from the American Type Culture Collection cultured in DMEM (Invitrogen/Life Technologies) with 10% FBS (Hyclone), 1% penicillin-streptomycin, and 10 μg/mL insulin (Sigma). The remainder of the chemicals and reagents were provided by either Sigma or Invitrogen. Prior to the MTT assay, plated cells were incubated for 24 h in a 96-well plate with 100 μl of the test substance per well and 100 μl of complete growth medium (cell density 1.2–1.8 × 10,000 cells/well). Cultures were transferred from the incubator and placed in a sterile work area or laminar flow hood. Then, 10% of the culture medium volume of reconstituted MTT was added, and the cultures were incubated for an additional 2 h. After that, cultures were taken out of the incubator, and the formazan crystals were dissolved using MTT solubilising solution [M-8910] in an amount equal to the volume of the original culture medium. The absorbance at 570 nm was measured. Finally, the IC_50_ of the test compound compared to the reference was calculated using the GraphPad Prism software.

#### 2.2.4. Cell cycle analysis

Compound **4c** was applied to the **A549** cell culture for 48 h at its IC_50_ concentration. Briefly, a six-well plate containing lung cancer (**A549**) cells was seeded with 1 × 10^5^ cells per well and then incubated for 24 h. 2 nM of compound **4c** or 0.1% DMSO was applied to the cells for 24 h. Following that, cells were gathered and fixed in ice-cold 70% ethanol at 4 °C for 12 h. The cells were then rinsed with cold Phosphate Buffer Saline (PBS) and incubated for 30 min at 37 °C in 0.5 ml of PBS after the ethanol was removed. Propidium iodide was used to stain the cells for 30 min in the dark. DNA content was determined using a flow cytometer.

#### 2.2.5. Annexin V-FITC assay

The **A549** cell culture was treated with an IC_50_ of compound **4c** for 48 h. Each well of a 6-well plate received 1 × 10^5^ conc. lung cancer (**A549**) cells that were then incubated for 24 h. The cells were then exposed for 24 h to 2 nM compound **4c** or (0.1%) DMSO before being harvested, washed with PBS, and stained for 15 min at room temperature in the dark with annexin V-FITC and PI in binding buffer (10 µM HEPES, 140 µM NaCl, and 2.5 µM CaCl_2_ at pH 7.4). The flow cytometer was then used to examine the cells.

#### 2.2.6. Molecular docking study

Molecular docking of the most active compounds was performed using Molecular Operating Environment (MOE, 2014.0901) software. Topoisomerase II alpha protein crystal structure co-crystallised with etoposide (PDB code: 5GWK) was obtained from the Research Collaboration for Structural Bioinformatics (RCSB) protein data bank website (http://www.rcsb.org). The topoisomerase II alpha receptor was prepared for docking studies by removing all the water molecules from the active sites. “Protonate 3D process” was used to add hydrogen atoms to the protein. After calculating the partial charges, the determined pocket was isolated, and the backbone was concealed. Several steps were completed before docking, including 3D protonation of the structures, automatically calculating partial charges, and energy minimisation with MOE until an RMSD gradient of 0.05 kcal mol^−1 ^Å^−1^. Docking validation was achieved by self-docking of the co-crystallised ligand (Etoposide) at the topoisomerase II alpha binding site, followed by docking of the most active compounds in the cytotoxicity study as well as a metabolite of compound **4c** and Doxorubicin as a reference drug together with Etoposide. The RMSD was used to measure the superposition of Etoposide, which was 0.73 Å.

## 3. Results and discussion

### 3.1. Chemistry

**Scheme 1.**
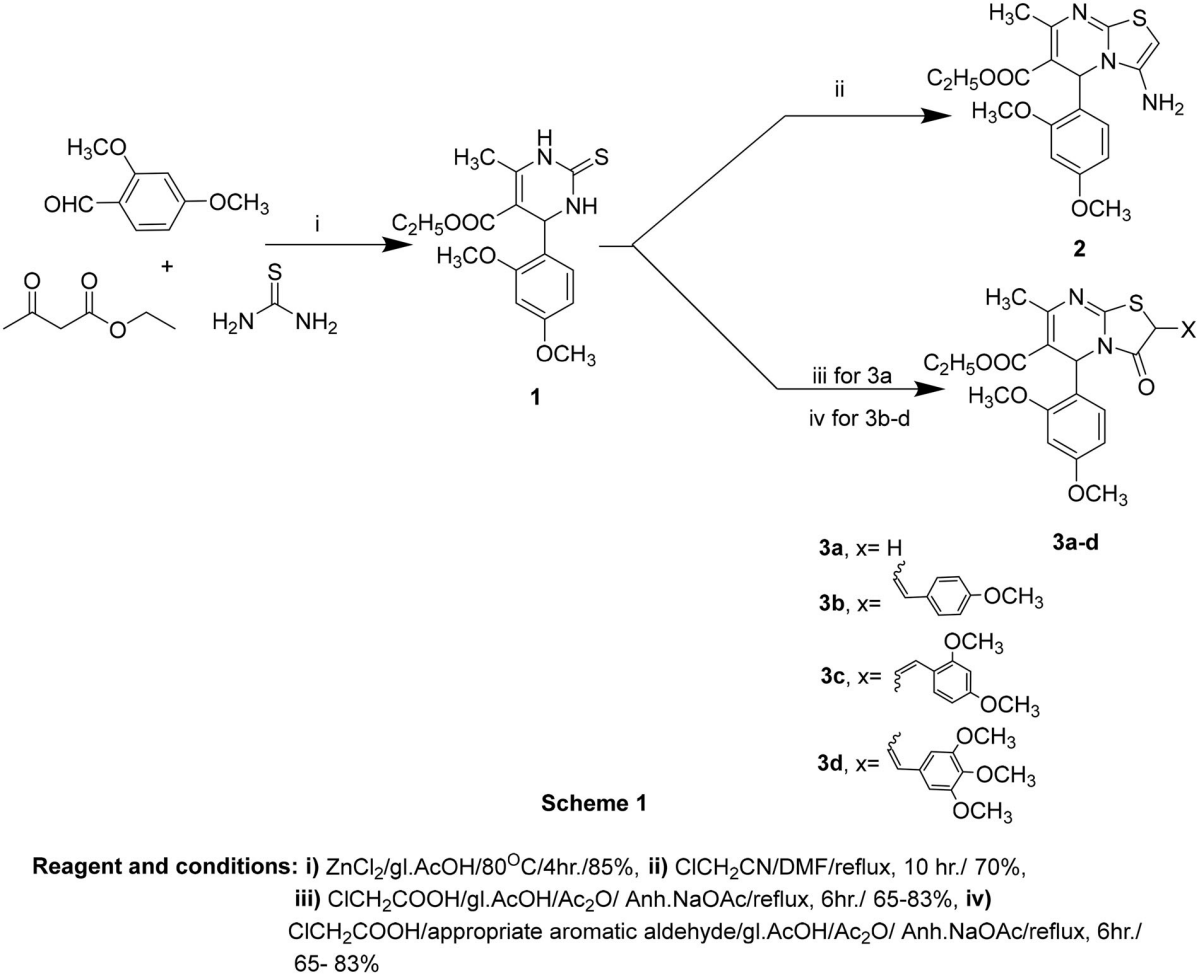
The synthetic pathway for the preparation of the starting material **1** and thiazolo[3,2-*a*]pyrimidines **2** and **3a–d**

**Scheme 2.**
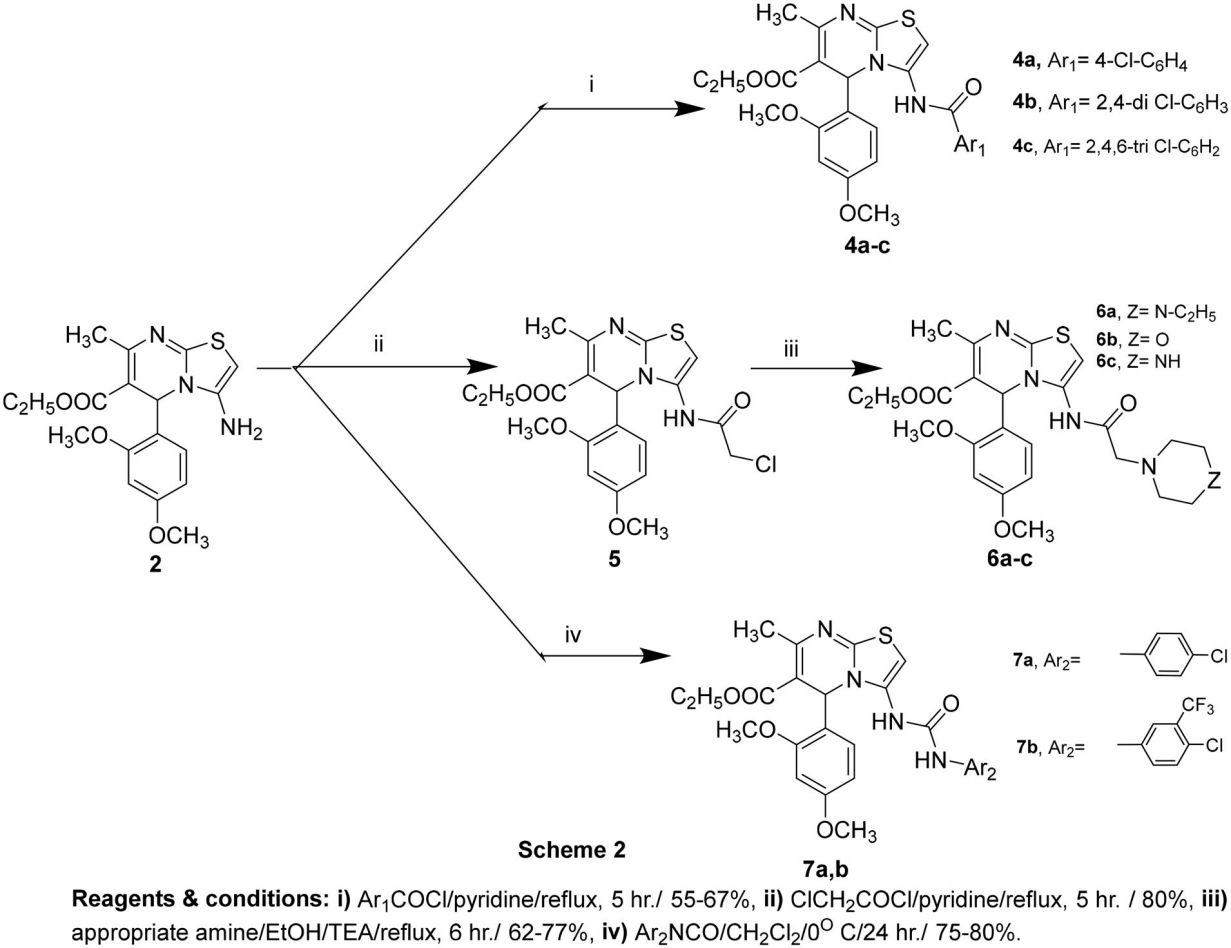
The synthetic approach for the three sets of amide derivatives **4a–c, 5, 6a–c** and **7a,b.**

The synthetic pathways of the target compounds are illustrated in Schemes 1 and 2. 3,4-Dihydropyrimidin-2-(1*H*)-thiones/ones were first synthesised by Pietro Biginelli in 1893 *via* the three-components cyclocondensation reaction of ethyl acetoacetate, benzaldehyde, and thiourea/urea using acid catalysis^26^. After that, such condensations were modified to be achieved under different reaction conditions using various catalysts to improve the yield and minimise the reaction time^27–30^. Hence, compound **1** was synthesised through a solvent-free and one-pot three-component cyclocondensation reaction of ethyl acetoacetate, 2,4-dimethoxybenzaldehyde, and thiourea using a zinc chloride/acetic acid catalytic system^31^, as depicted in Scheme 1. The structure of compound **1** was confirmed by IR, NMR, and elemental analyses. The IR spectrum of compound **1** observed absorption bands at 3313, 3295, and 1731 cm^−1^ corresponding to two NH and carbonyl ester moieties, respectively. ^1^H-NMR spectrum disclosed the presence of triblet and quartette signals at 1.06 and 3.96 ppm attributed to ethyl ester protons in addition to two D_2_O exchangeable singlets at 9.18 and 10.18 ppm corresponding to two NH of pyrimidine nucleus. Furthermore, the existence of signals in ^13^C-NMR at 17.47 and 49.51 for aliphatic ethyl ester carbons with signals at 165.69 and 174.45 ppm for carbonyl and thione carbons confirmed the prepared structure. However, 3-aminothiazolopyrimidine derivatives were reported to be obtained through the reaction of pyrimidine-2-thione scaffolds with choroacetonitrile^32–34^. Accordingly, compound **1** was reacted with chloroacetonitrile in *N,N*-dimethylformamide^33^ to afford 2-aminothiazolopyrimidine **2,** which was assumed to be produced by an electrophilic attack of the tautomeric mercapto group of compound **1** on the active methylene of chloroacetonitrile with the elimination of the hydrogen chloride molecule followed by intramolecular cyclisation *via* the addition of the NH group on the cyano moiety^32^. The structure of compound **2** was confirmed by spectral data. The ^1^H-NMR spectrum showed a D_2_O exchangeable singlet at 7.24 ppm indicative of NH_2_ protons with the presence of a singlet signal at 7.77 ppm characteristic for the thiazole-C_2_-H proton. The ^13^C-NMR spectrum demonstrated signals at their anticipated chemical shifts.

**Figure 3.**
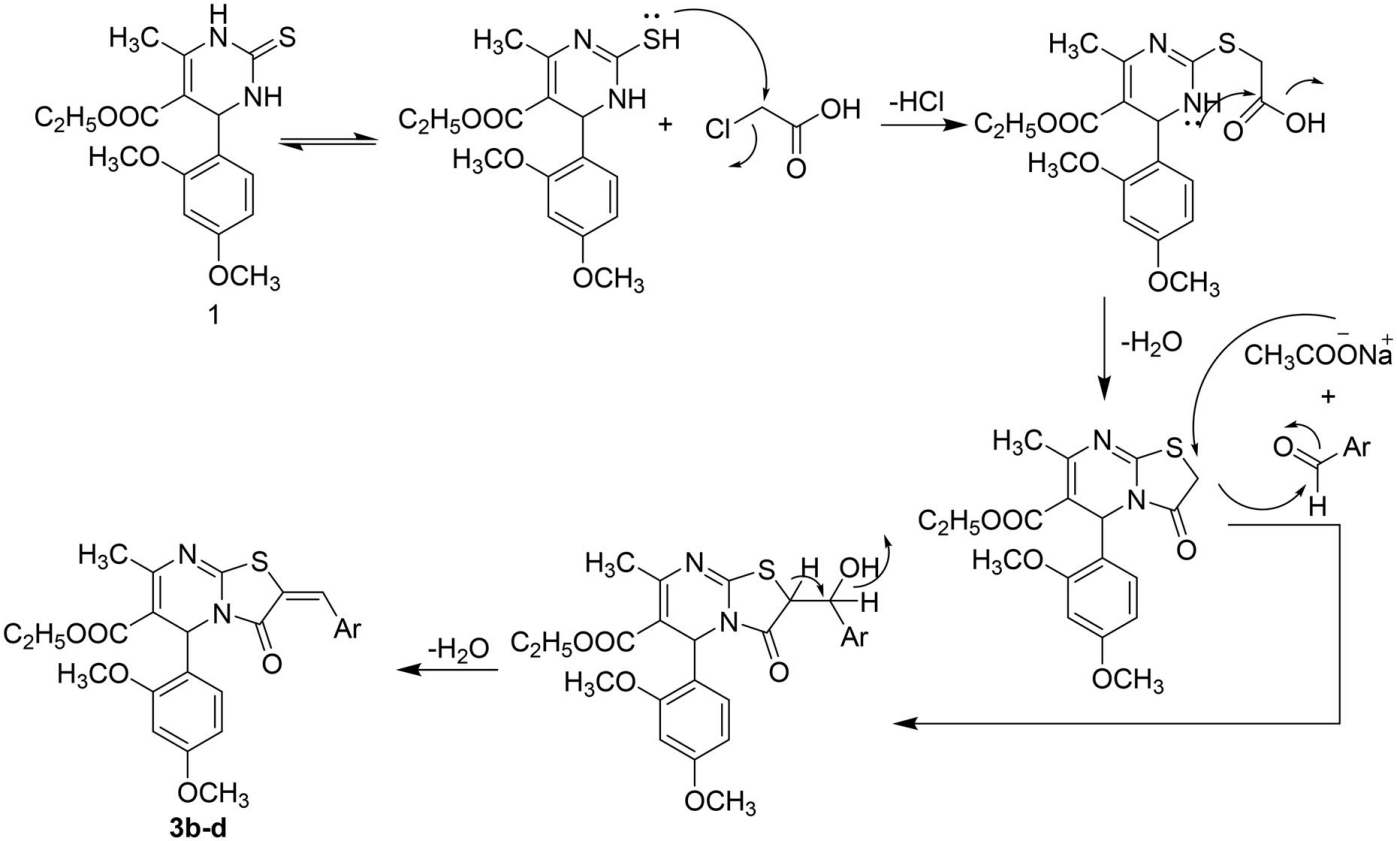
Plausible mechanism for synthesis of compounds **3b–d**.

Moreover, a one-pot three-component condensation of **1** with monochloroacetic acid and appropriate aldehyde in glacial acetic acid containing anhydrous sodium acetate and acetic anhydride^32–35^ resulted in the formation of 2-benzylidene-thiazolopyrimidin-3-one derivatives **3b–d**. However, performing the reaction without aldehyde^35^ resulted in the formation of 3-oxothiazolopyrimidine analog **3a.** The structure of compounds **3a–d** was evidenced through spectral data. The ^1^H-NMR spectrum of compound **3a** showed a singlet signal at 3.82 ppm corresponding to thiazole-CH_2_ protons. While ^1^H-NMR spectra of compounds **3b–d** demonstrated singlet signals at 7.61–7.78 ppm attributed to benzylidene-CH protons. Also, the structures for compounds **3b-d** were confirmed by ^13^C-NMR that revealed signals at 150.84–151.07 ppm, indicating the presence of benzylidene carbons. The reaction was reported^32^ to proceed by a mechanism, as shown in Figure 3.

Several findings^36–41^ proved the biological importance of the amide linker presented in many potential anticancer agents. That stimulated us to synthesise three distinct sets of thiazolopyrimidine with various amide fragments incorporated at the C_3_ position. Accordingly, compound **2** bearing amino functionality at the C_3_ position was used as a starting material for the preparation of new series of the designed compound, as illustrated in Scheme 2.

In this context, a set of 3-substituted benzamidothiazolo[3,2-*a*]pyrimidine-6-carboxylate derivatives **4a–c** was synthesised by the reaction of compound **2** with an appropriate aromatic acid chloride in pyridine^42^. The structures of this set of compounds were evidenced based on spectral data and elemental analyses. The ^1^H-NMR spectra revealed the disappearance of the characteristic NH_2_ signals present in the starting compound **2** and the appearance of D_2_O signals due to amidic NH protons in the range of 10.15–10.25 ppm. Besides, the existence of additional signals for amidic carbonyl carbon in ^13^C-NMR emphasised the proposed structure. Synthesis of the second set of substituted acetamide compounds involved two steps. Firstly, compound **2** was reacted with chloroacetyl chloride in pyridine to afford the chloroacetamido derivative **5**. Secondly, treatment of the latter with the appropriate amines in absolute ethanol containing catalytic amounts of triethyl amine^43^ yielded the target substituted acetamide products **6a-c**. Their structures were assigned based on spectral data. The aliphatic region in the ^1^H-NMR spectra showed additional singlet signals at 3.09–3.89 ppm for compounds **6a–c** and 5.04 ppm for compound **5** attributed to acetamido CH_2_ protons. Also, the ^1^H-NMR spectrum of compound **6c**, as a representative example, showed two multiples at 2.26–2.28 ppm and 3.71–3.73 ppm corresponding to piperazine protons. ^13^C-NMR spectra demonstrated signals at their anticipated chemical shifts. Finally, compound **2** was reacted with the selected isocyanates in methylene chloride^44^ to afford the third set of aminoamide derivatives **7a,b**. All the spectroscopic data of the compounds were in full accordance with their structures. ^1^H-NMR spectra of compounds **7a** and **b** revealed D_2_O exchangeable signals for two NH protons of the ureido moiety at 8.99–10.10 ppm. ^13^C-NMR spectra of compounds **7a** and **b** illustrated the carbons of the incorporated substituted phenyl moieties of isocyanates, as well as the position and the total number of other signals that were consistent with the proposed structures.

### 3.2. Biological screening

#### 3.2.1. Cytotoxicity assay

The newly prepared compounds were screened against three cancer cell lines: namely, breast cancer cell line **MCF7**, non-small cell lung cancer (NSCLC) cell line **A549,** and renal cancer cell line A498, using the MTT colorimetric assay. The choice of these three cell types was based on the over-expression of topoisomerase II in such cells^25^^,^^45–48^. The results of the five-doses cytotoxicity evaluation of the newly synthesised compounds were compared with Doxorubicin as a reference anticancer agent. Table 1 presents the average IC_50_ values for the five doses of cytotoxicity against the chosen cancer cell lines.

**Table 1.** IC_50_ values of the designed targets against MCF7, A549, and A498 cell lines.

Ser	Cytotoxicity IC_50_ (nM)^a^			
	Compound	MCF7± SD	A549± SD	A498± SD
1	1	103 ± 1.62	210 ± 3.61	115 ± 2.3
2	2	41 ± 0.72	23 ± 0.45	332 ± 7.4
3	3a	107 ± 1.88	93 ± 1.79	73 ± 1.63
4	3b	22 ± 0.62	24 ± 0.61	46 ± 1.35
5	3c	16 ± 0.38	19 ± 0.52	9 ± 0.34
6	3d	3 ± 0.1	12 ± 0.38	4 ± 0.15
7	4a	111 ± 2.66	75 ± 1.97	47 ± 1.45
8	4b	56 ± 1.44	48 ± 1.35	113 ± 3.7
9	4c	2 ± 0.05	2 ± 0.07	1 ± 0.05
10	5	101 ± 2.11	31 ± 0.73	38 ± 1.02
11	6a	14 ± 0.36	8 ± 0.24	2 ± 0.07
12	6b	3 ± 0.08	6 ± 0.16	10 ± 0.32
13	6c	68 ± 1.6	19 ± 0.51	25 ± 0.75
14	7a	78 ± 1.93	54 ± 1.46	38 ± 1.19
15	7b	16 ± 0.47	19 ± 0.58	9 ± 0.33
*	Doxorubicin	9 ± 0.26	13 ± 0.42	7 ± 0.23

**^a^**Results are the mean ± SD of two independent assays.

**Figure 4.**
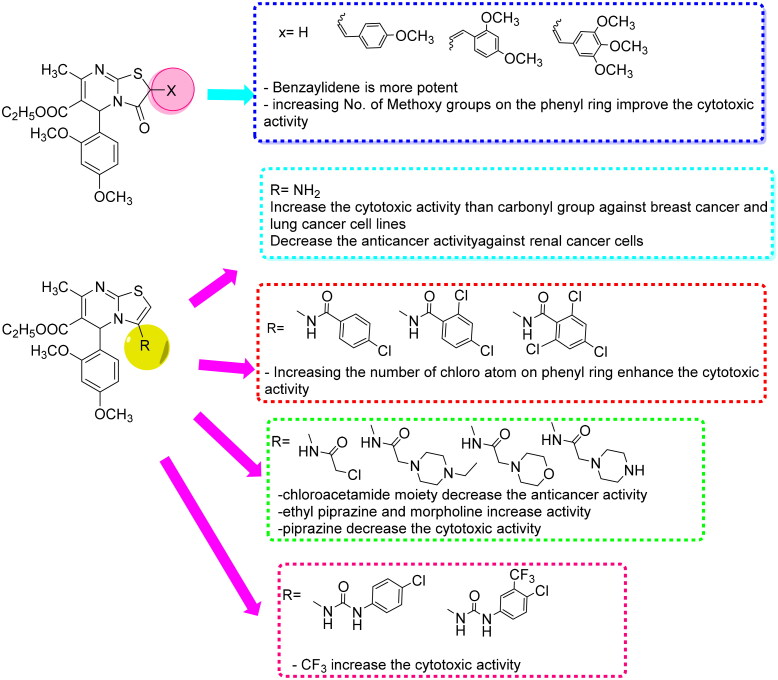
SAR study for the designed thiazolopyrimidines.

According to the tabulated results, most of the tested compounds showed very strong to moderate cytotoxic activity against the three cancer cell lines. However, some compounds demonstrated higher activity than the reference drug, such as compounds **3d**, **4c**, and **6b** against breast cancer cell line with IC_50_ values of 3, 2, and 3 nM, respectively, compared to the IC_50_ of Doxorubicin of 9 nM. Also, compounds **3d**, **4c, 6a,** and **6b** exhibited higher cytotoxic activity against the lung cancer cell line than the reference drug (Doxorubicin IC_50_ = 13 nM), with IC_50_ values of 12, 2, 8, and 6 nM, respectively. Additionally, compounds **3d**, **4c**, and **6a** illustrated higher potency against the renal cancer cell line than Doxorubicin (IC_50_ = 7 nM), having IC_50_ values of 4, 1, and 2 nM, respectively. Among the cancer cells tested, lung cancer cells (**A549**) and renal cancer cells (**A498**) were found to be the most sensitive to the cytotoxic effect of new compounds, followed by breast cancer cells (**MCF-7**). **SAR** evaluation of the tested compounds revealed that the bicyclic thiazolopyrimidines were more effective than the monocyclic pyrimidine backbone **1**. Furthermore, 3-aminothiazolopyrimidine **2** (IC_50_ 41, 23 nM) was found to exhibit better cytotoxic activity than thiazolopyrimidine-3-one **3a** (IC_50_ 107, 93 nM) against breast cancer **MCF-7** and lung cancer cells **A549**, in contrast to renal cancer cell line **A498**, demonstrating that thiazolopyrimidine-3-one **3a** (IC_50_ 73 nM) was more effective than 3-aminothiazolopyrimidine **2** (IC_50_ 332 nM). Moreover, the introduction of a substituted benzylidene moiety at the C_2_ position of the thiazolopyrimidine-3-one scaffold, as seen in compounds **3b–d**, resulted in a significant boost in cytotoxic activity. Additionally, increasing the number of electron-donating substituents on the phenyl ring of the benzylidene moiety led to a notable positive impact on the potency. As a result, 3,4,5-trimethoxybenzylidene derivative **3d** with IC_50_ values of 3, 12, and 4 nM was discovered to be more potent than the 2,4-dimethoxybenzylidene derivative **3c** with IC_50_ values of 16, 19 and 9 nM, which in turn was more potent than 4-methoxybenzylidene analog **3b** with IC_50_ values of 22, 24, and 46 nM against the three cancer cells, **MCF-7**, **A549**, and **A498**, respectively, as found in the literature^24^. After that, three sets of amide derivatives with diverse cytotoxic activities were formed by replacing the 3-amino group with various amide fragments. Among the first set of 3-substituted benzamide moiety compounds, compound **4c** demonstrated potent anticancer activity with IC_50_ values of 2, 1, and 2 nM against three cancer cells, **MCF-7, A549,** and **A498**, respectively, while the remaining compounds of this set exhibited only modest cytotoxic activity. That highlighted the significance of the presence of three electron-withdrawing groups on the phenyl ring. Of the second set of compounds with acetamide fragments at the C_3_ position, compounds **6a** and **6b** bearing 4-ethylpiperazinyl acetamide and morpholinyl acetamide moieties, respectively, demonstrated significant cytotoxic activity, stronger than the reference drug with IC_50_ values of 14, 8, and 2 nM for compound **6a** and 3, 6, and 10 nM for compound **6b** against three cancer cells, **MCF-7, A549,** and **A498**, respectively. In contrast, the introduction of the chloroacetamide and piperazinyl acetamide moieties at the C_3_ position, as in compounds **5** and **6c**, resulted in a remarkable decrease in anticancer activity. Furthermore, compound **7b** from the last set, with an aminoamide fragment bearing a 3-trifluoromethyl-4-chlorophenyl moiety, exhibited excellent cytotoxicity, comparable to Doxorubicin, with IC_50_ values of 19 and 9 nM against cancer A549 and A498 cells, respectively. However, when the electron-withdrawing, 3-trifluoromethyl group, was removed from compound **7a** in the same series, the cytotoxic activity decreased, highlighting the importance of having multiple electron-withdrawing groups on the phenyl side chain, Figure 4.

#### 3.2.2. Topoisomerase II inhibition enzyme assay

**Figure 5.**
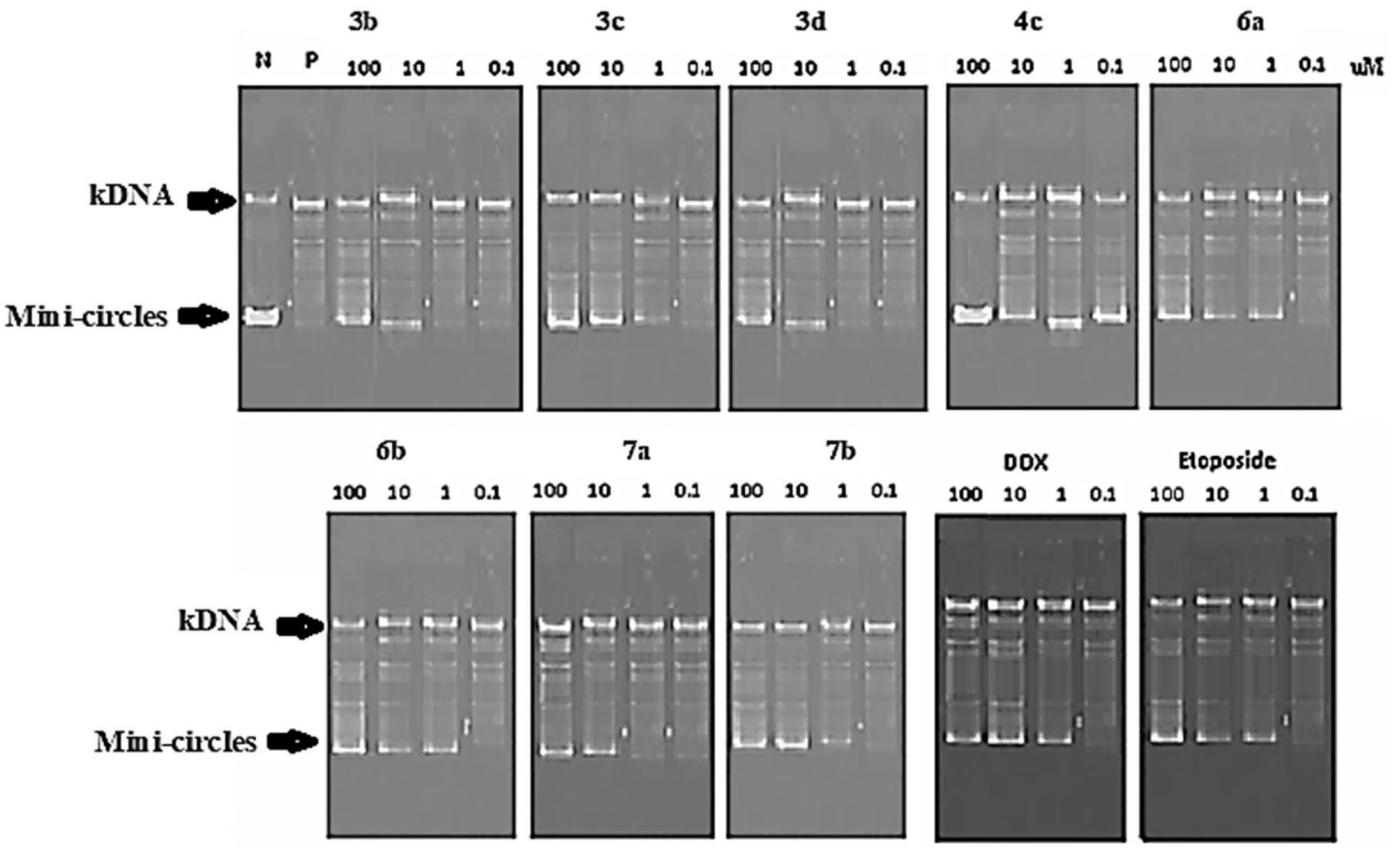
Agarose gel electrophoresis of compounds **3b-3d, 4c, 6a, 6b, 7a** and **7b** with Etoposide and Doxorubicin at 100, 10, 1, 0.1 μM concentrations.

Human topoisomerase II has been demonstrated to be an effective goal in the treatment of a variety of cancers. Topo II enzyme has been reported to be targeted by anticancer drugs such as Etoposide, Doxorubicin, Daunorubicin, and Mitoxantrone^49^. These drugs are classified based on their mechanism of action as intercalating agents (e.g. Doxorubicin) or non-intercalating agents, such as epipodophyllotoxins (e.g. Teniposide)^50^. Therefore, the most active compounds as cytotoxic agents **3b-3d, 4c, 6a, 6b, 7a,** and **7b** were further subjected to enzymatic evaluation to study their inhibitory activity on the Topo II enzyme using human topoisomerase II decatenation assay. This assay is based on the fact that human Topo II can decatenate interlinked double-stranded DNA molecules. The inhibitory activity was evaluated using four concentrations (0.1, 1, 10, 100 μM), and the results were observed on staining gel electrophoresis using Etoposide and Doxorubicin as reference drugs (Figure 5).

The IC_50_ was calculated and illustrated in Table 2. The results revealed that compound **4c** was the most potent with Topo II inhibitory IC_50_ of 0.23 ± 0.01 µM, which was 1.4-fold and 3.6-fold higher than the IC_50_ of Etoposide (0.32 ± 0.02 µM) and Doxorubicin (0.83 ± 0.05 µM) as positive controls, respectively. Whereas compounds **7b, 3c, 6b,** and **6a** showed marked Topo II inhibitory activity with a nearly high potency of Doxorubicin as a positive control, with IC_50_ values ranging from 1.05 to 1.88 µM. The remaining compounds demonstrated mild inhibitory activity. As a result, the outcomes of the Topo II inhibition assay, as well as the cytotoxicity evaluation, reflected the importance of the presence of a trichlorobenzamide side chain at the C_3_ position of the thiazolopyrimidine scaffold.

**Table 2.** IC_50_ of the tested compounds on Topo II in comparison with Etoposide and Doxorubicin.

Compounds	TOPO II IC_50_ (µM)± SD
**3b**	6.14 ± 0.36
**3c**	1.39 ± 0.08
**3d**	2.21 ± 0.13
**4c**	0.23 ± 0.01
**6a**	1.88 ± 0.11
**6b**	1.51 ± 0.09
**7a**	3.56 ± 0.21
**7b**	1.05 ± 0.06
Etoposide	0.32 ± 0.02
Doxorubicin	0.83 ± 0.05

Results are the mean ± SD of two independent assays.

#### 3.2.3. *In vitro* cytotoxicity on WI-38 human cell line

**Figure 6.**
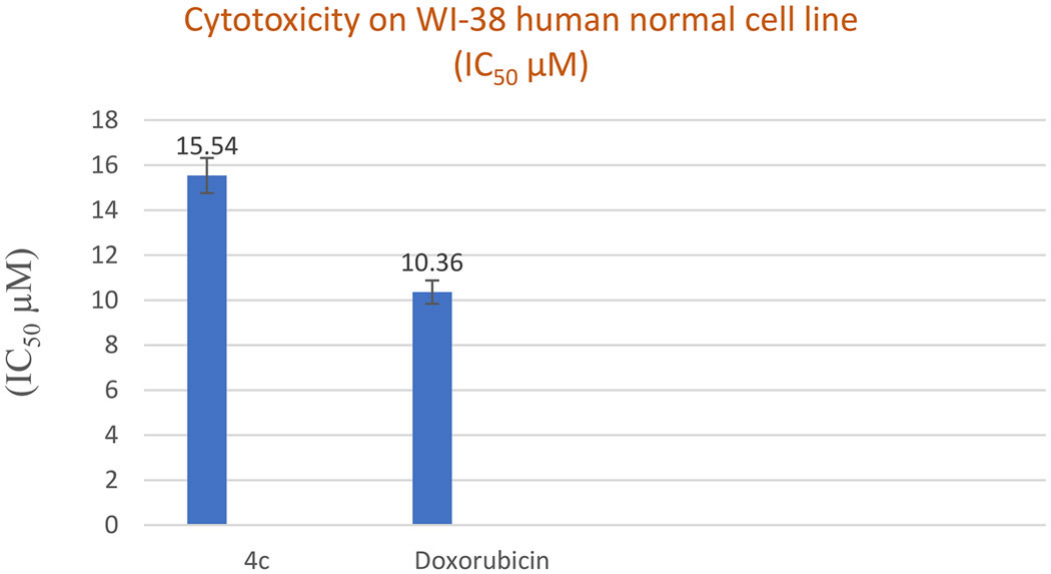
*In vitro* cytotoxicity (IC_50_) of compound **4c** and Doxorubicin on WI-38 human cell line (Normal cell composed of fibroblasts and derived from lung tissue of a 3-month-gestation aborted female fetus).

The most active cytotoxic agent with potent Topo II inhibitory activity **4c** was then tested against the human normal cell line WI-38. As shown in Figure 6, the tested compound **4c** had low cellular cytotoxicity with an IC_50_ value of 15.54 ± 0.84 µM compared to the reference drug Doxorubicin (IC_50_ 10.36 ± 0.56 µM).

#### 3.2.4. Selectivity index

According to Badisa et al.^51^, the Selectivity index (SI) value of the compounds reflects their degree of selectivity. A compound with a high SI value indicates selective toxicity towards cancer cells (SI = IC_50_ normal cell/IC_50_ cancer cell) and little effect on the normal cell line, suggesting that the compounds can be investigated for potentially promising antitumor activity. Accordingly, compound **4c**, which has a higher SI than Doxorubicin by about 7 to 10.5-fold, appears to be the most active thiazolopyrimidine derivative and has a high potential for *in vitro* anticancer activity (Table 3).

**Table 3.** The degree of selectivity of the tested compound **4c** and Doxorubicin.

Selectivity index (SI)^a^			
Compounds	MCF7	A549	A498
**4c**	7770	7770	15540
Doxorubicin	1151.11	796.92	1480

**^a^**SI = IC_50_ normal cell/IC_50_ cancer cell.

#### 3.2.5. Cell cycle analysis

**Figure 7.**
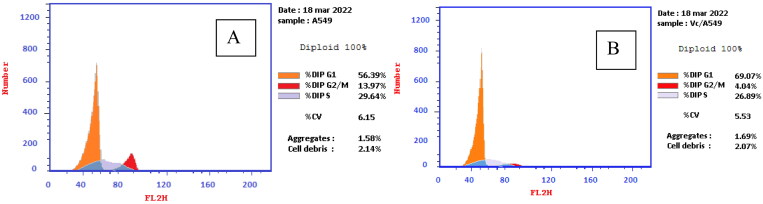
(A) Cell cycle analysis of A549 treated with DMSO only, (B) Cell cycle analysis of A549 after treatment with **4c** for 24 h and 48 h.

The cell cycle is required for cell division and replication. The cell cycle was divided into four distinct phases: G1 phase (synthesis), S phase (synthesis), G2 phase (interphase), and M phase (mitosis). The G1 phase, also known as the post-mitotic pre-synthesis phase, is distinguished by direct cell division. DNA replication identifies the S phase. The G2 phase, premitotic, or post-synthetic phase, which can be considered the actual division, is when the cell prepares to split into two cells. Finally, the doubled DNA organised in chromosomes is separated during the M- or mitosis-phase division^25^. Many anticancer drugs cause apoptosis, cell cycle arrest, or a combination of both as part of their cytotoxic action^52^. As a result, it was worth investigating whether cell cycle arrest was involved in the cytotoxicity mechanism of the most active cytotoxic agent **4c** on A549 cells using flow cytometry analysis, and the results were demonstrated in Table 4 and Figure 7. The results revealed a 69.07% increase in cell count at the G0-G1 phase, compared to 56.39% for control cells. While the percentage of cells in the S phase was reduced by 26.89% compared to the control (29.64%). On the other hand, a dramatic fall in the cell population in the G2/M phase was observed upon treatment with compound **4c** from 13.97 to 4.04%. As a result, compound **4c** was demonstrated to significantly disrupt the cell cycle profile and cause cell cycle arrest.

**Table 4.** Cell cycle analysis of A549 cells after treatment with **4c** and DMSO control.

Compounds	%G0–G1	%S	%G2/M
**4c**	69.07	26.89	4.04
control	56.39	29.64	13.97

#### 3.2.6. Annexin V-FITC apoptosis assay

**Figure 8.**
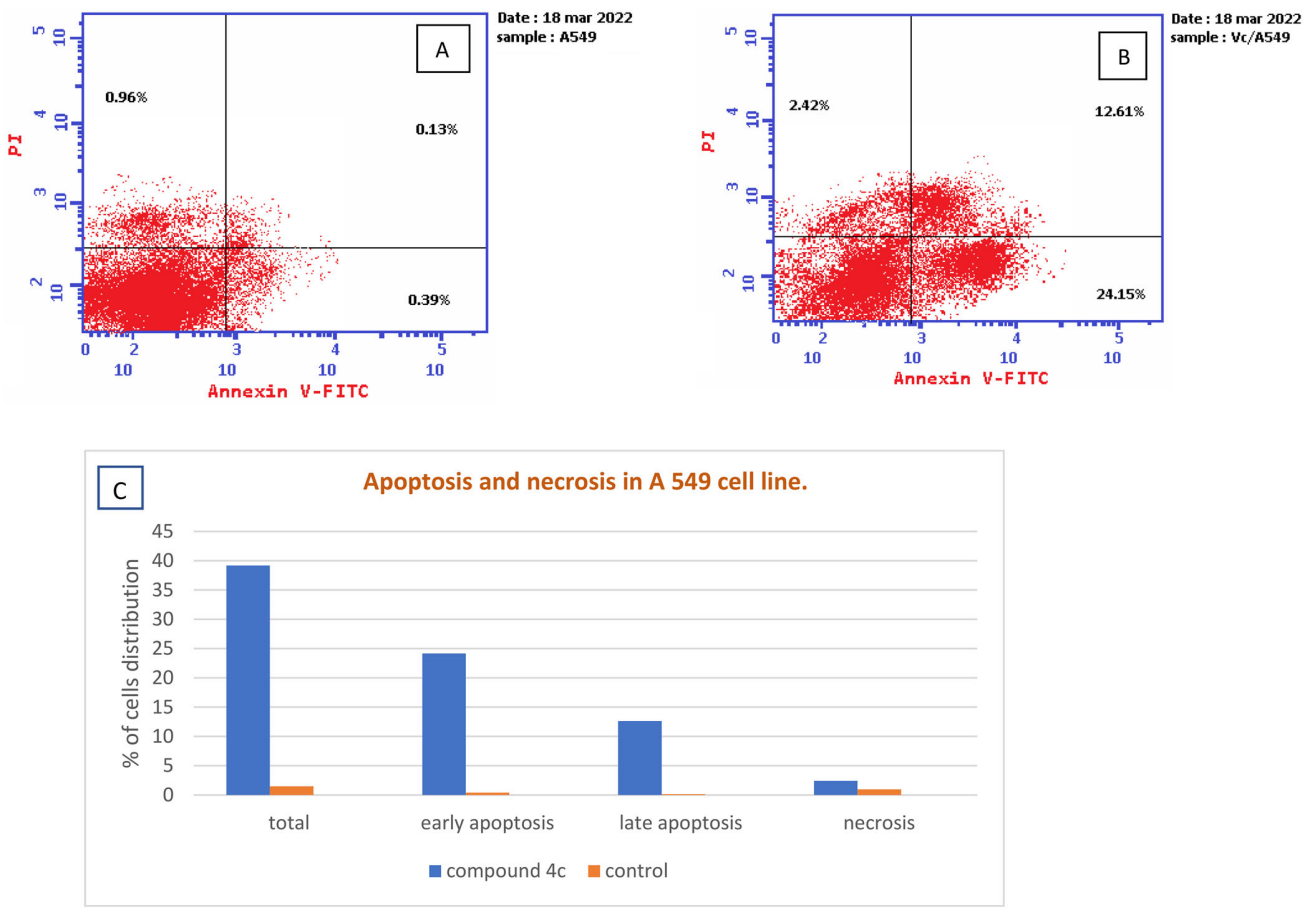
(A) Apoptosis and necrosis in A 549 cell line (control), (B) The effect of compound **4c** on apoptosis and necrosis in A 549 cells, (C) Graphical representation of the effect on apoptosis and necrosis of **4c** on A 549 cell line compared to control cells.

Apoptotic cell death is a method by which an anticancer agent destroys tumour cells^53^. Therefore, compound **4c** was additionally estimated through an Annexin V-FITC experiment utilising A549 cells to evaluate if the cytotoxic action of the novel compound is related to necrosis or apoptosis. The findings (Figure 8(A–C)) revealed a significant increase in early and late apoptotic cells by 61.92-fold (24.15%) and 97-fold (12.61%), compared to control cells with 0.39% and 0.13%, respectively. Additionally, there was an increase in the percentage of necrosis by 2.5-fold, along with an elevation in the total apoptosis by 26.47-fold when compared to control cells. These findings indicated that apoptosis could be a mechanism by which compound **4c** caused cell death in A549 cells.

#### 3.2.7. Molecular docking study

**Figure 9.**
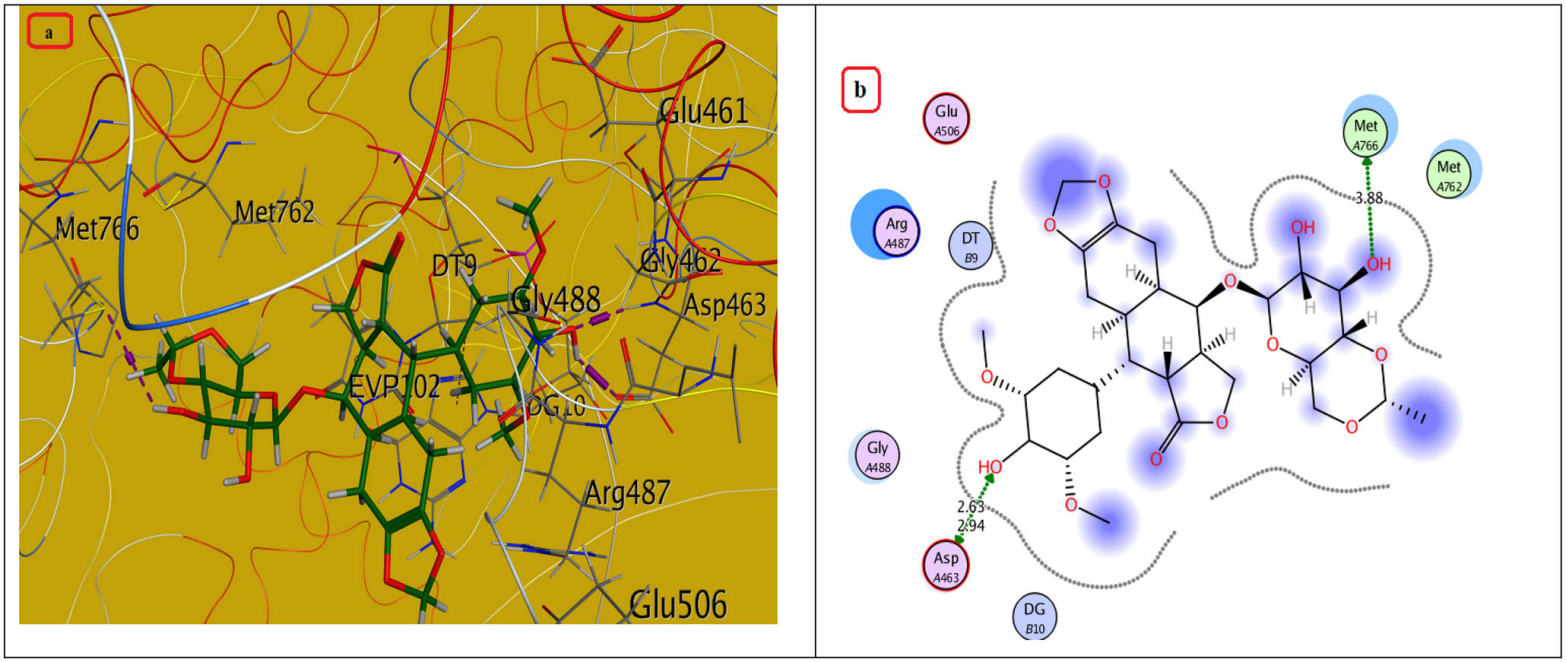
The (a) 3D and (b) 2D interaction mode of the ligand (Etoposide) within topoisomerase II binding pocket (PDB code 4GWK). The pink dot arrow represents H-bond.

**Figure 10.**
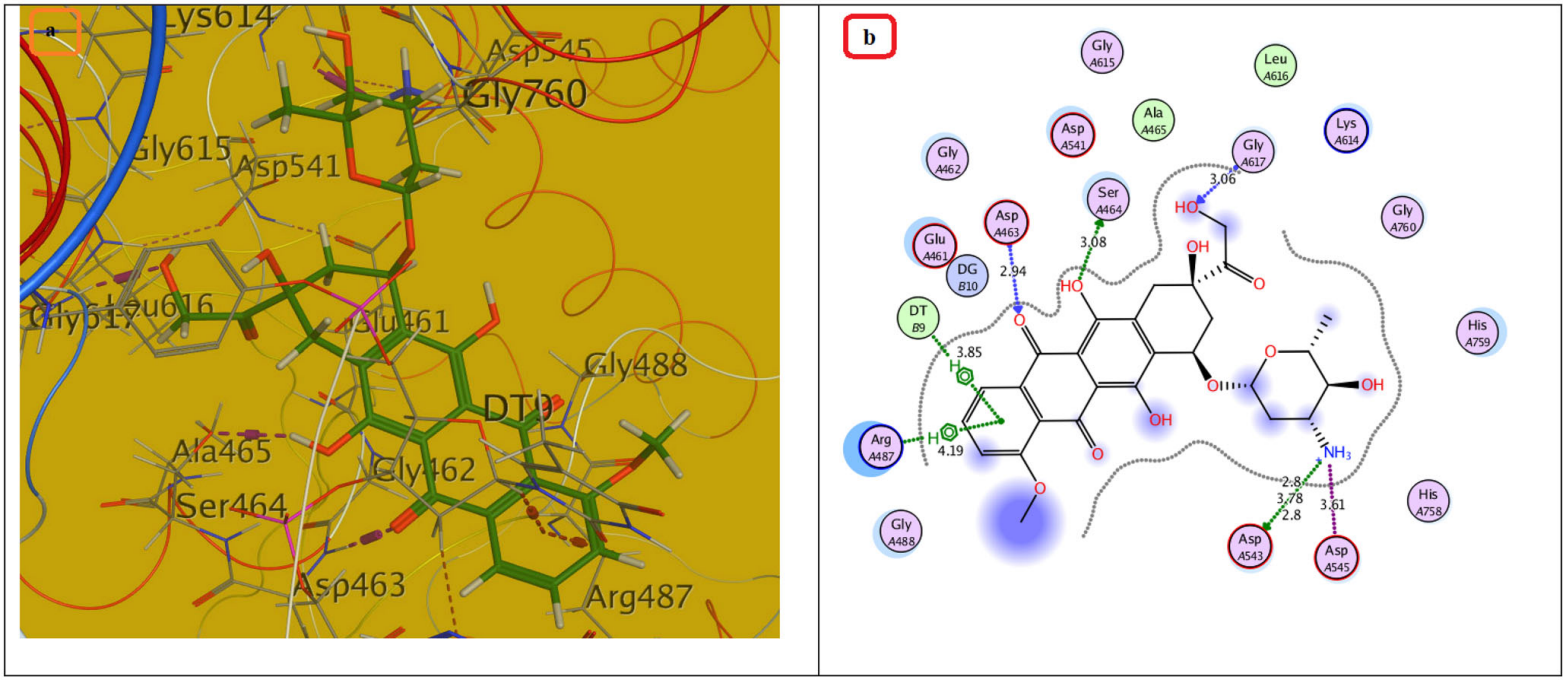
The (a) 3D and (b) 2D interaction mode of Doxorubicin (reference drug) within topoisomerase II binding pocket. The pink dot arrow represents H-bonds and brown dot arrow represents H-π interaction.

**Figure 11.**
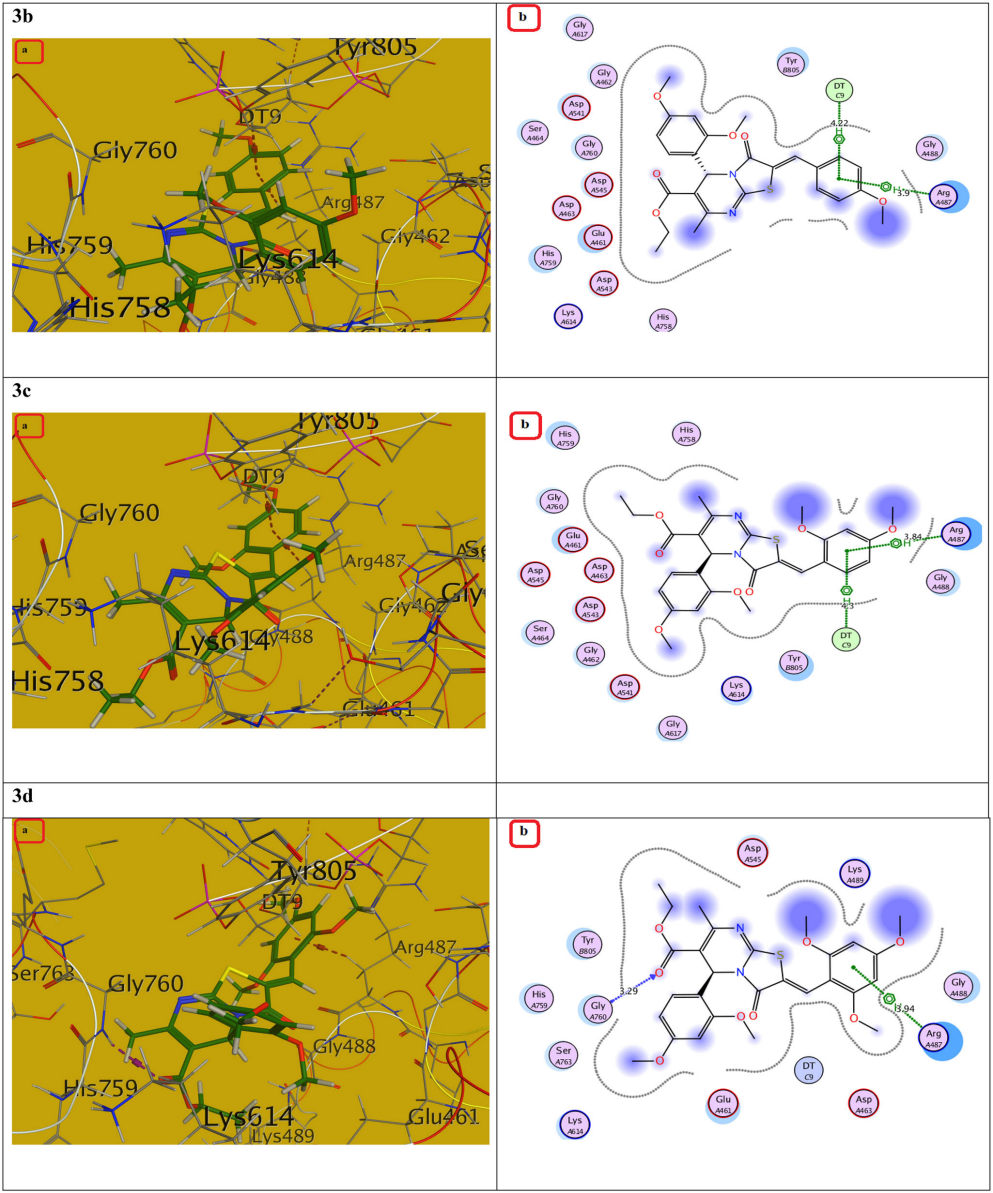
The (a) 3D and (b) 2D interaction mode of compounds **3b-d** within topoisomerase II binding pocket. The pink dot arrow represents H-bonds and brown dot arrow represents H-π interaction.

**Figure 12.**
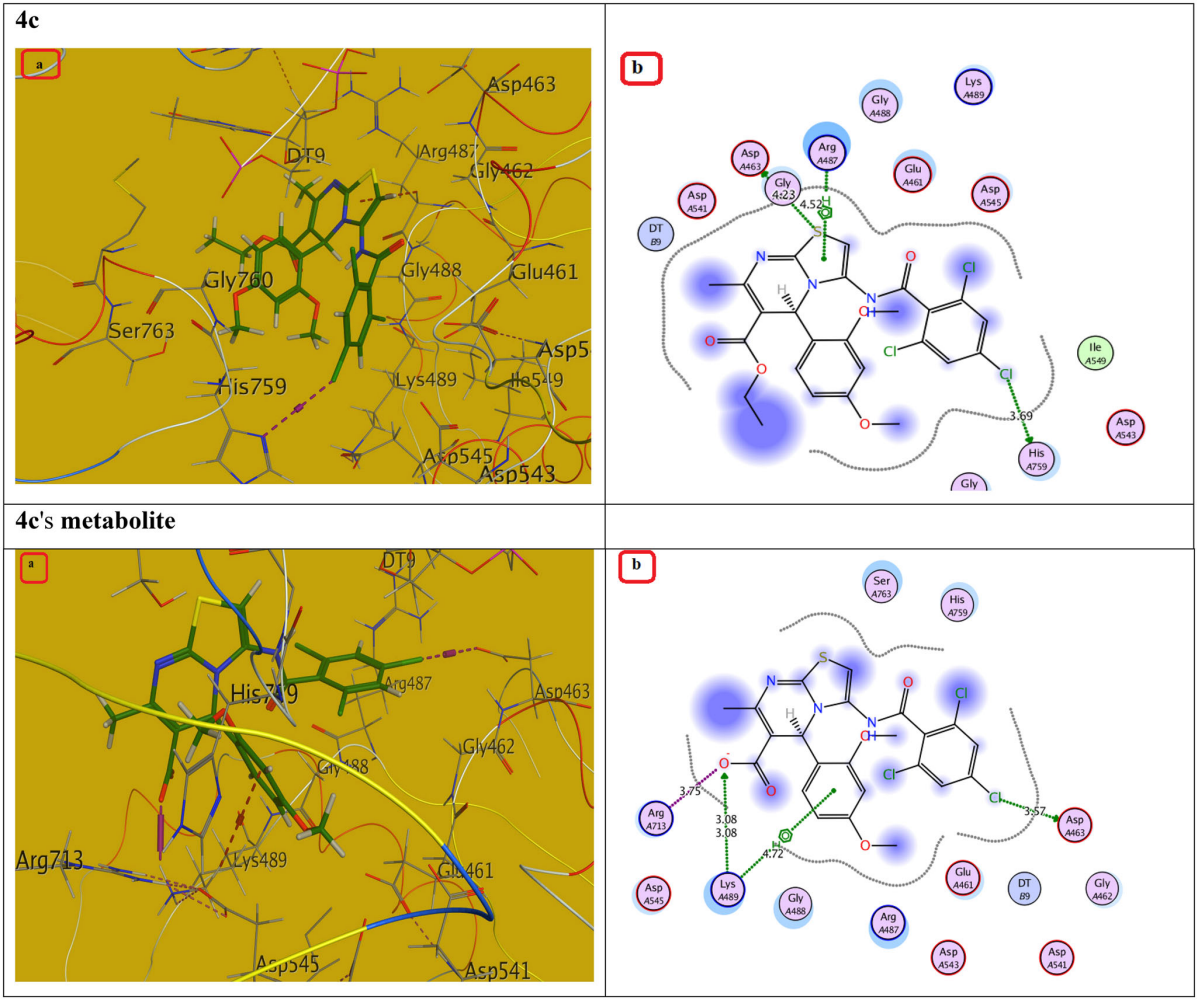
The (a) 3D and (b) 2D interaction mode of compound **4c** and its metabolite within topoisomerase II binding pocket. The pink dot arrow represents H-bonds and brown dot arrow represents H-π interaction.

**Figure 13.**
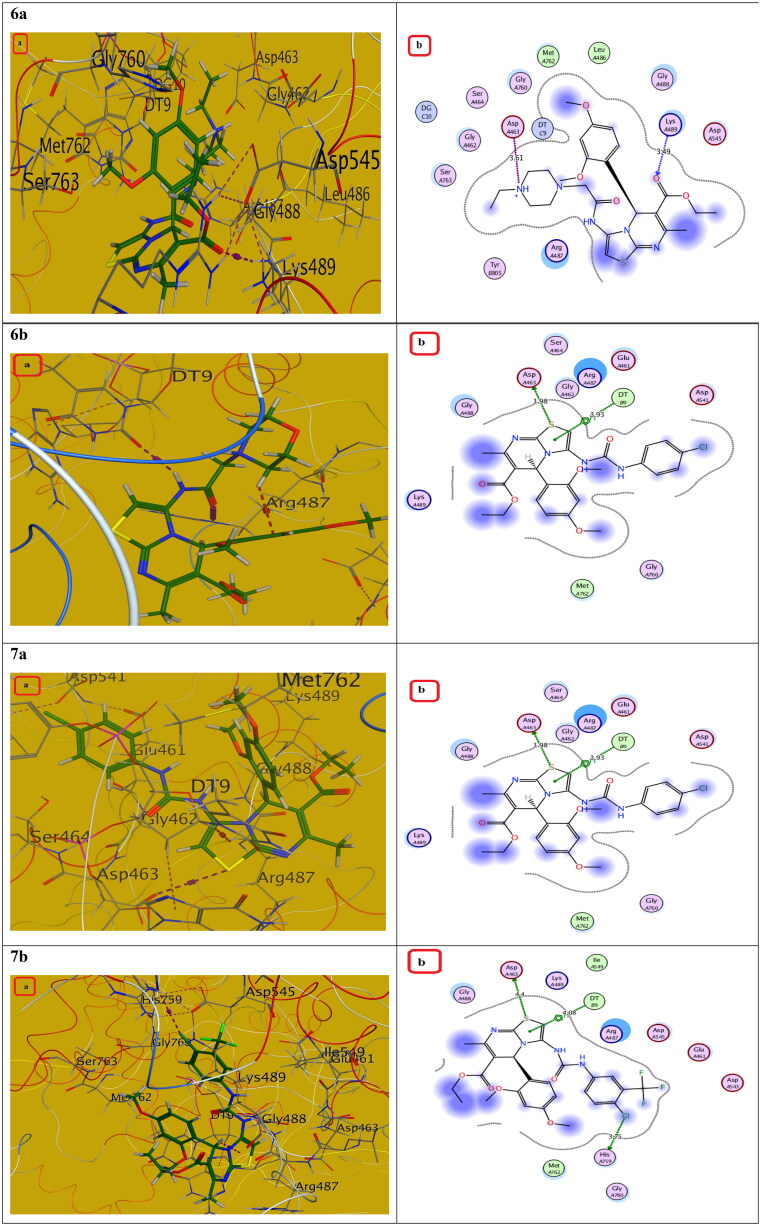
The (a) 3D and (b) 2D interaction mode of compounds **6a, 6b** and **7a, 7b** within topoisomerase II binding pocket. The pink dot arrow represents H-bonds and brown dot arrow represents H-π interaction.

Compounds with the highest cytotoxic activity (**3b-d, 4c, 6a, 6b, 7a,** and **7b)** were selected for the docking study to gain further insight into the binding modes of the synthesised compounds into the DNA binding site. MOE2014 was used for docking in order to determine the free energy and binding mode. Topoisomerase II structure was obtained from the protein data bank (PDB ID:5GWK) database. The docking validation was performed by docking Etoposide to the DNA binding site. Docking binding mode, energy score, and RMSD were presented in Table 5 and Figures 9–13. The results revealed that Etoposide was able to form hydrogen bonding interactions with Asp 463 and Met 766 amino acids in the topoisomerase II binding pocket with a binding energy score of −10.3 kcal/mol. Furthermore, the binding mode of the reference drug, Doxorubicin, with topoisomerase II binding site was also investigated, which was discovered to form four H-bonds with Asp 463, Asp 543, Ser 464, and Arg 617 amino acids, as well as a stacking interaction with Arg 487 and DT9 nucleotides, besides an ionic interaction with Asp 545 amino acid. Of all the tested thiazolopyrimidines, compound **4c** had the best binding interaction, forming an essential hydrogen bonding interaction with the same amino acid residues Asp 463 as Doxorubicin and Etoposide, besides π Stacking interaction with Arg 487 amino acid. Moreover, compounds **3b-d** with substituted benzylidene moiety at the C_2_ position of thiazolopyrimidin-3-ones revealed a typical π stacking interaction with Arg 487 amino acid as Doxorubicin. Compounds **3b** and **3c** formed additional π stacking interaction with DT-9, whereas compound **3d** formed hydrogen bonding with Gly 760 amino acid.

**Table 5.** Docking study outcomes of the selected compounds showing the binding interactions with topoisomerase II enzyme binding site.

Compound	Binding energy score ΔG (Kcal/mol)	RMSD (A^ο^)	Interactions bond length (Å)
Etoposide (Ligand)	–10.23	0.75	H. Bonds: Asp 463 (2.63 Å, 2.94 Å), Met 766 (3.88 Å)
Doxorubicin	–7.61	1.40	H. Bonds: Asp 463 (2.98 Å), Ser 464, (3.08 Å), Asp 543 (2.80 Å), Arg 617 (3.06 Å). π Stacking: Arg 487 (4.19 Å), DT9 (3.85 Å). Ionic interaction: Asp 545 (3.61 Å)
**3b**	–7.20	1.52	π Stacking: Arg 487 (3.90 Å), DT9 (4.22 Å)
**3c**	–7.30	1.47	π Stacking: Arg 487 (3.84 Å), DT9 (4.30 Å)
**3d**	–7.21	1.81	H. Bonds: Gly 760 (3.29 Å) π Stacking: Arg 487 (3.94 Å)
**4c**	–5.88	1.25	H. Bonds: Asp 463 (4.23 Å), His 459 (3.69 Å), Arg 713 (3.75 Å). π Stacking: Arg 487 (3.94 Å)
Metabolite of **4c**	–6.89	1.34	H. Bonds: Asp 463 (3.57 Å), Lys 489 (3.08 Å). π Stacking: Lys 489 (4.72 Å), Ionic interaction: Arg 713 (3.75 Å).
**6a**	–4.67	1.72	Ionic interaction: Asp 463 (3.61 Å). H. Bonds: Lys 489 (3.49 Å).
**6b**	–4.76	1.84	H. Bonds: Arg 487 (2.79 Å), DT9 (3.06 Å)
**7a**	–7.13	1.53	H. Bonds: Asp 463 (3.98 Å), π Stacking: DT9 (3.93 Å)
**7b**	–5.45	1.60	H. Bonds: Asp 463 (4.40 Å), His 759 (3.75 Å). π Stacking: DT9 (4.08 Å).

Additionally, the remaining thiazolopyrimidine derivatives with substituted acetamide and aminoamide side chains showed an interaction with the same amino acid Asp 463 as Etoposide and Doxorubicin, except for compound **6b,** which demonstrated hydrogen bonding interaction with DNA nucleotide DT9 and Arg 487 as Doxorubicin. We also considered the instability of the ester functional group, which will be easily metabolised inside the human body into an acidic group. Docking of the metabolite of compound **4c** (containing acid rather than ester functionality) was also performed and showed its ability to bind to topoisomerase II enzyme effectively *via* two H-bonds with (Asp 463 and Lys 489) amino acids as well as π Stacking with the same amino acid (Lys 489). Also, it showed an ionic interaction with Arg 713 amino acid. It should be noted that S-enantiomers were the active isomers that could bind to the topoisomerase II binding pocket, whereas R-isomers either had lower activity than S-isomers or showed no topoisomerase II inhibition activity. These findings were totally in agreement with the literature^25^. As a result, the designated compounds, particularly **4c**, may exhibit cytotoxicity by inhibiting the Topo II enzyme, resulting in the death of cancer cells in a mechanism similar to that of Etoposide and Doxorubicin.

#### 3.2.8. *In silico* physicochemical features, pharmacokinetics profiles, and drug-likeness data of most active compounds compared to doxorubicin and etoposide

To be a potential drug candidate, molecules must possess certain features, such as pharmacokinetics or pharmacodynamics, physicochemical properties, and drug-likeness. Therefore, the Swiss ADME online software (www.SwissADME.ch) was utilised to study the *in silico* ADME profile of the most active compounds **3c, 4c, 6a, 6b,** and **7b** in comparison with Doxorubicin (Dox) and Etoposide (ETP). Compounds **3c, 6a,** and **6b** are expected to be highly absorbed in the gastrointestinal tract (GIT), while compounds **4c** and **7b** demonstrated low GIT absorption like Doxorubicin and Etoposide, owing to their position outside the human intestinal absorption (HIA) region. Furthermore, the target compounds **3c, 4c, 6a, 6b,** and **7b** are characterised by a lack of BBB permeability, like Doxorubicin and Etoposide, suggesting that they may not penetrate the CNS. Moreover, compounds **3c, 6a,** and **6b** are thought to inhibit one or two of the five main cytochrome P-450 (CYP) isoforms in the liver, allowing them to be used safely alongside other medications. On the other hand, compounds **4c** and **7b** are anticipated to inhibit four cytochrome P-450 (CYP) isoforms in the liver; thus, they should be administered at time intervals when any other medications are prescribed to evade any possible drug-drug interactions. Doxorubicin did not affect any of the five major cytochrome P-450 (CYP) isoforms, whereas Etoposide inhibited only one of the CYP isoforms, CYP2D6 (Table 6).

**Table 6.** The *in silico* predicted pharmacokinetics for most active compounds compared to Doxorubicin and Etoposide.

Molecule	GI absorption	BBB permeable	CYP1A2 inhibitor	CYP2C19 inhibitor	CYP2C9 inhibitor	CYP2D6 inhibitor	CYP3A4 inhibitor
(3c)	High	No	No	No	Yes	No	Yes
(4c)	Low	No	Yes	Yes	Yes	No	Yes
(6a)	High	No	No	No	No	No	Yes
(6b)	High	No	No	No	Yes	No	Yes
(7b)	Low	No	Yes	Yes	Yes	No	Yes
DOX	Low	No	No	No	No	No	No
ETP	Low	No	No	No	No	Yes	No

The physicochemical parameters of the most active compounds **3c, 4c, 6a, 6b,** and **7b** are listed in Table 7. The target compounds have a molecular weight ≥500 Da, implying they will vary from easy to moderately diffuse and absorb into the cell membrane. Furthermore, compounds **3c, 6a,** and **6b** are expected to exhibit high membrane permeability since they meet the ideal log *p* values, while **4c** and **7b** do not. Additionally, they were shown to be ideal H-bond acceptors <10 (6–9) and H-bond donors <5 (0–2), in contrast to Doxorubicin and Etoposide, allowing the molecule to pass through the aqueous pores of biological membranes by passive diffusion. Besides, each of these compounds contains nine to ten rotatable bonds (≤10), implying good molecular flexibility. Moreover, all target compounds produced good TPSA, ranging from 119.39 to 131.86 Ǻ2 that outperformed the reference drugs. Finally, compound **3c** was found to be moderately soluble, while compounds **6a** and **6b** were proposed to be soluble as reference drugs, in contrast to compounds **4c** and **7b**, which were found to be poorly soluble. According to NRB and TPSA, the target compounds exhibit an interesting oral bioavailability. In general, the physicochemical properties of the target compounds **3c, 4c, 6a, 6b,** and **7b** were nearly identical to or better than those of Doxorubicin and Etoposide (Table 7).

**Table 7.** *In silico* physicochemical properties for most active compounds in comparison to doxorubicin and etoposide.

Molecule	^a^MW ≤500	^b^Log *P*_o/w_ ≤5	^c^HBA ≤10	^d^HBD ≤5	^e^TPSA Ǻ^2^ ≤140	^f^NRB ≤10	^g^Log S
(3c)	524.59	2.45	8	0	125.82	9	–5.6**
(4c)	582.88	5.55	6	1	119.39	9	–6.62***
(6a)	529.65	1.76	8	1	125.87	10	–3.97*
(6b)	502.58	1.23	8	1	131.86	10	–3.53*
(7b)	597.01	6.61	9	2	131.42	10	–6.15***
DOX	543.52	−0.32	12	6	206.07	5	–3.91*
ETP	588.56	1.01	13	3	160.83	5	–3.75*

^a^MW: molecular weight; ^b^Log *P*_o/w_: partition coefficient octanol/water; ^c^HBA: number of H-bond acceptors; ^d^HBD: number of H-bond donors; ^e^TPSA: topological polar surface area; ^f^NRB: number of rotatable bonds; ^g^Log S: Aqueous solubility (*soluble;**moderately soluble; ***poorly soluble).

The SwissADME Web tool demonstrated that compounds **3c, 4c, 6a, 6b,** and **7b** almost compiled with the drug-likeness rules proposed by Lipinski’s (Pfizer)^54^ and Veber’s (GSK)^55^ filters for pharmaceutical companies. Lipinski and Veber’s rules are two of the most prominent methods for identifying drug-like compounds and their oral bioavailability. Lipinski’s guidelines are focussed on finding compounds with permeability and absorption problems, whereas Veber’s rules are concerned with molecular flexibility and topological polar surface area. Except for MW, all target compounds almost completely followed Lipinski rules, while Veber’s guidelines were entirely followed by the under-investigated compounds. The examined compounds do not have PAINS (Pan Assay Interference Structures)^56^ or Brenks (Structural)^57^ alerts, indicating that there is no interference in any protein test, implying that the results of *in vitro* bioassays should be robust. Doxorubicin and Etoposide were found to violate both Lipinski’s (Pfizer) and Veber’s (GSK) drug-likeness rules (Table 8).

**Table 8.** The drug-likeness of most active compounds as well as the references Doxorubicin and Etoposide drugs.

Molecule	Lipinski #violations	Veber #violations	PAINS #alerts	Brenk #alerts
(3c)	1	0	0	0
(4c)	1	0	0	0
(6a)	1	0	0	0
(6b)	1	0	0	0
(7b)	1	0	0	0
DOX	3	1	1	1
ETP	2	1	0	0

## 4. Conclusion

In summary, two series of 3-oxo-3,5-dihydrothiazolo[3,2-*a*]pyrimidines **3a-d** (Scaffold 1) and thiazolo[3,2-*a*]pyrimidine analogs **2**, **4a-c, 6a-c,** and **7a,b** (scaffold 2) have been prepared and screened against breast MCF7, lung A549, and renal A498 cancer cell lines. Several compounds, such as **3c, 3d, 4c, 6a, 6b,** and **7b**, exhibited potent to strong anticancer activity that was nearly comparable to and better than Doxorubicin. Among them, compound **4c** was the most potent, with IC_50_ values of 2 ± 0.05, 2 ± 0.07, and 1 ± 0.05 nM against MCF7, A549, and A498 cancer cell lines, respectively that was more active from 4.5-fold to 7-fold than Doxorubicin, which had IC_50_ values of 9 ± 0.26, 13 ± 0.42, and 7 ± 0.23 nM. The most promising cytotoxic agents have been investigated for Topo II enzyme inhibition activity. Compound **4c** was the most potent Topo II inhibitor, with an IC_50_ of 0.23 ± 0.01 µM, which was 1.4 and 3.6 times higher than the IC_50_ values of Etoposide (0.32 ± 0.02 µM) and Doxorubicin (0.83 ± 0.05 µM), respectively. While compounds **7b, 3c, 6b,** and **6a** showed marked Topo II inhibitory activity with almost high Doxorubicin potency. Furthermore, compound **4c** was examined for safety against the non-tumorigenic human WI-38 cell line, demonstrating low cellular cytotoxicity. Moreover, cell cycle analysis of compound **4c** on A549 cells demonstrated a dramatic fall in the cell population at the G2/M phase, resulting in cell proliferation inhibition. Additionally, its flow cytometry analysis revealed a significant increase in early and late apoptotic cells by 61.92-fold and 97-fold, respectively, compared with control cells. Finally, a molecular modelling study of the active compounds confirmed compound **4c’**s ability to bind with the same amino acid residues Asp 463 and Arg 487 as Doxorubicin in topoisomerase II enzyme binding site and embed in DNA grooves.

## Supplementary Material

Supplemental Material
