# Supplementary material for: Design, synthesis, docking, and anticancer evaluations of new thiazolo[3,2-a] pyrimidines as topoisomerase II inhibitors
Source: J Enzyme Inhib Med Chem. 2023 Feb 12;38(1):2175209. doi: 10.1080/14756366.2023.2175209 (PMC9930781; doi:10.1080/14756366.2023.2175209)
Supplement: Supplemental Material [file IENZ_A_2175209_SM8368.pdf]

Supplementary material

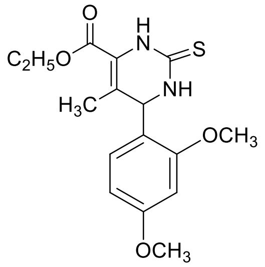

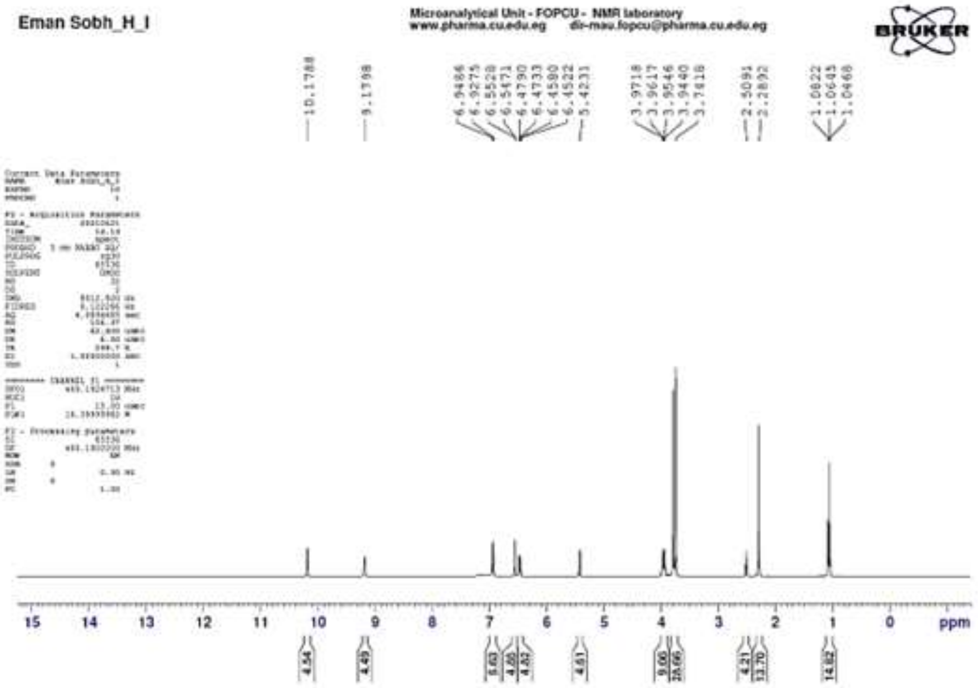

Figure S1. ^{1}HNMR of compound 1

Information Classification: General

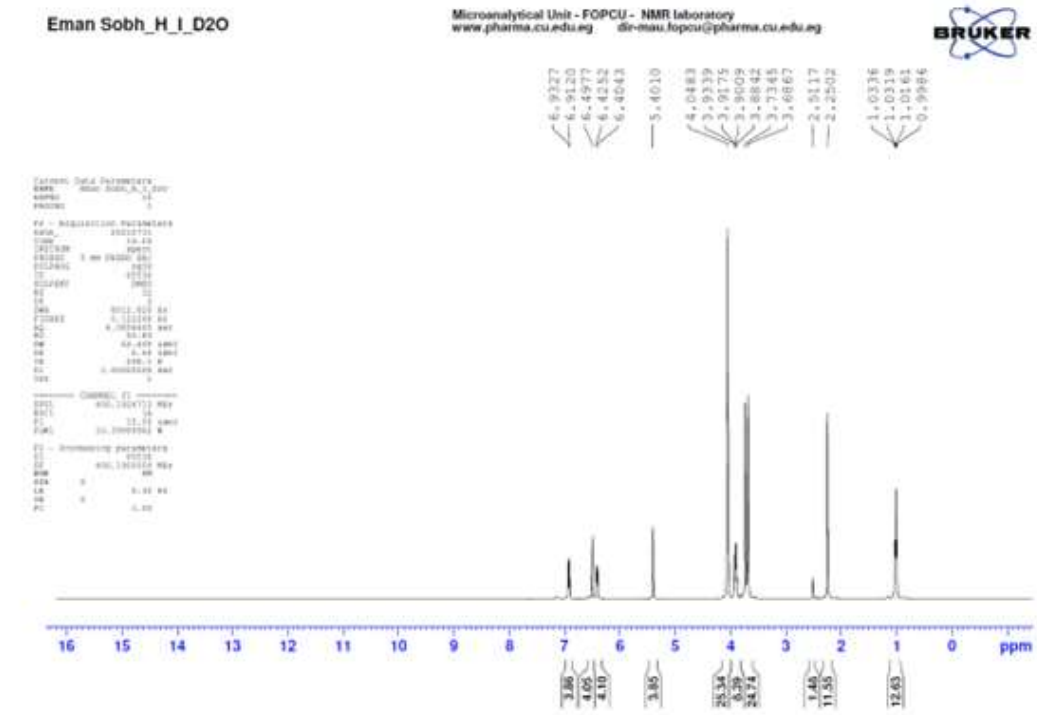

Figure S2. D_2O **of** ^{1}**HNMR of compound 1**

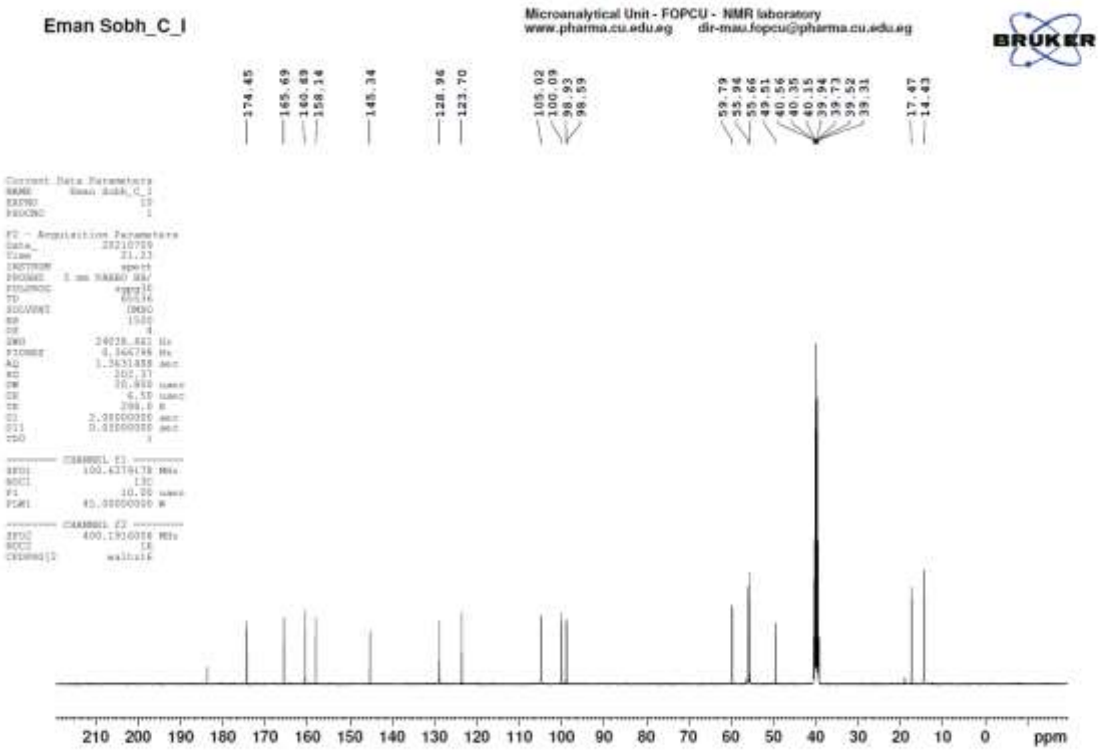

Figure S3. ^{13}**CNMR of compound 1**

Information Classification: General

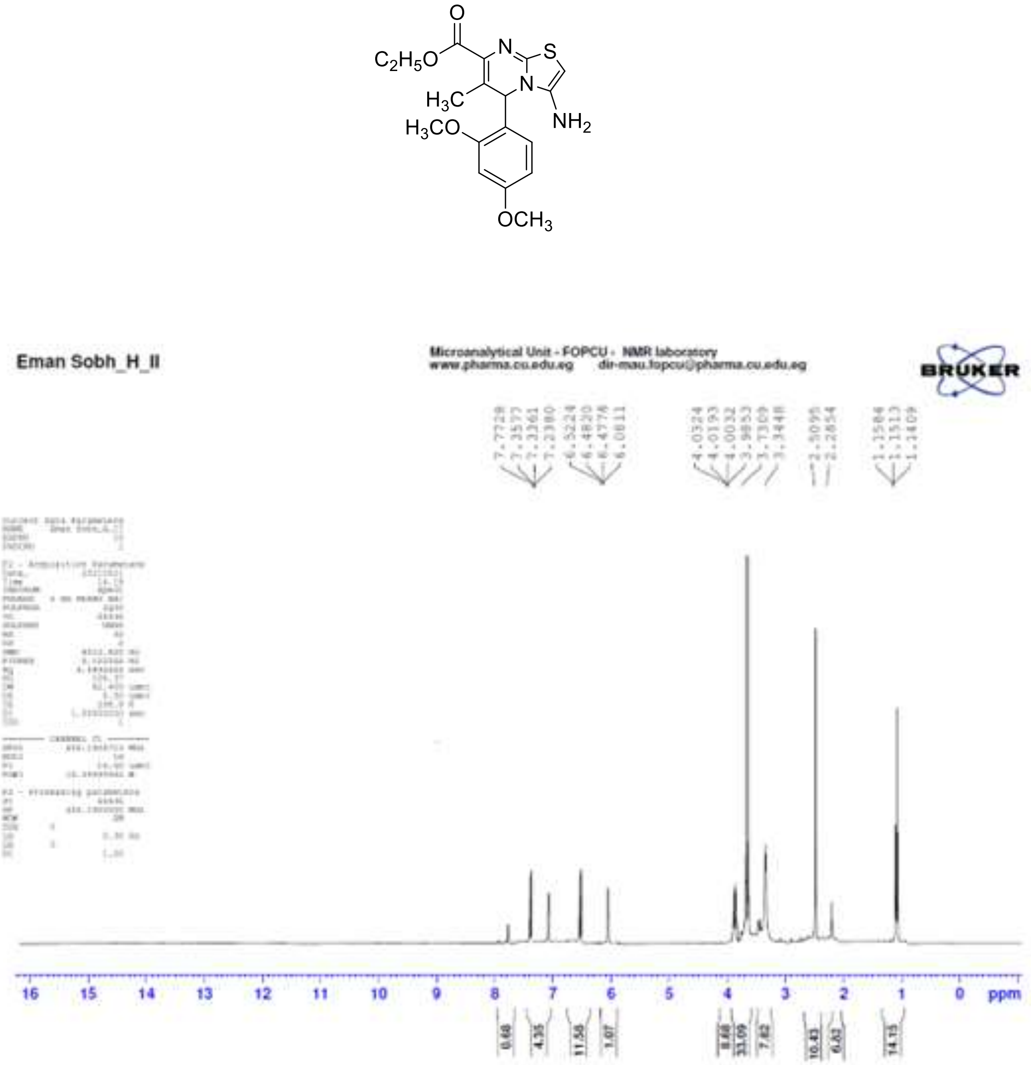

Figure S4. ^{1}HNMR of compound 2

Information Classification: General

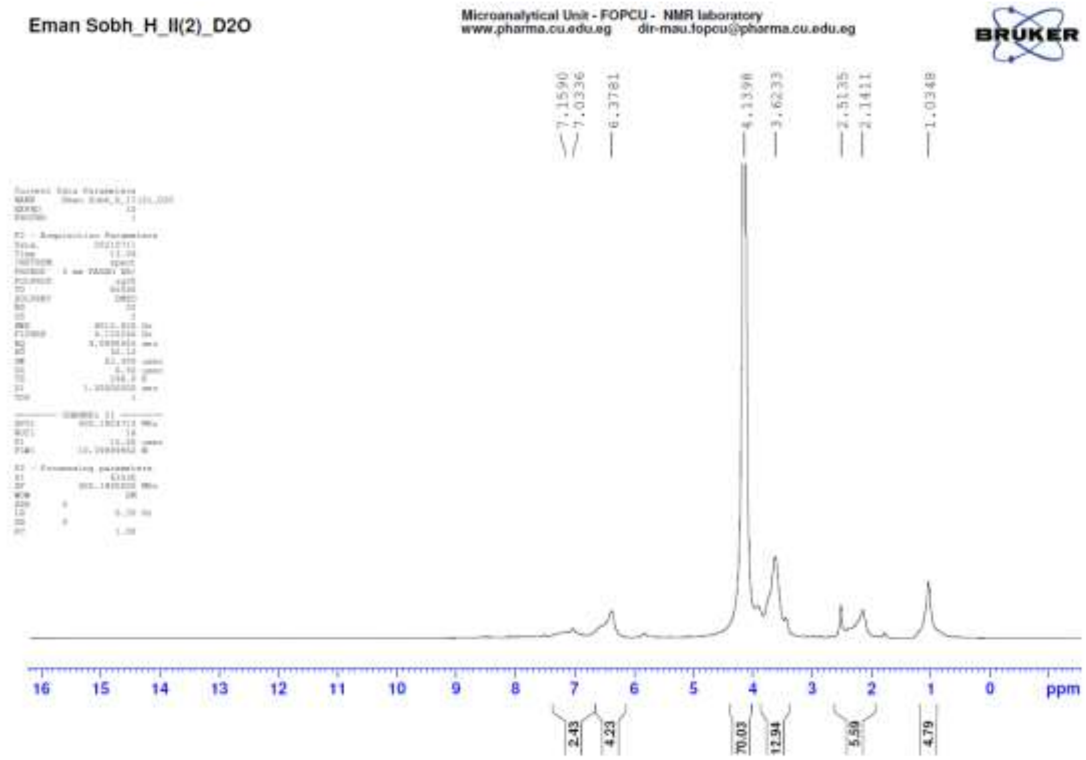

Figure S5. D2O OF ^{1}HNMR of compound 2

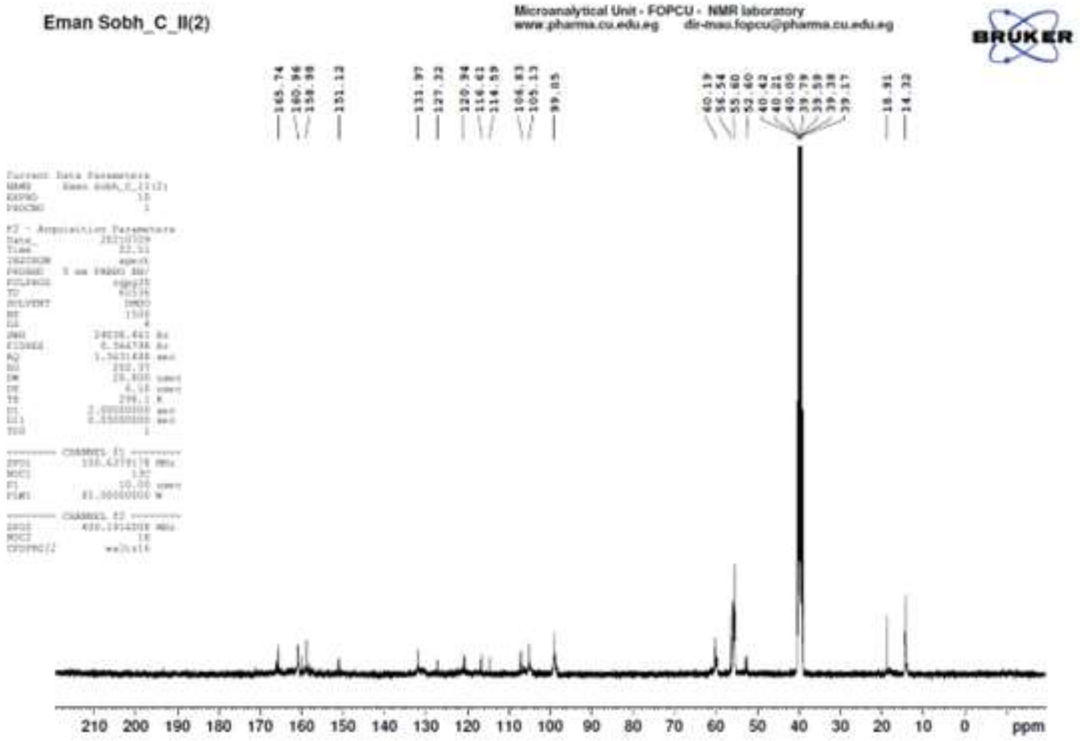

Information Classification: General

Figure S6. ^{13}CNMR of compound 2

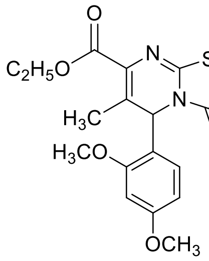

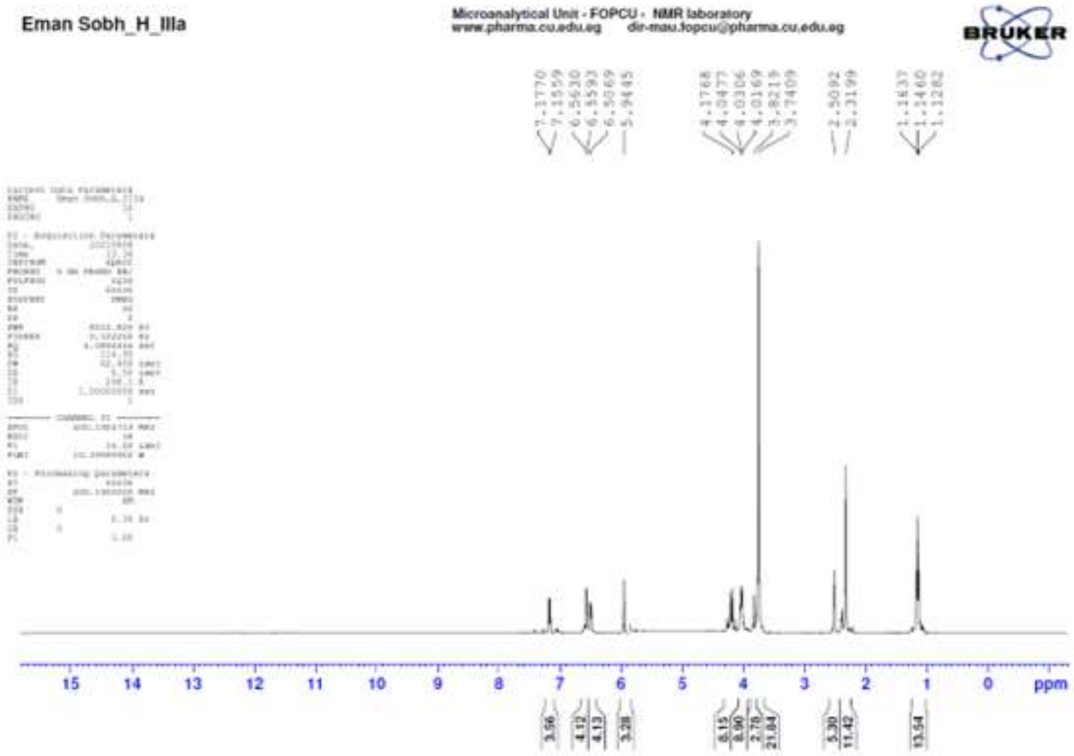

Figure S7. ^{1}HNMR of compound 3a

Information Classification: General

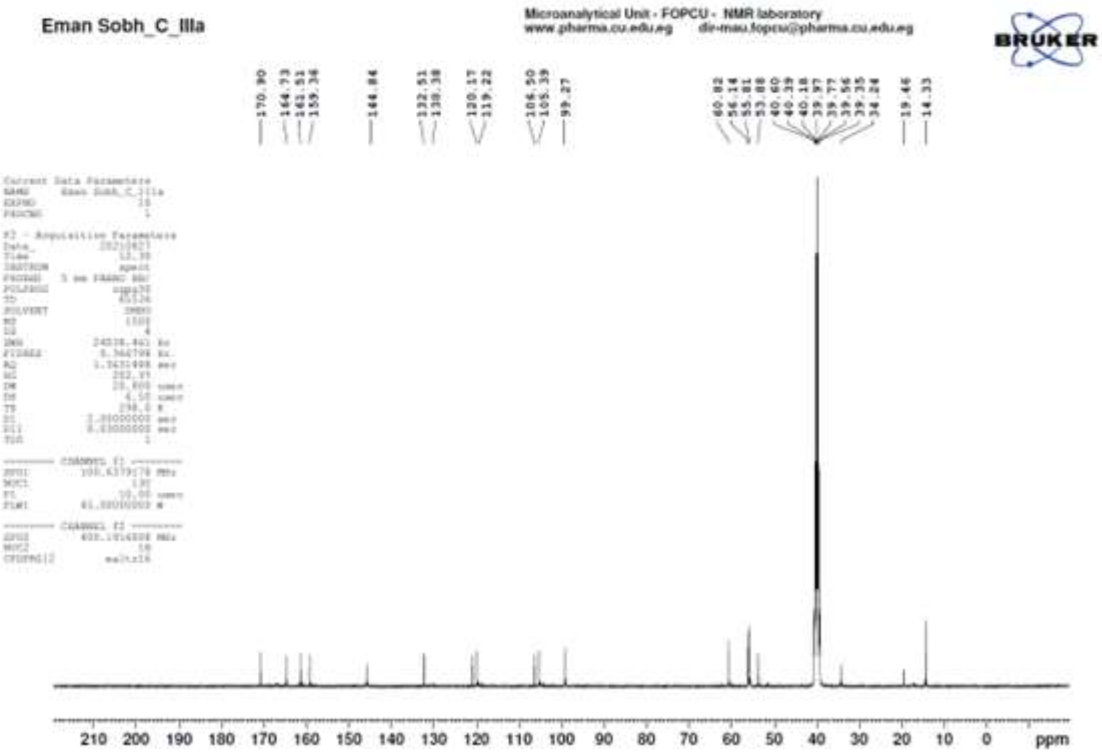

Figure S8. ^{13}**CNMR of compound 3a**

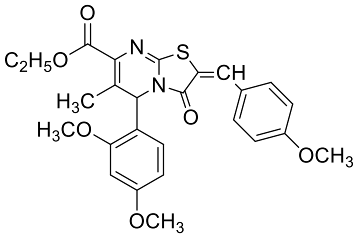

Information Classification: General

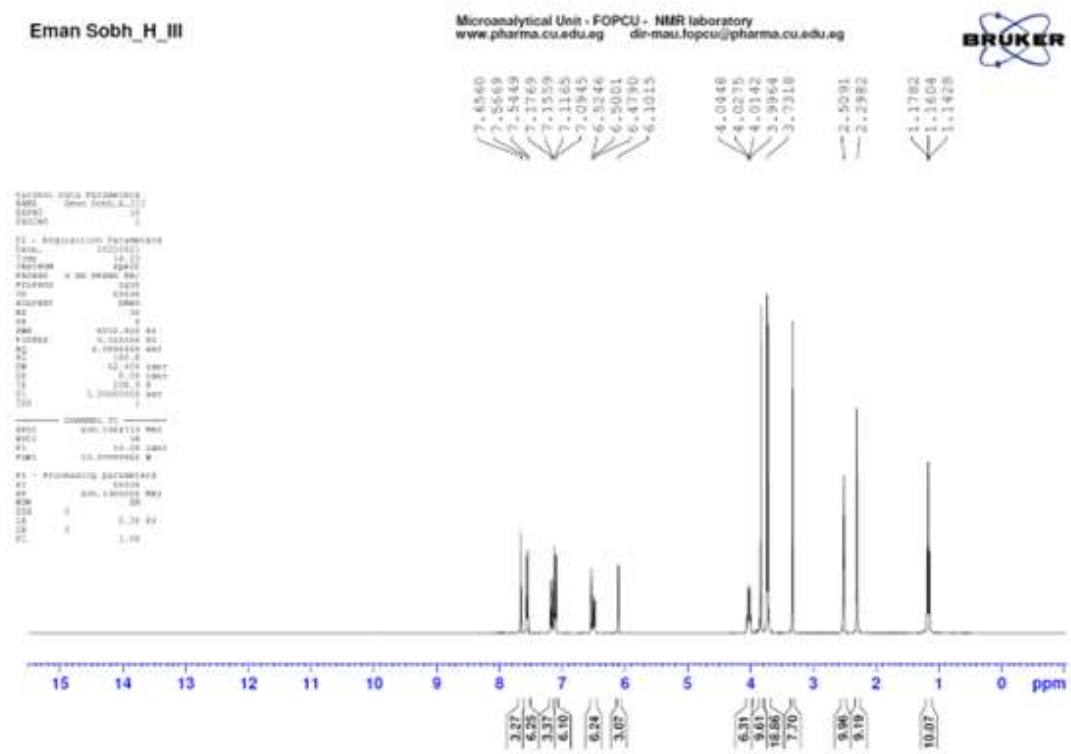

Figure S9. ^{1}HNMR of compound 3b

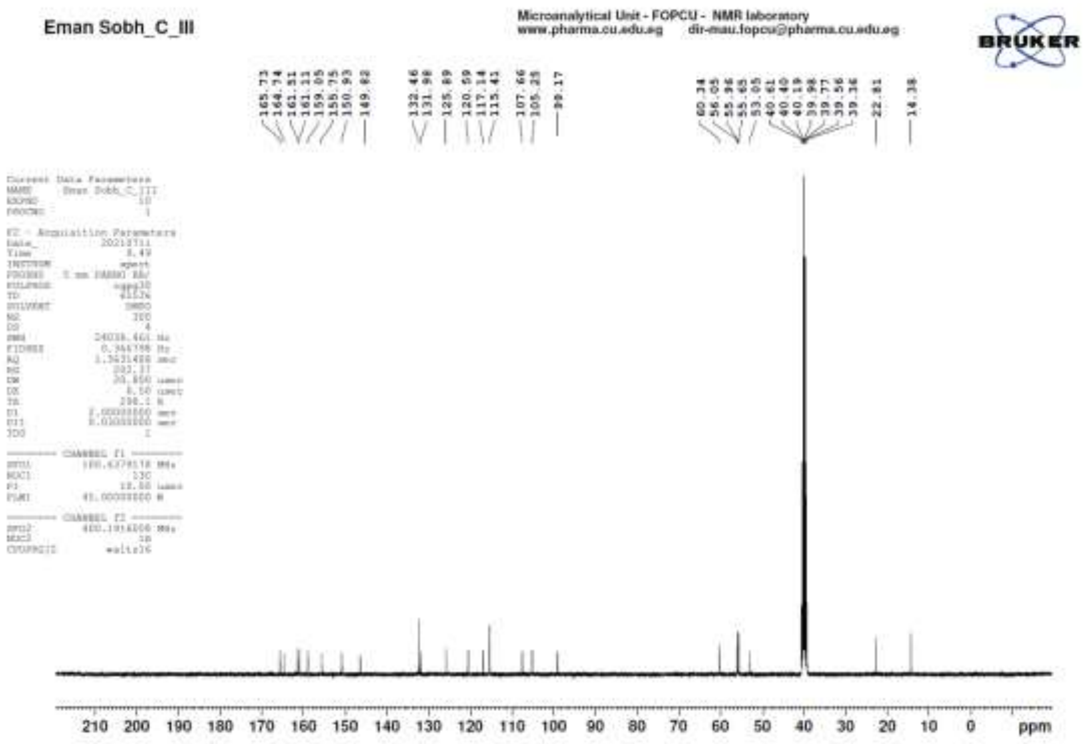

Figure S10. ^{13}CNMR of compound 3b

Information Classification: General

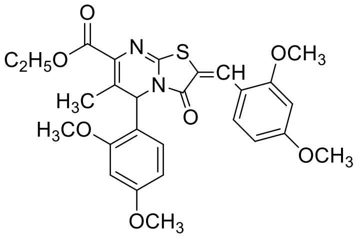

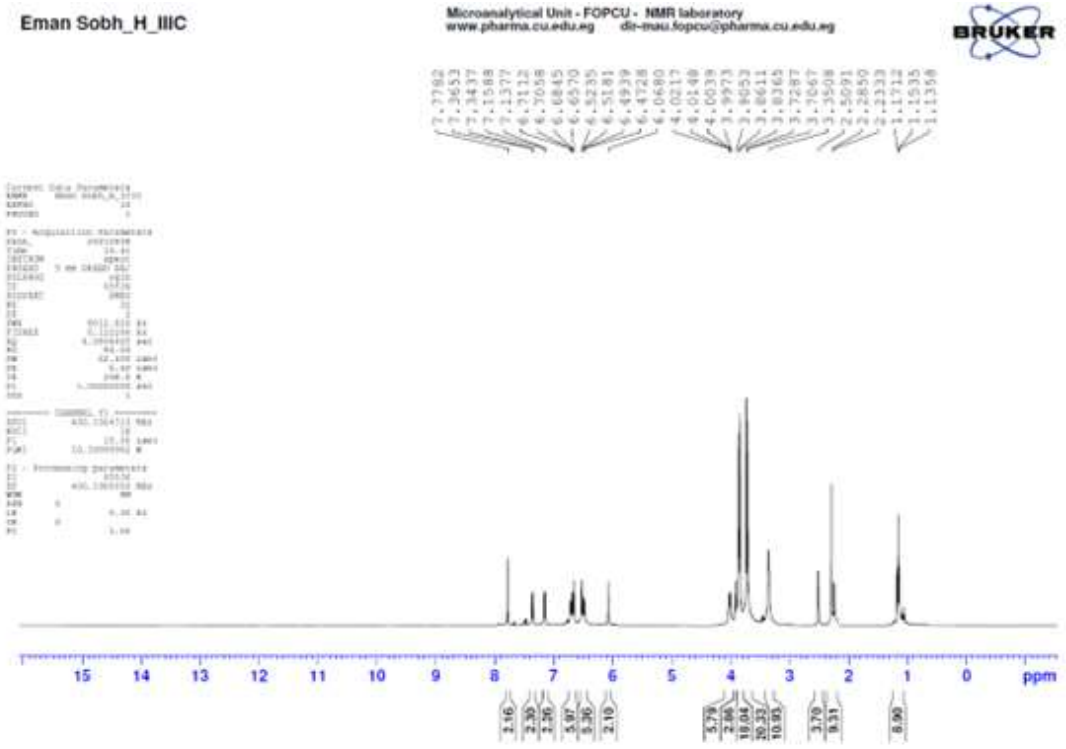

Figure S11. [1]**HNMR of compound 3c**

Information Classification: General

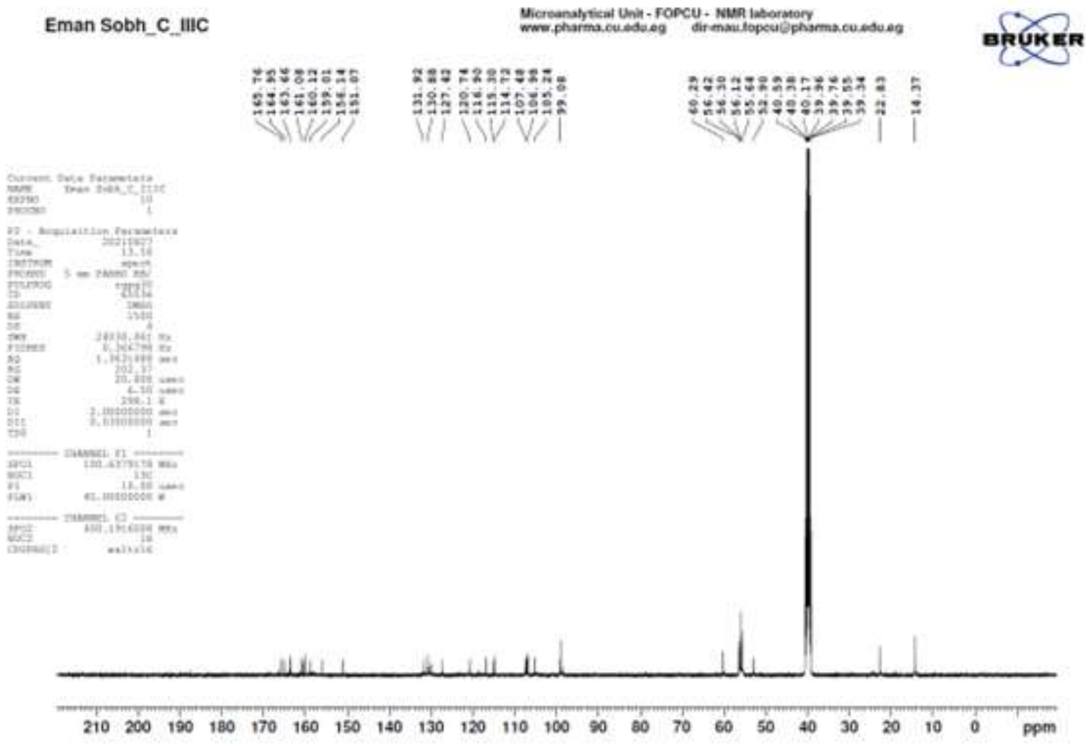

Figure S12. ^{13}**CNMR of compound 3c**

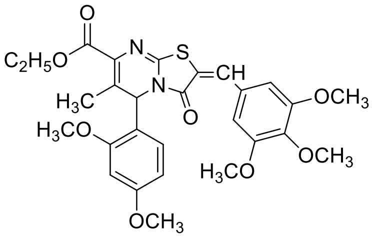

Information Classification: General

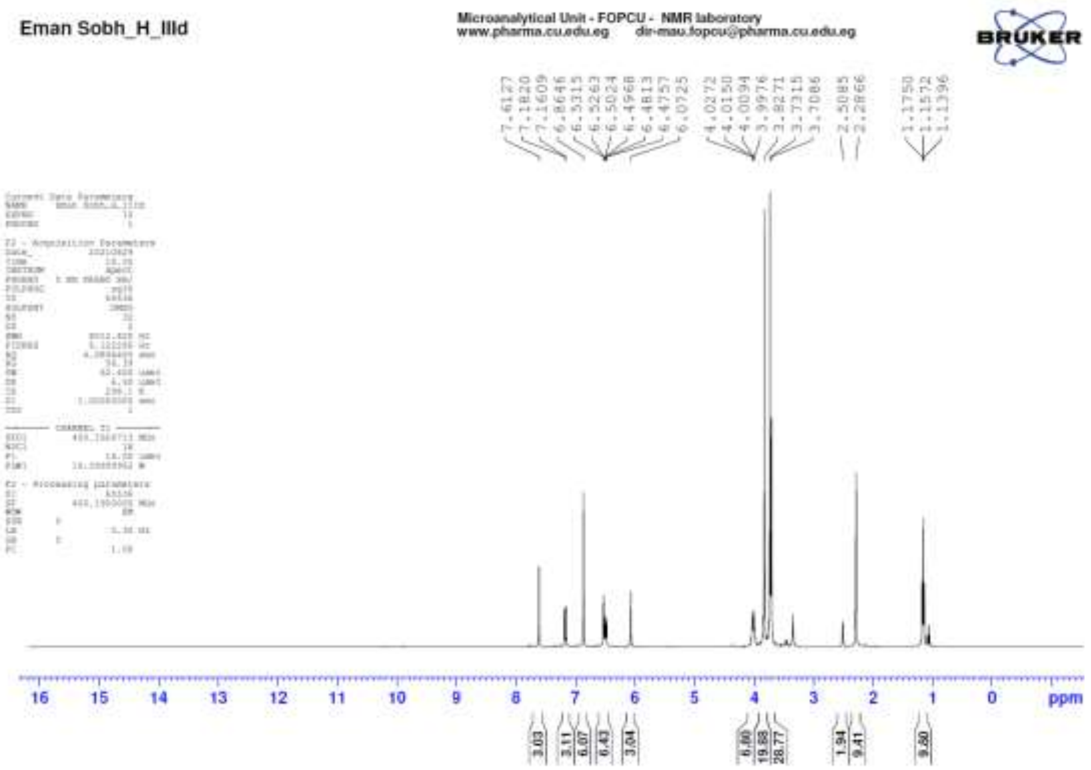

Figure S13. ^{1}HNMR of compound 3d

Information Classification: General

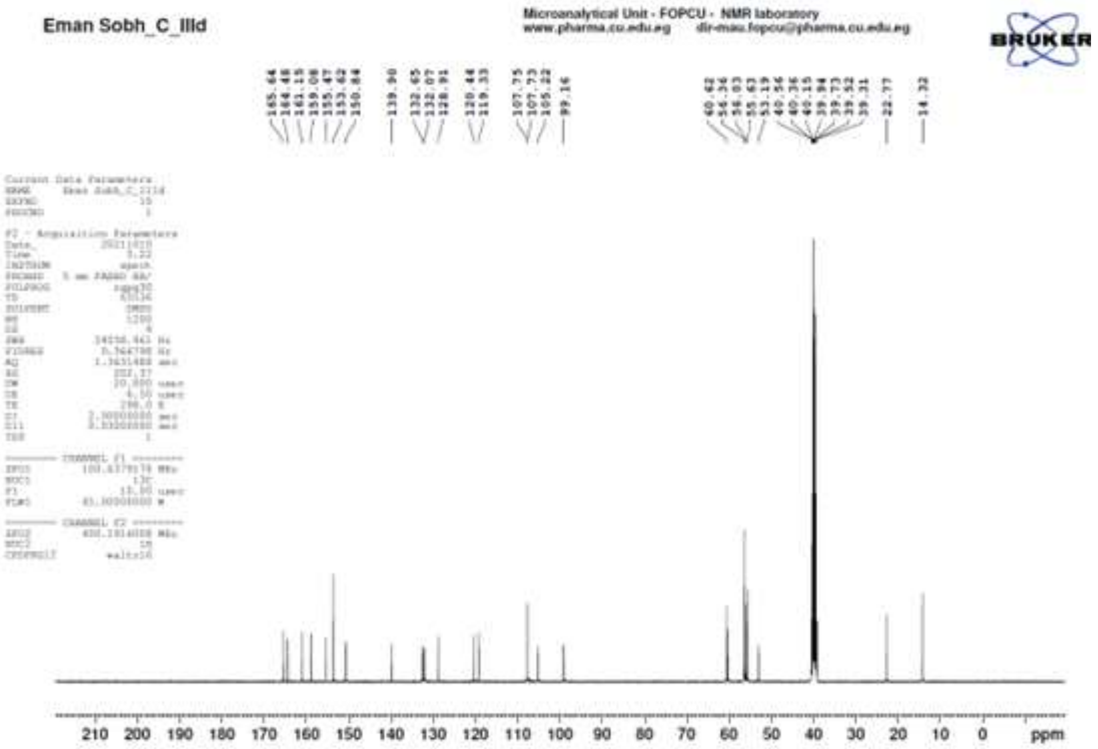

Figure S14. ^{13}CNMR of compound 3d

Figure S15. ^{1}HNMR compound 4a

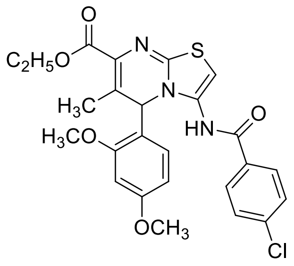

Information Classification: General

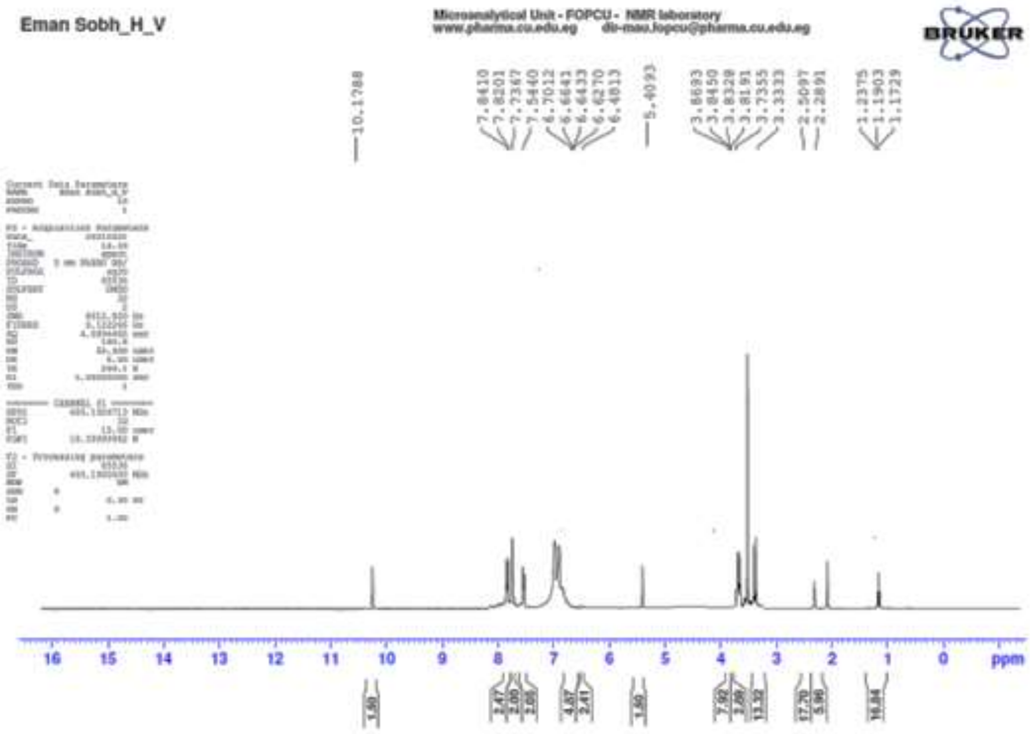

Figure S15. ^{1}HNMR of compound 4a

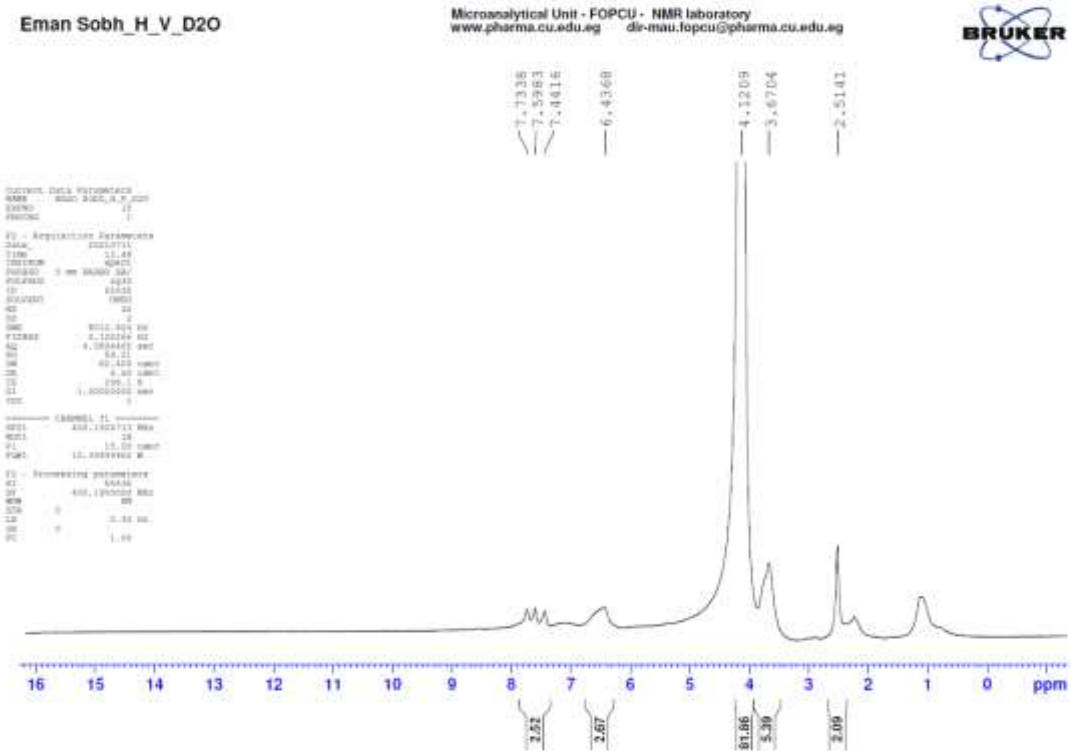

Figure S16. D_2O of ^{1}HNMR of compound 4a

Information Classification: General

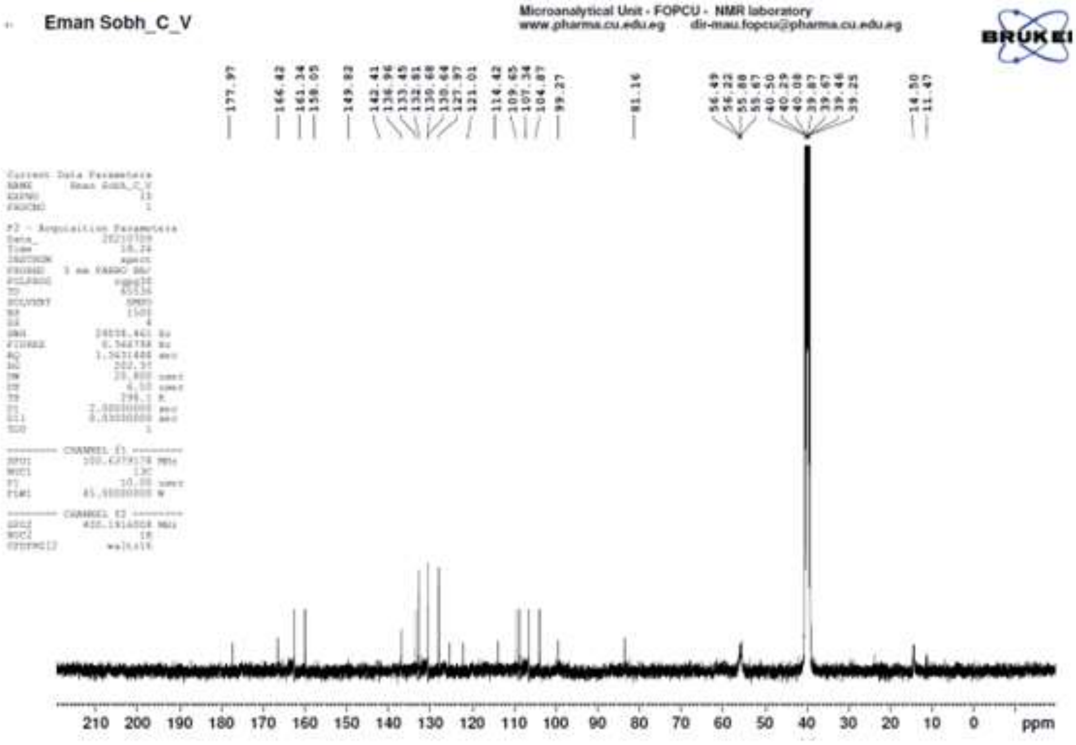

Figure S17. ^{13}CNMR of compound 4a

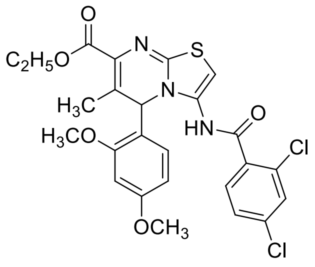

Information Classification: General

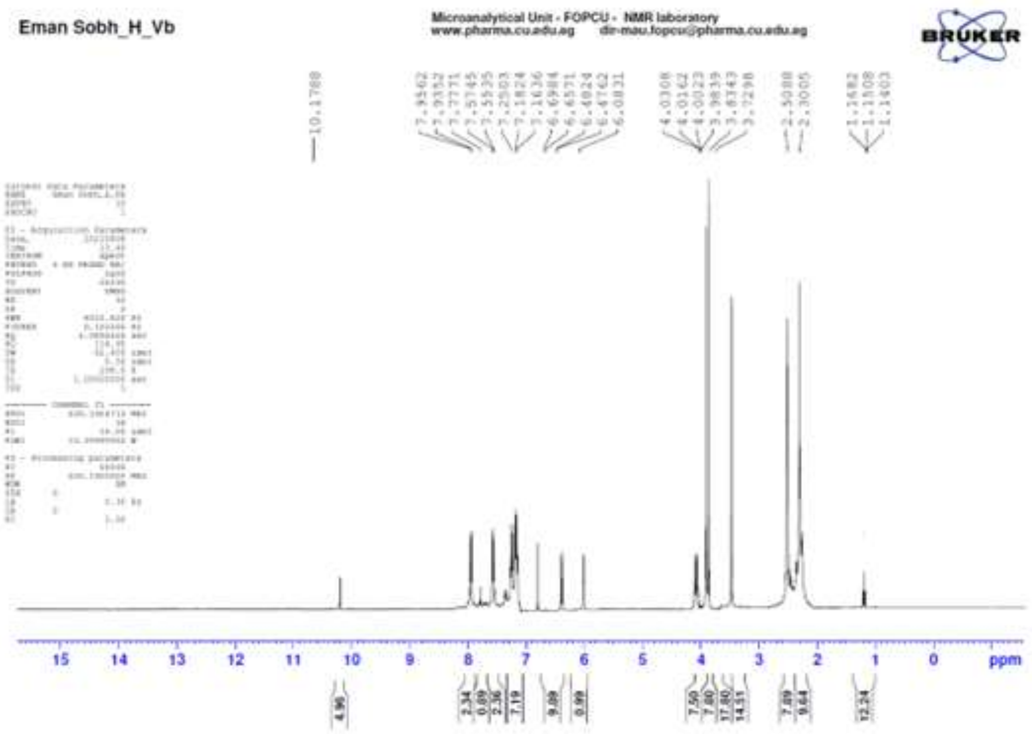

Figure S18. ^{1}HNMR of compound 4b

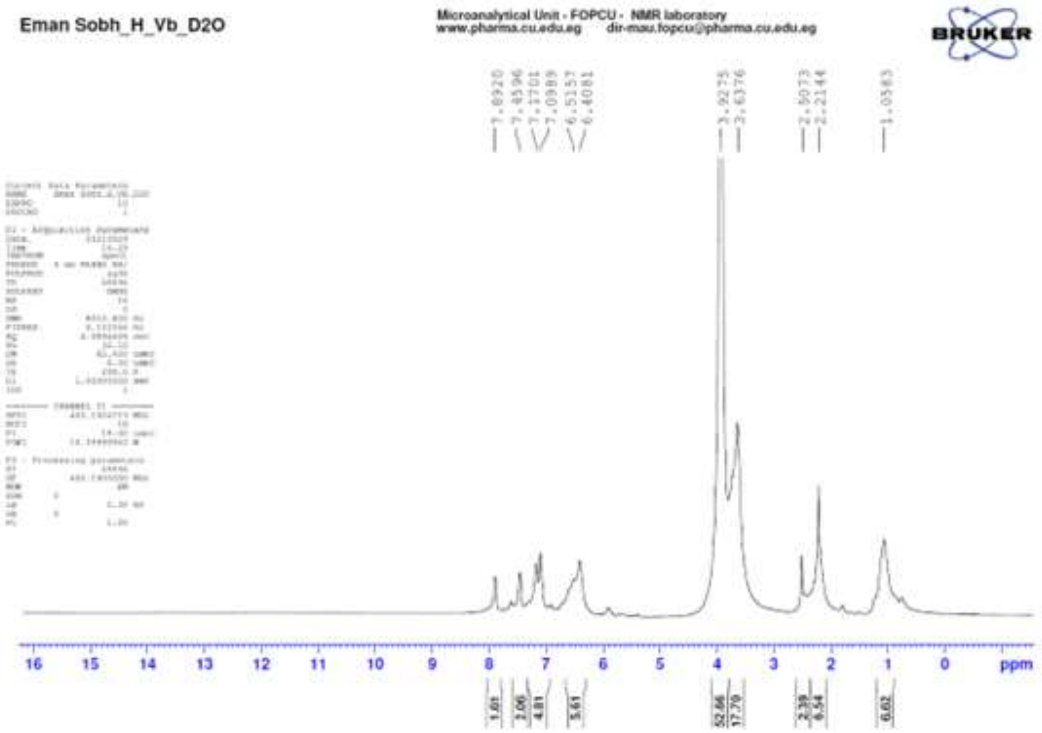

Figure S18. D_2O of ^{1}HNMR of compound 4b

Information Classification: General

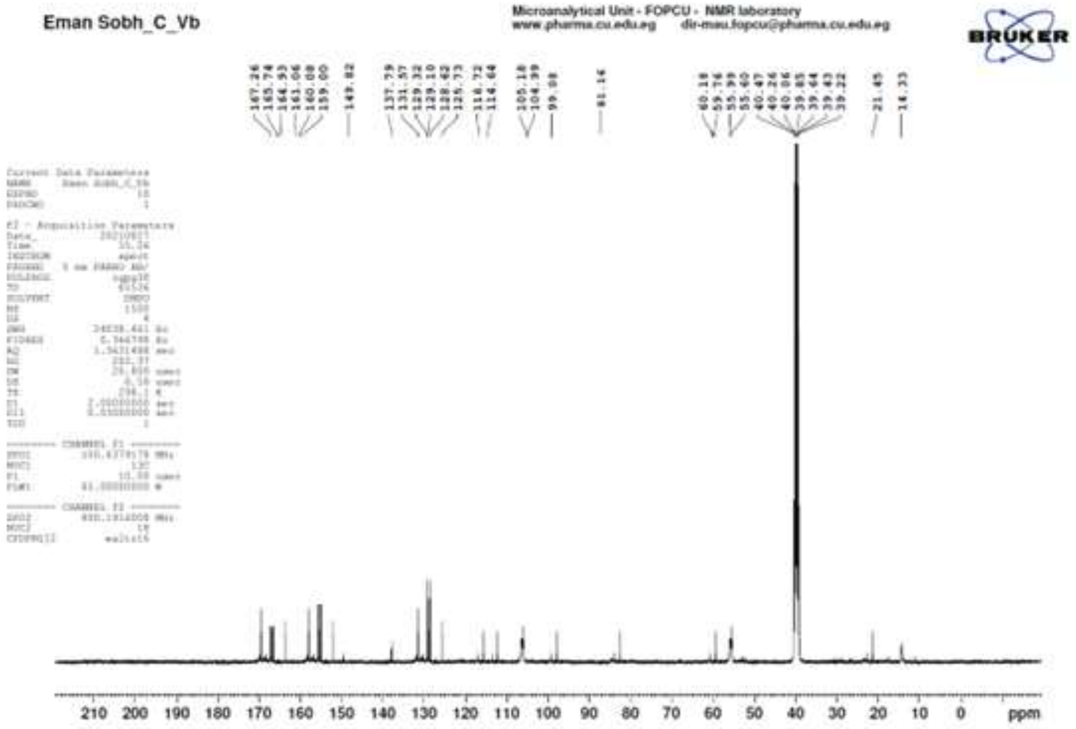

Figure S20. ^{13}CNMR of compound 4b

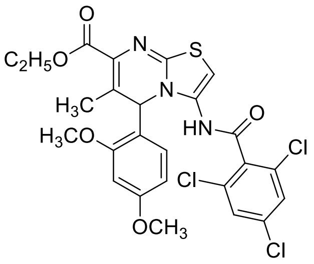

Information Classification: General

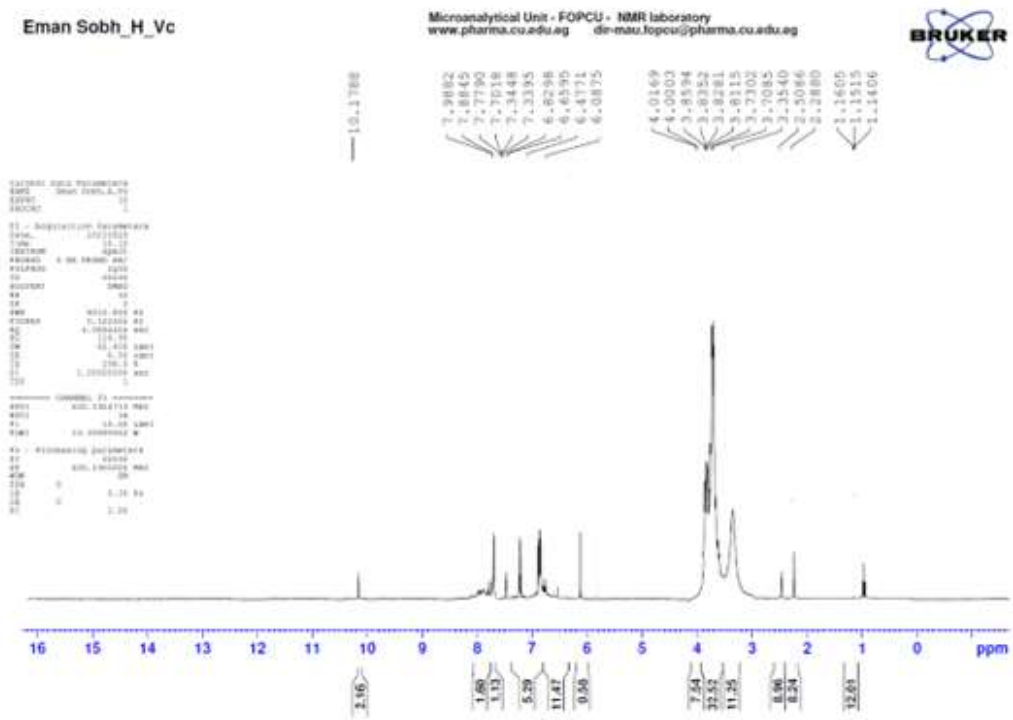

Figure S21. ^{1}HNMR of compound 4c

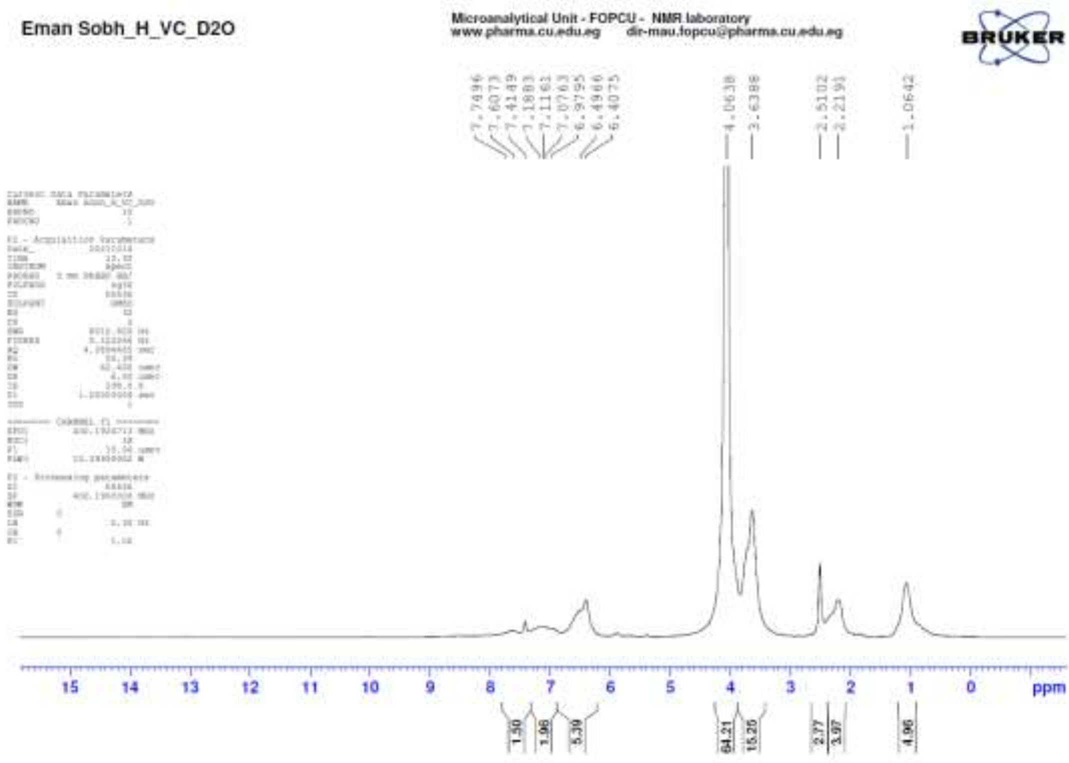

Figure S22. D2O of ^{1}HNMR of compound 4c

Information Classification: General

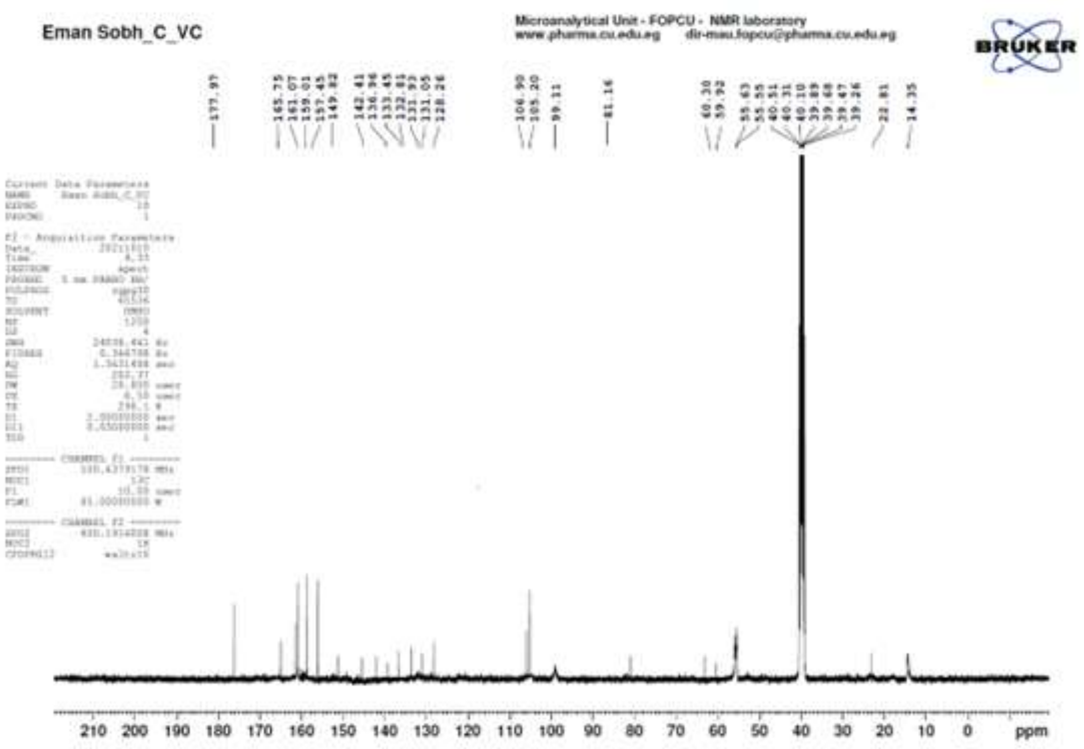

Figure S23. ^{13}**CNMR of compound 4c**

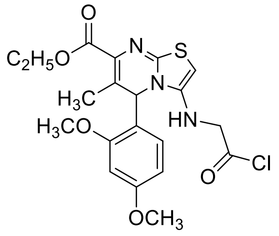

Information Classification: General

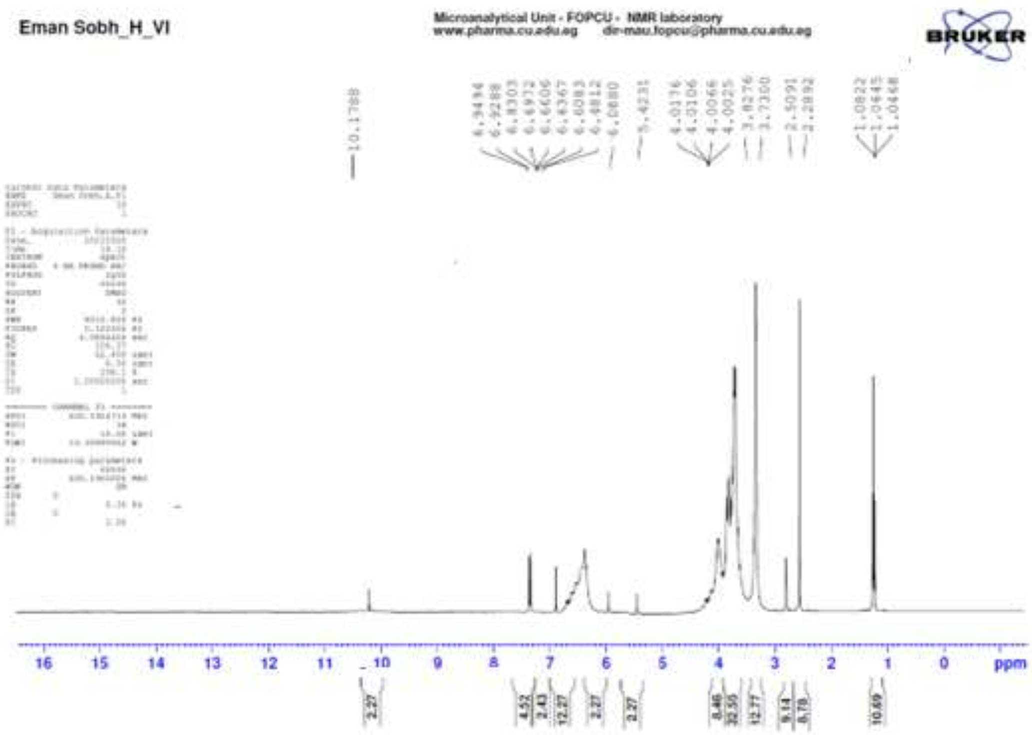

Figure S24. ¹HNMR of compound 5

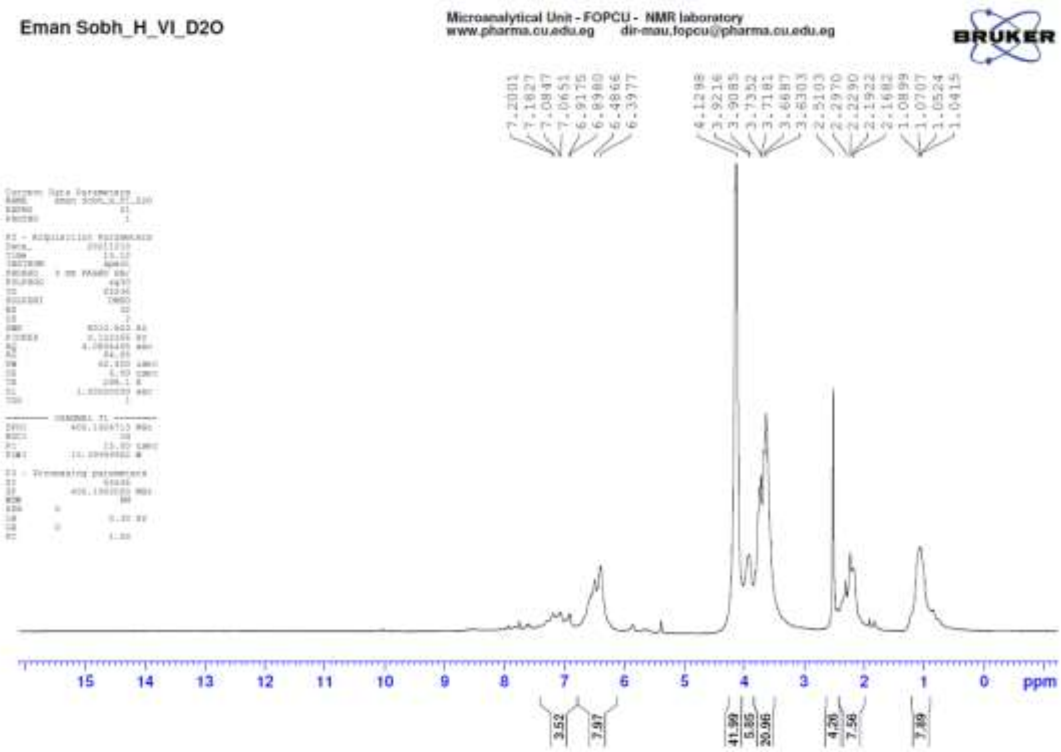

Figure S25. ¹HNMR (D_2O) of compound 5

Information Classification: General

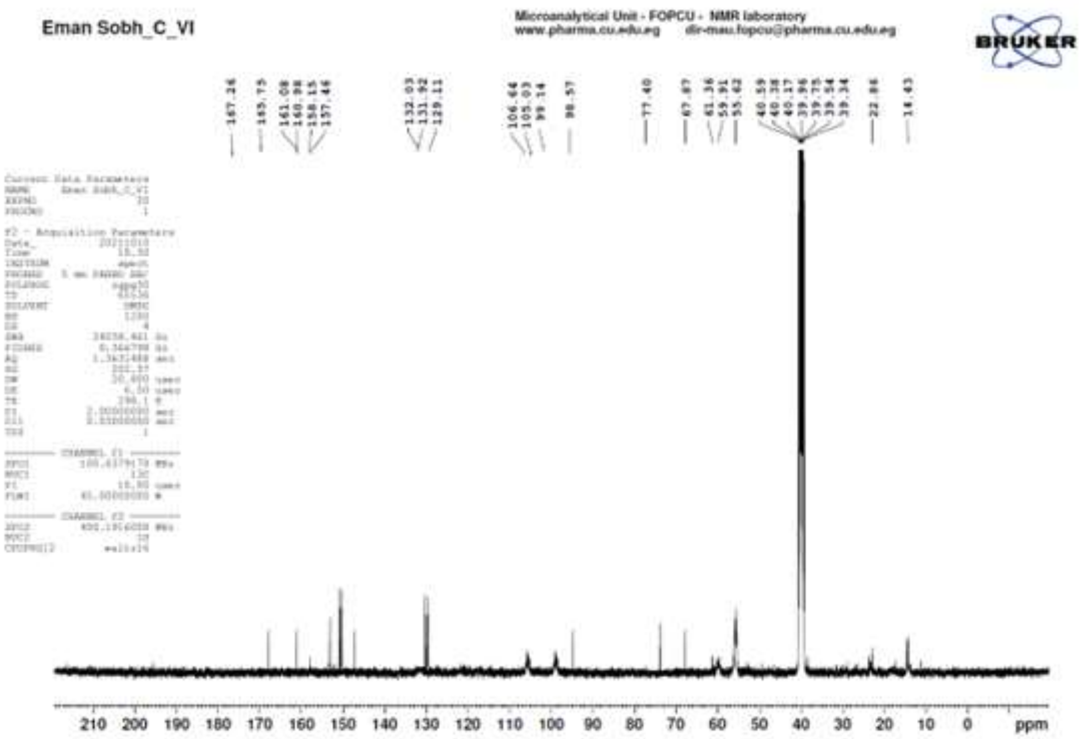

Figure S26. ^{13}CNMR of compound 5

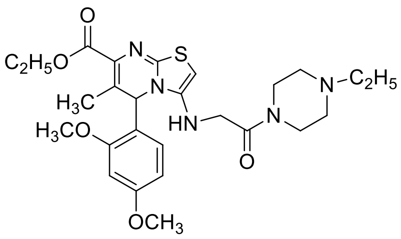

Information Classification: General

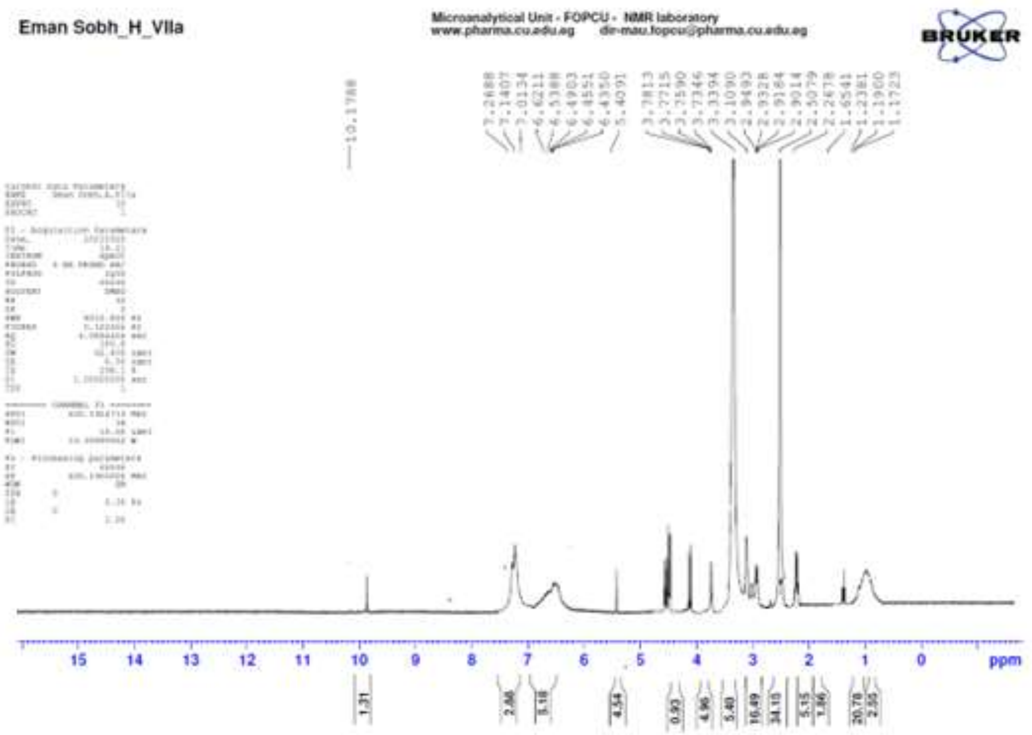

Figure S27. ^{1}HNMR of compound 6a

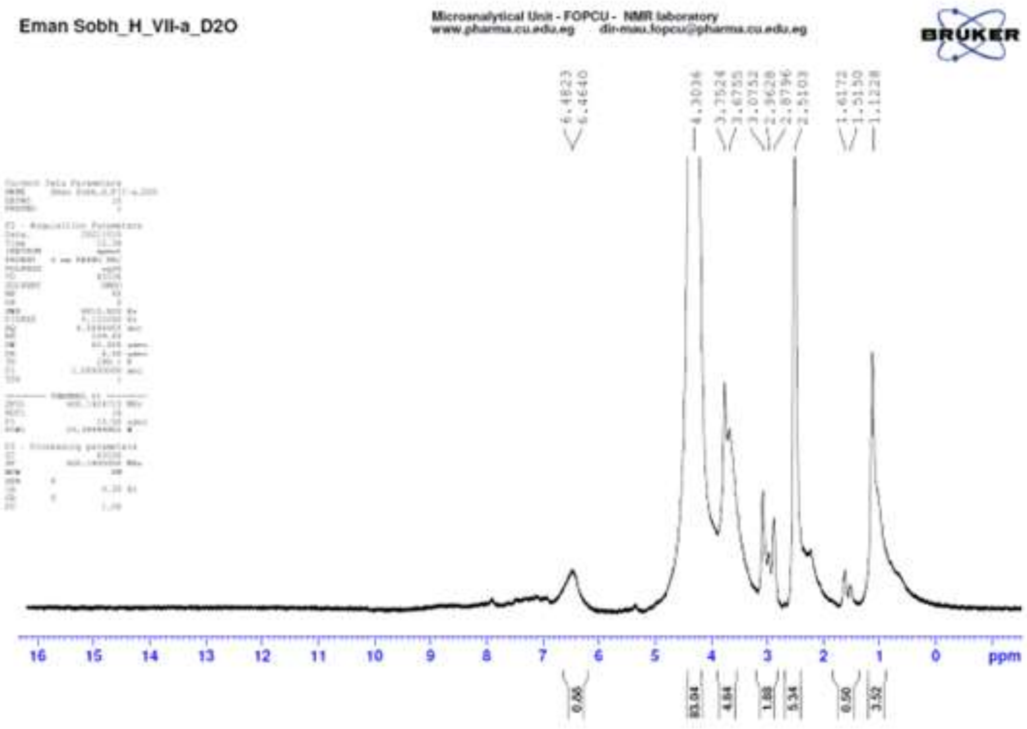

Figure S28. ^{1}HNMR (D_2O) of compound 6a

Information Classification: General

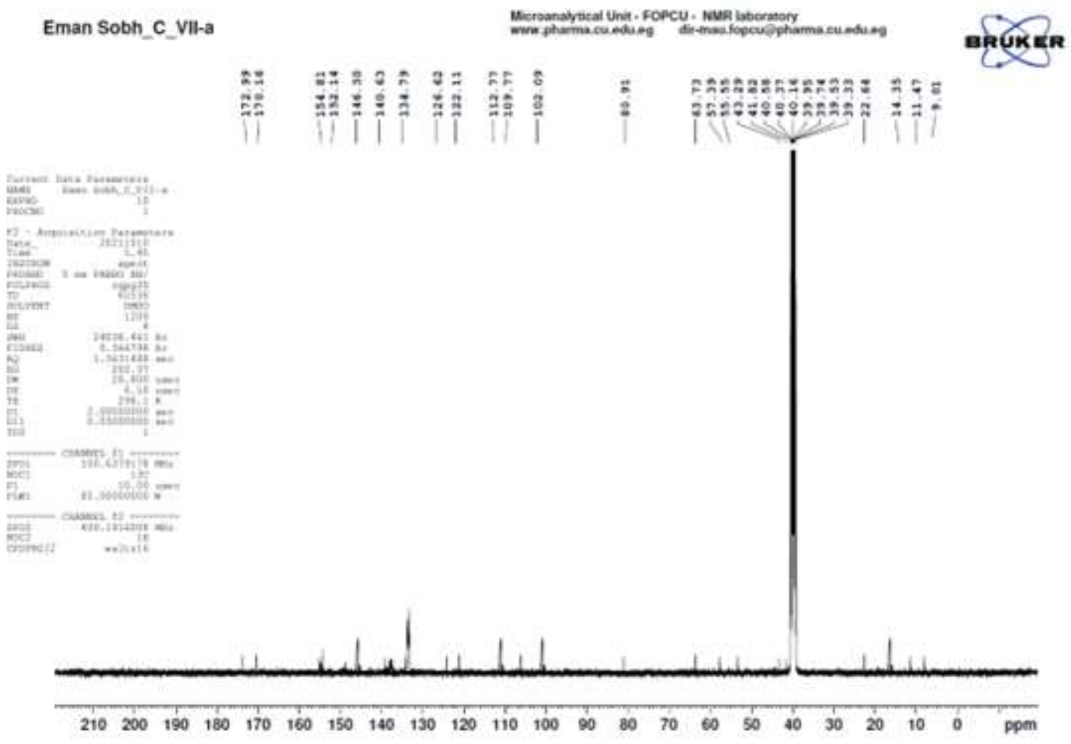

Figure S29. ^{13}**CNMR of compound 6a**

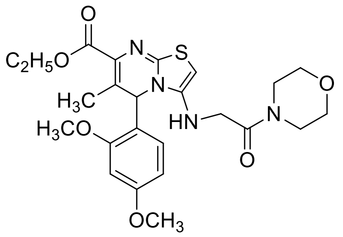

Information Classification: General

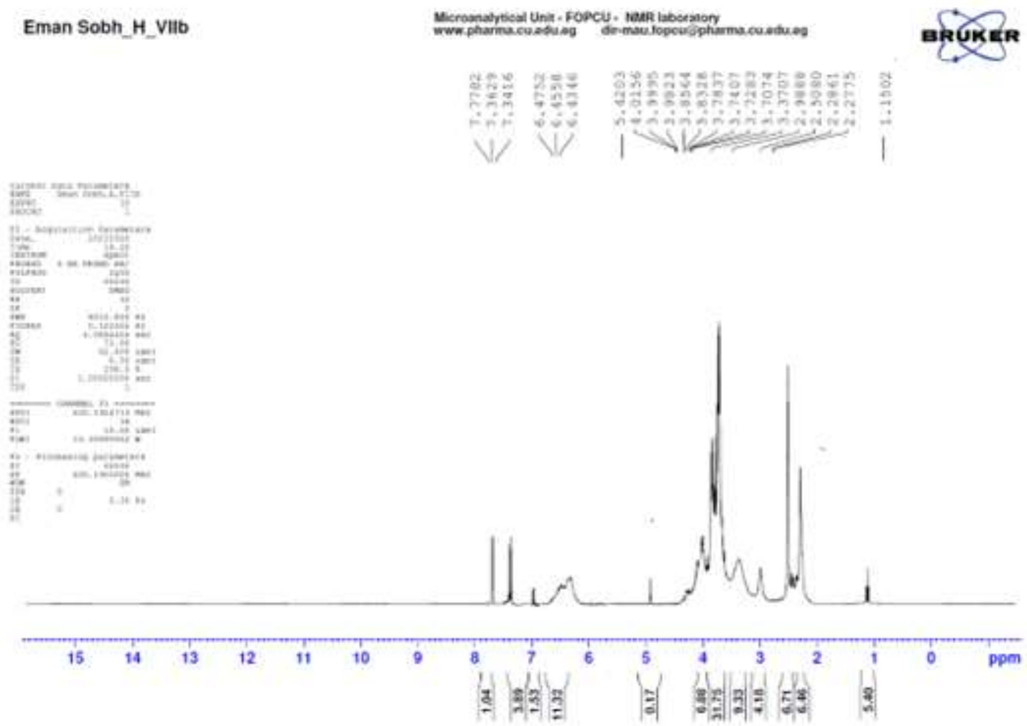

Figure S30. [1]**HNMR of compound 6b**

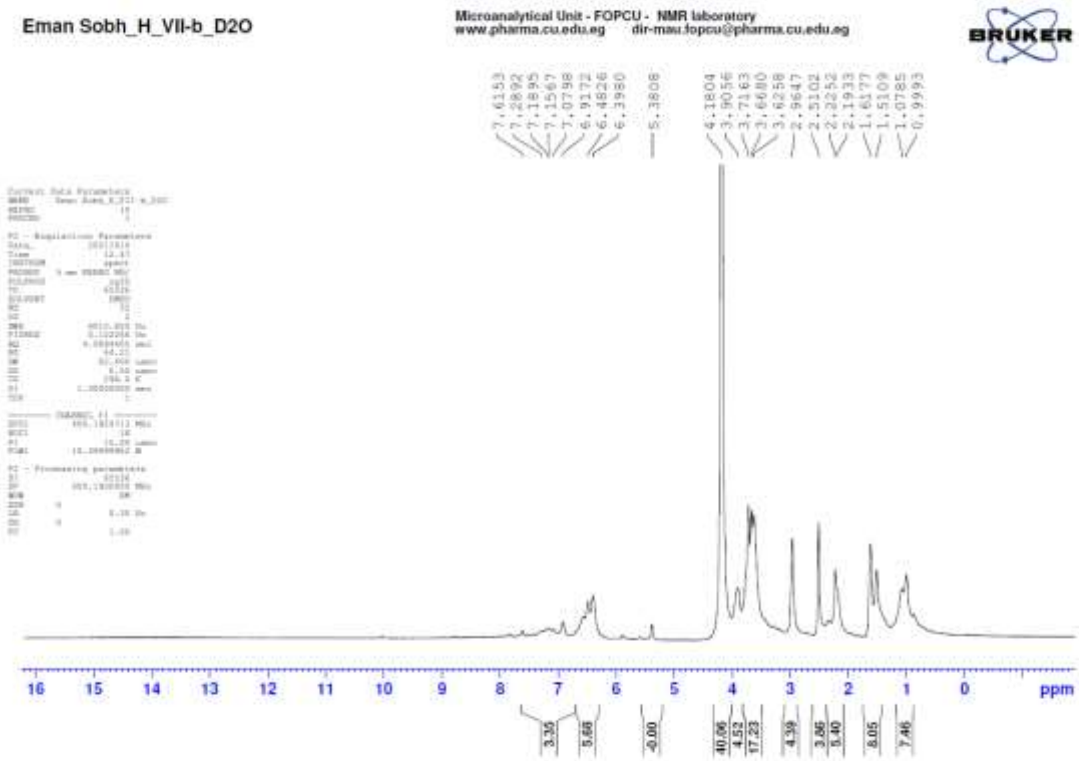

Figure S31. [1]**HNMR (D2O) of compound 6b**

Information Classification: General

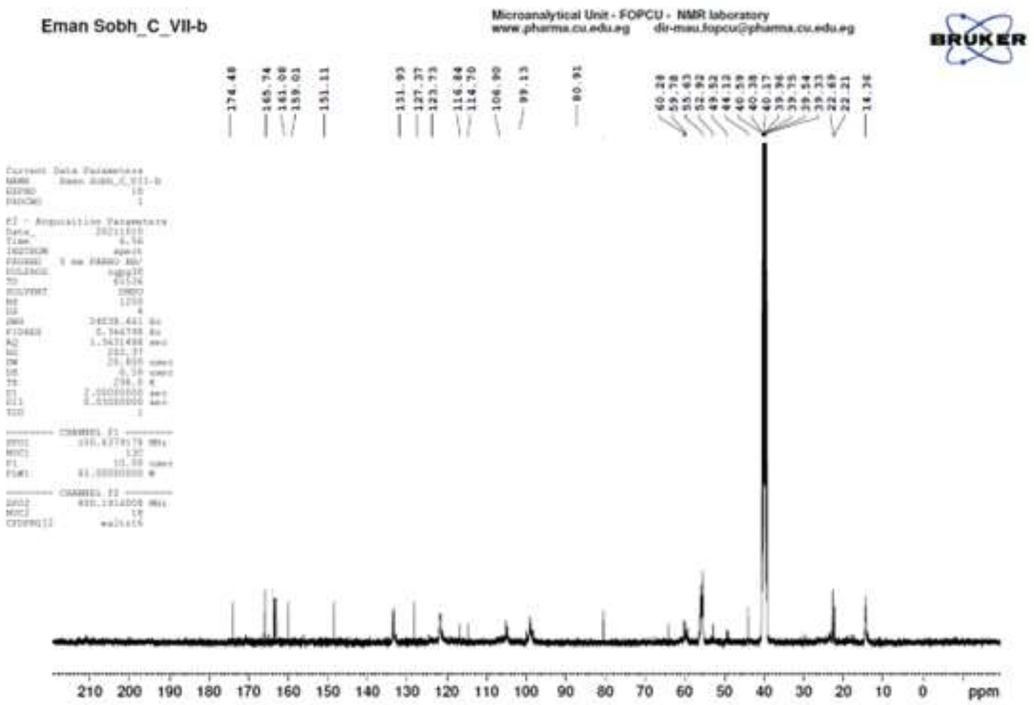

Figure S32. ^{13}**CNMR of compound 6b**

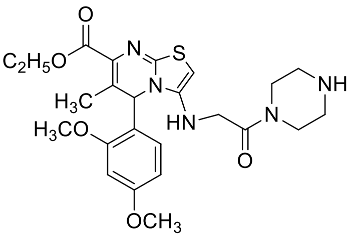

Information Classification: General

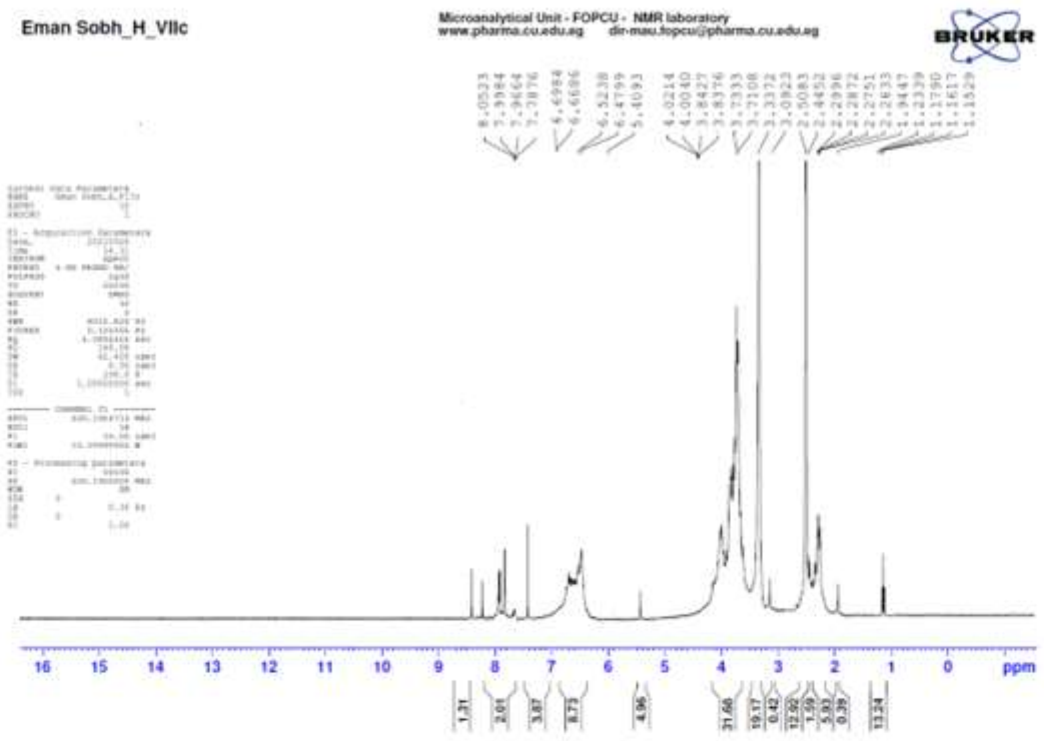

Figure S33. ^{1}HNMR of compound 6c

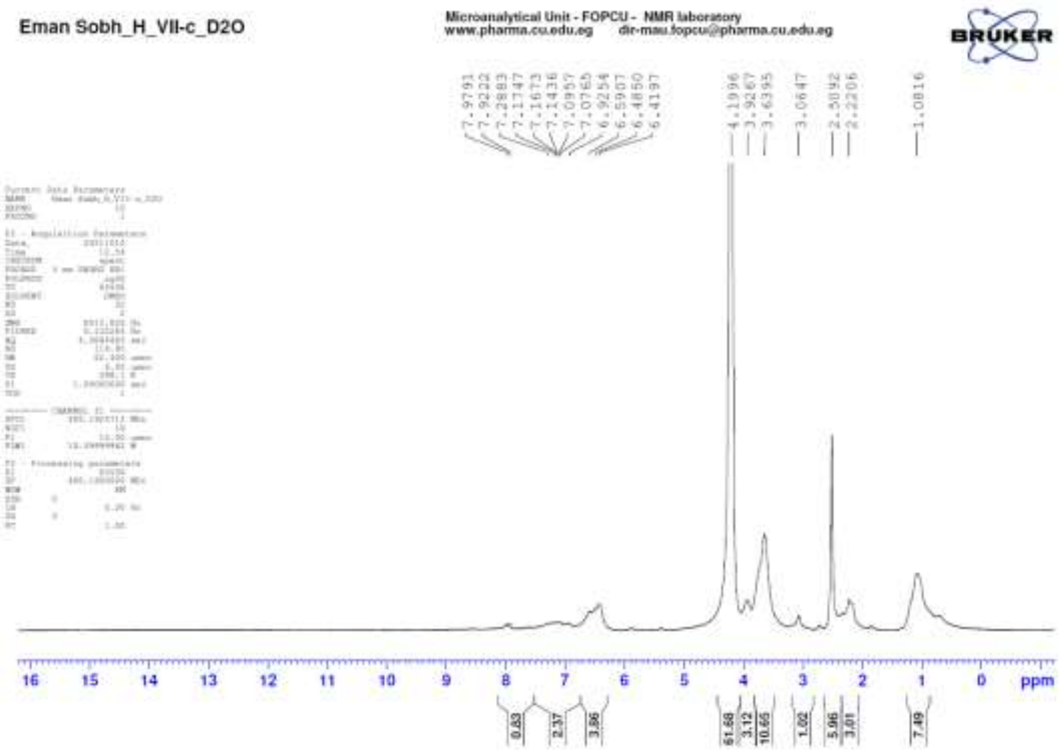

Figure S34. ^{1}HNMR (D2O) of compound 6c

Information Classification: General

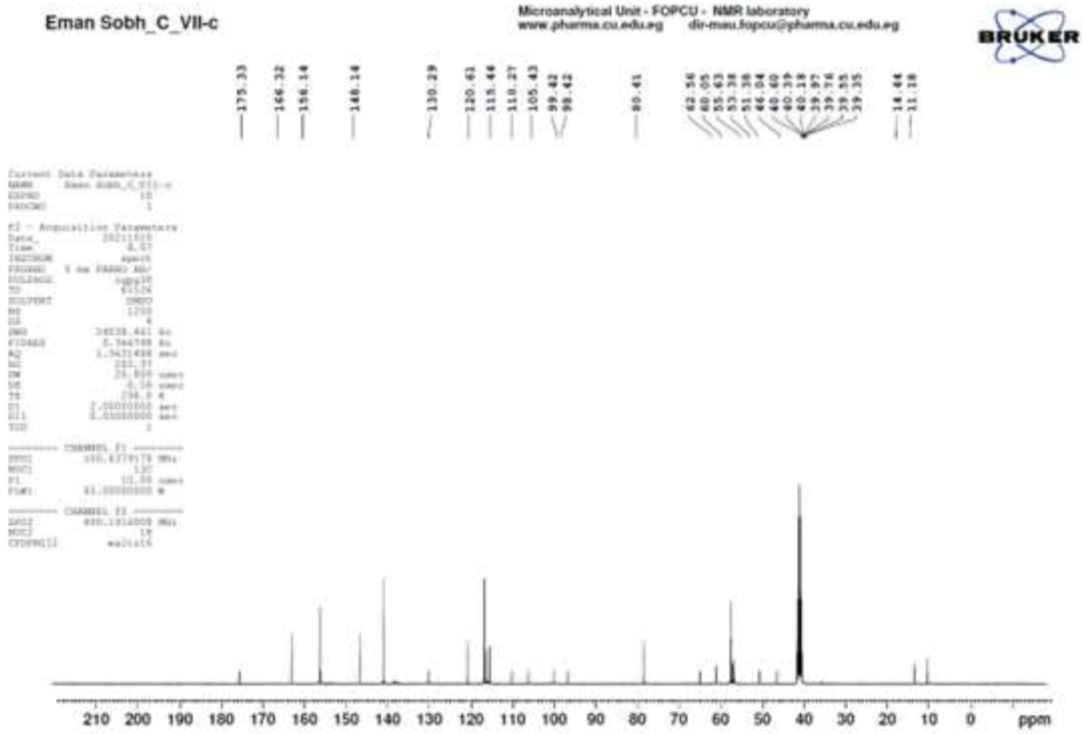

Figure S35. ^{13}**CNMR of compound 6c**

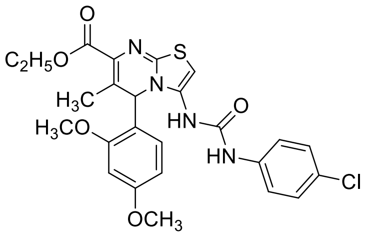

Information Classification: General

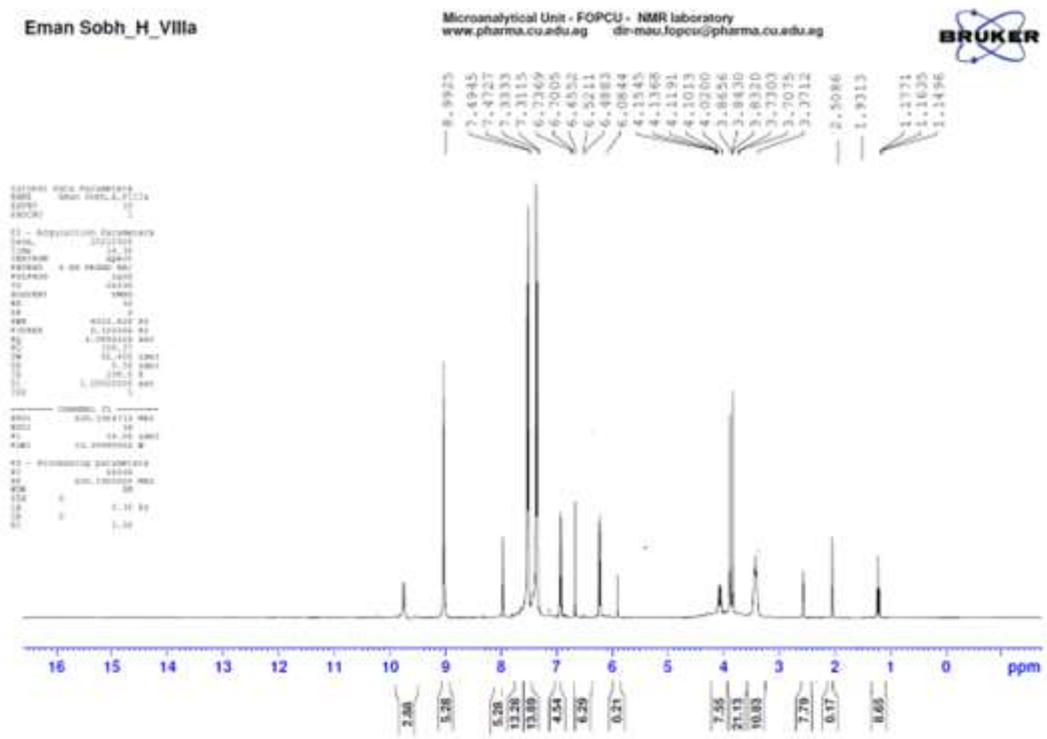

Figure S36. ^{1}HNMR of compound 7a

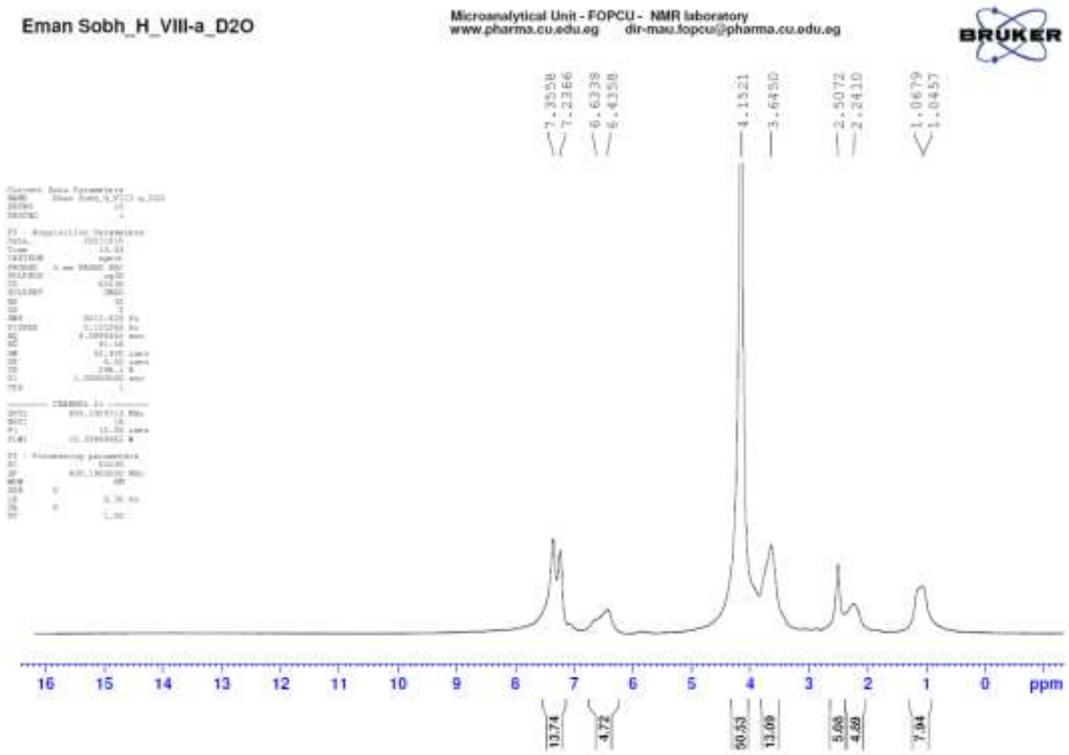

Figure S37. ^{1}HNMR (D_2O) of compound 7a

Information Classification: General

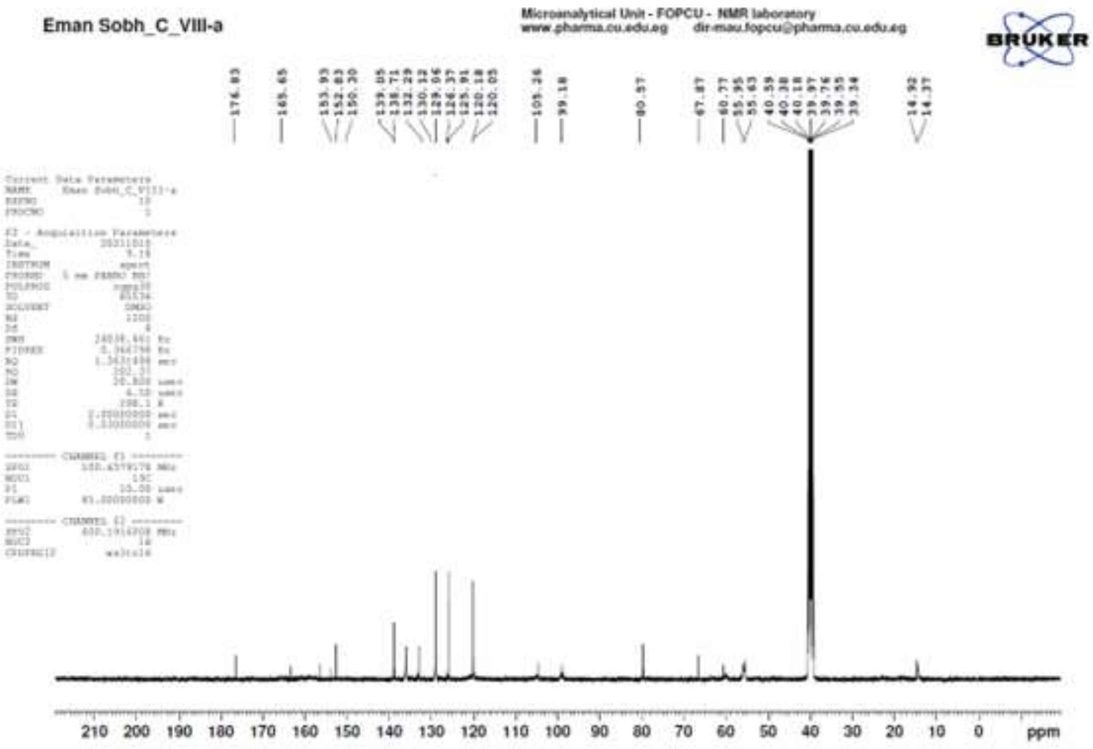

Figure S38. **^{13}CNMR of compound 7a**

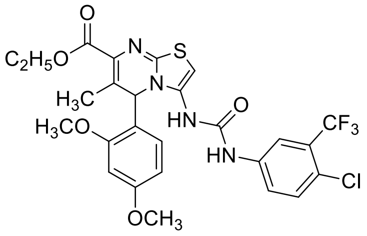

Information Classification: General

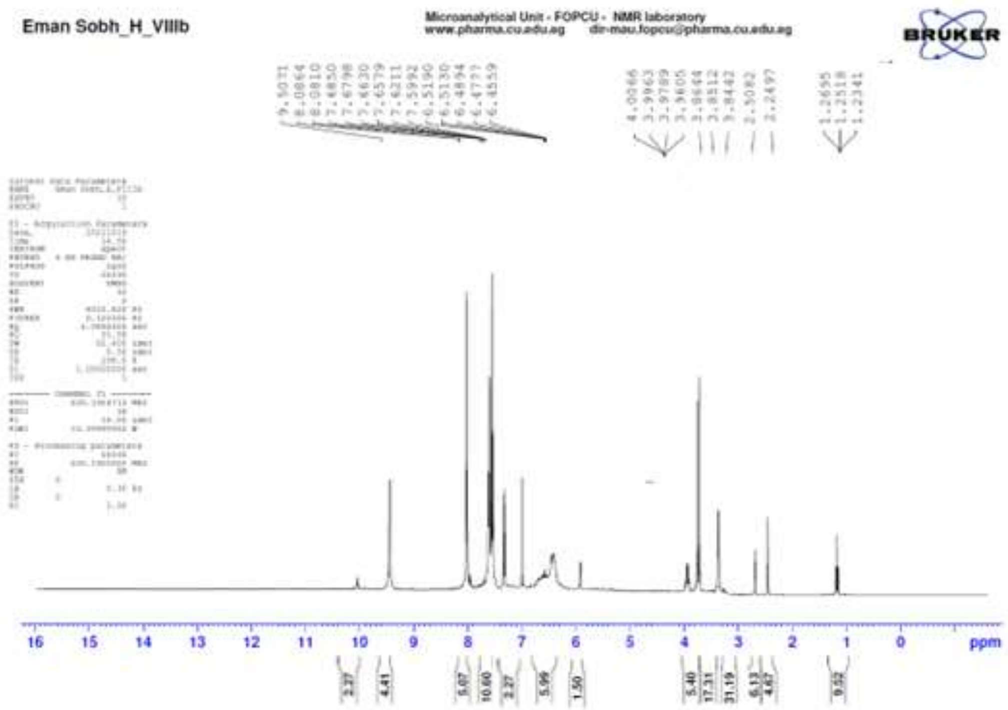

Figure S39. ^{1}HNMR of compound 7b

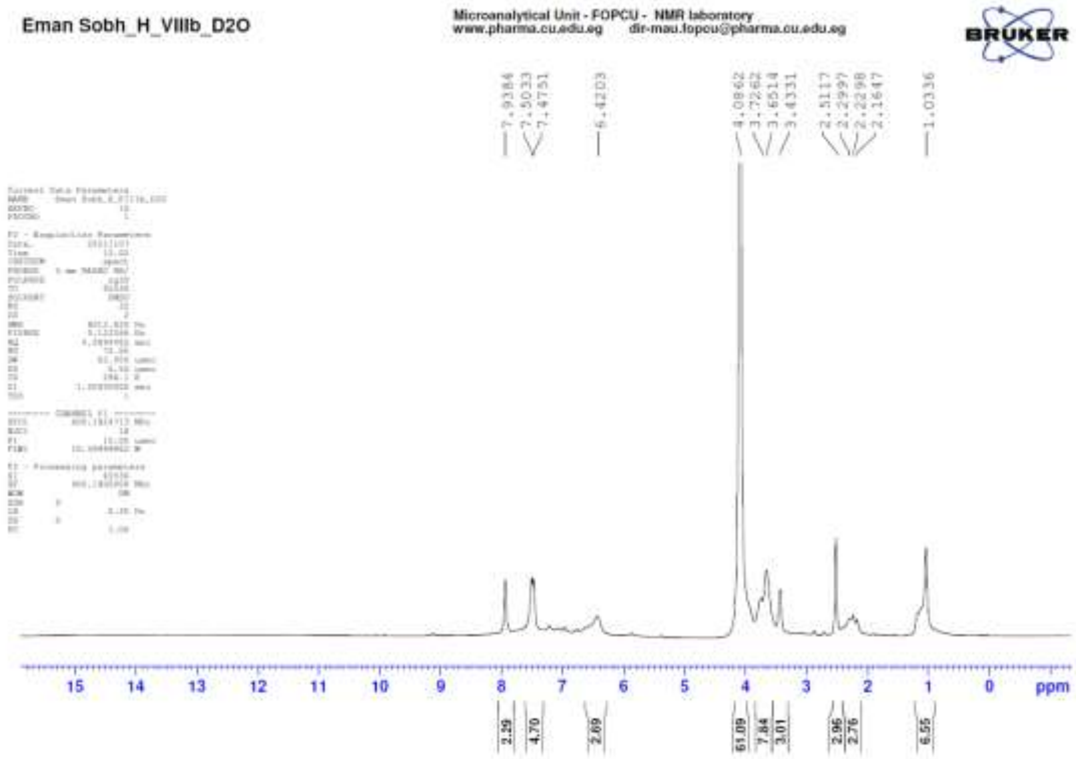

Figure S40. ^{1}HNMR (D2O) of compound 7b

Information Classification: General

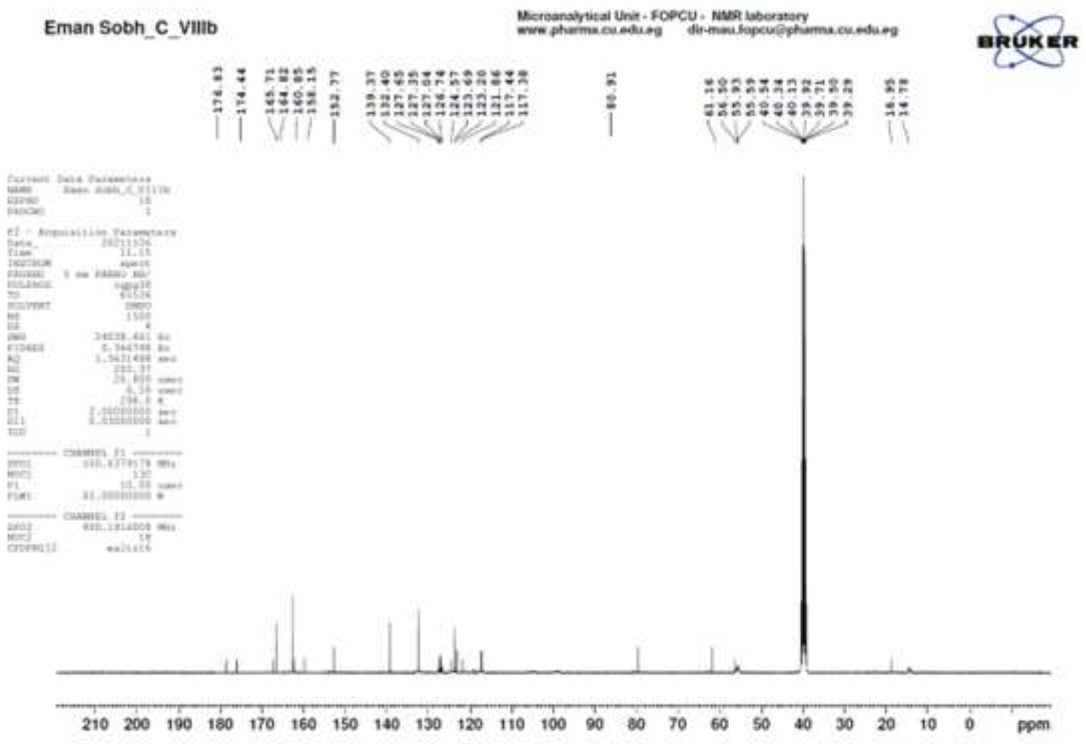

Figure S41. ^{13}**CNMR of compound 7b**

Information Classification: General

MTT assay calculations for the synthesized compounds (1, 2, 3a, 3b, 3c, 3d, 4a, 4b, 4c, 5, 6a, 6b, 6c, 7a and 7b, respectively) on MCF-7, A549 and A498 cell lines using doxorubicin reference.

researcher	assay	Date	cells		
Dr/Eman Sobh	MTT	16-Nov	**MCF7**	**A549**	**A498**

	**Blank**	**CC**	**Sample No. I/MCF7**					**Sample No. II/MCF7**				
	**1**	**2**	**3**	**4**	**5**	**6**	**7**	**8**	**9**	**10**	**11**	**12**
A	B	C	100ug	25ug	6.3ug	1.6ug	0.4ug	100ug	25ug	6.3ug	1.6ug	0.4ug
B	B	C	100ug	25ug	6.3ug	1.6ug	0.4ug	100ug	25ug	6.3ug	1.6ug	0.4ug
C	B	C	100ug	25ug	6.3ug	1.6ug	0.4ug	100ug	25ug	6.3ug	1.6ug	0.4ug

ROBONIK P2000 Eia reader

Wave length: 450 nm

Reference: 630 nm

	**1**	**2**	**3**	**4**	**5**	**6**	**7**	**8**	**9**	**10**	**11**	**12**
A	0.002	0.549	0.241	0.284	0.326	0.367	0.445	0.195	0.277	0.322	0.345	0.413
B	0.001	0.551	0.236	0.279	0.331	0.371	0.432	0.173	0.256	0.309	0.361	0.392
C	0.001	0.537	0.252	0.273	0.346	0.369	0.449	0.214	0.259	0.327	0.355	0.385
mean	0.0005	0.546	0.243	0.279	0.3343	0.369	0.442	0.194	0.264	0.3193	0.3537	0.3967
%		`	44.533	51.07	61.271	67.624	81.002	35.553	48.38	58.522	64.814	72.694

I/MCF7

log conc.	% viability
2	44.533
1.4	51.069
0.8	61.271
0.19	67.624
-0.41	81.002

IC50=

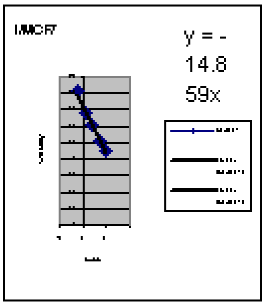

II/MCF7

log conc.	% viability
2	35.55
1.3979	48.38
0.7959	58.52
0.1931	64.81
-0.409	72.69

IC50=

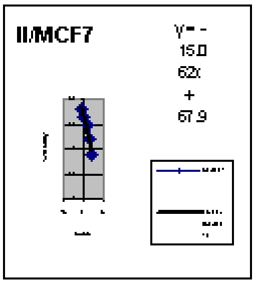

Information Classification: General

[redacted]

	Blank	CC	Sample No. IIIa/MCF7					Sample No. IIIb/MCF7				
	1	2	3	4	5	6	7	8	9	10	11	12
A	B	C	100ug	25ug	6.3ug	1.6ug	0.4ug	100ug	25ug	6.3ug	1.6ug	0.4ug
B	B	C	100ug	25ug	6.3ug	1.6ug	0.4ug	100ug	25ug	6.3ug	1.6ug	0.4ug
C	B	C	100ug	25ug	6.3ug	1.6ug	0.4ug	100ug	25ug	6.3ug	1.6ug	0.4ug

ROBONIK P2000 Eia reader

Wave length: 450 nm

Reference: 630 nm

	1	2	3	4	5	6	7	8	9	10	11	12
A	0.001	0.544	0.254	0.305	0.345	0.392	0.443	0.176	0.236	0.296	0.346	0.419
B	0.001	0.569	0.246	0.313	0.349	0.385	0.452	0.169	0.241	0.294	0.344	0.427
C	0.001	0.582	0.238	0.306	0.351	0.384	0.439	0.181	0.229	0.286	0.347	0.423
mean	0.001	0.565	0.246	0.308	0.3483	0.387	0.4447	0.1753	0.235	0.292	0.3457	0.423
% viability			43.54	54.51	61.652	68.496	78.702	31.032	41.65	51.681	61.18	74.867

IIIa/MCF7

log conc.	% viability
2	43.54
1.4	54.513
0.8	61.652
0.19	68.496
-0.41	78.702

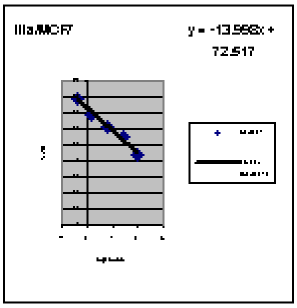

IC50=

IIIb/MCF7

log conc.	% viability
2	31.03
1.3979	41.65
0.7959	51.68
0.1931	61.18
-0.409	74.87

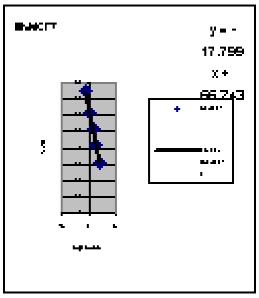

IC50=

[redacted]

	Blank	CC	Sample No. IIIc/MCF7					Sample No. IIId/MCF7				
	1	2	3	4	5	6	7	8	9	10	11	12

Information Classification: General

A	B	C	100ug	25ug	6.3ug	1.6ug	0.4ug	100ug	25ug	6.3ug	1.6ug	0.4ug
B	B	C	100ug	25ug	6.3ug	1.6ug	0.4ug	100ug	25ug	6.3ug	1.6ug	0.4ug
C	B	C	100ug	25ug	6.3ug	1.6ug	0.4ug	100ug	25ug	6.3ug	1.6ug	0.4ug

ROBONIK P2000 Eia reader

Wave length: 450 nm

Reference: 630 nm

	1	2	3	4	5	6	7	8	9	10	11	12
A	0.001	0.644	0.185	0.232	0.284	0.326	0.363	0.146	0.205	0.253	0.292	0.361
B	0.003	0.623	0.175	0.235	0.275	0.328	0.369	0.132	0.191	0.256	0.294	0.352
C	0.001	0.619	0.177	0.228	0.287	0.327	0.364	0.127	0.198	0.264	0.296	0.359
mean	0.0017	0.629	0.179	0.232	0.282	0.327	0.3653	0.135	0.198	0.2577	0.294	0.3573
% viability			28.473	36.85	44.857	52.015	58.112	21.474	31.5	40.986	46.766	56.84

IIIc/MCF7

2	28.473
1.4	36.85
0.8	44.857
0.19	52.015
-0.41	58.112

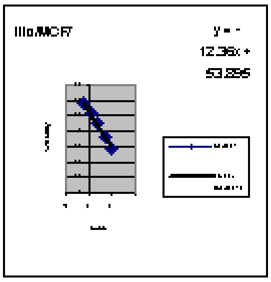

IC50=

IIId/MCF7

2	21.47
1.3979	31.5
0.7959	40.99
0.1931	46.77
-0.409	56.84

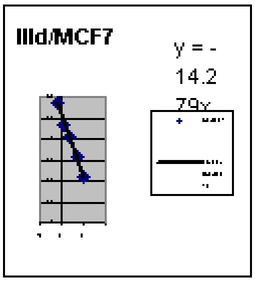

IC50=

	Blank	CC	Sample No. Vb/MCF7					Sample No. Vc/MCF7				
	1	2	3	4	5	6	7	8	9	10	11	12
A	B	C	100ug	25ug	6.3ug	1.6ug	0.4ug	100ug	25ug	6.3ug	1.6ug	0.4ug
B	B	C	100ug	25ug	6.3ug	1.6ug	0.4ug	100ug	25ug	6.3ug	1.6ug	0.4ug
C	B	C	100ug	25ug	6.3ug	1.6ug	0.4ug	100ug	25ug	6.3ug	1.6ug	0.4ug

ROBONIK P2000 Eia reader

Wave length: 450 nm

Information Classification: General

Reference: 630 nm

	1	2	3	4	5	6	7	8	9	10	11	12
A	0.001	0.494	0.212	0.264	0.294	0.342	0.395	0.185	0.224	0.275	0.324	0.367
B	0.003	0.521	0.202	0.253	0.313	0.347	0.392	0.169	0.235	0.271	0.328	0.372
C	0.001	0.483	0.208	0.259	0.316	0.349	0.386	0.188	0.227	0.267	0.331	0.374
mean	0.0017	0.499	0.2073	0.259	0.3077	0.346	0.391	0.1807	0.229	0.271	0.3277	0.371
% viability			41.522	51.8	61.615	69.292	78.304	36.182	45.79	54.272	65.621	74.299

Vb/MCF7

2	41.522
1.4	51.802
0.8	61.615
0.19	69.292
-0.41	78.304

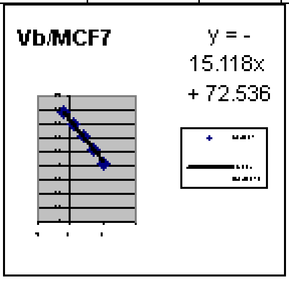

IC50=

Vc/MCF7

2	36.18
1.3979	45.79
0.7959	54.27
0.1931	65.62
-0.409	74.3

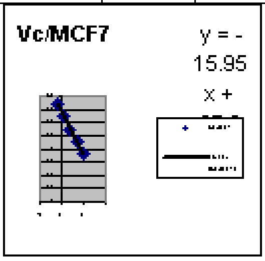

IC50=

	Blank	CC	Sample No. VI/MCF7					Sample No. VIIa/MCF7				
	1	2	3	4	5	6	7	8	9	10	11	12
A	B	C	100ug	25ug	6.3ug	1.6ug	0.4ug	100ug	25ug	6.3ug	1.6ug	0.4ug
B	B	C	100ug	25ug	6.3ug	1.6ug	0.4ug	100ug	25ug	6.3ug	1.6ug	0.4ug
C	B	C	100ug	25ug	6.3ug	1.6ug	0.4ug	100ug	25ug	6.3ug	1.6ug	0.4ug

ROBONIK P2000 Eia reader

Wave length: 450 nm

Reference: 630 nm

	1	2	3	4	5	6	7	8	9	10	11	12
A	0.001	0.545	0.238	0.294	0.337	0.384	0.411	0.172	0.234	0.286	0.331	0.382
B	0.001	0.528	0.247	0.292	0.345	0.379	0.428	0.169	0.221	0.274	0.337	0.379

Information Classification: General

C	0.001	0.551	0.242	0.285	0.339	0.386	0.434	0.182	0.222	0.272	0.341	0.381
mean	0.001	0.541	0.2423	0.29	0.3403	0.383	0.4243	0.1743	0.226	0.2773	0.3363	0.3807
% viability			44.766	53.63	62.869	70.751	78.387	32.204	41.69	51.232	62.131	70.32

VI/MCF7

2	44.766
1.4	53.633
0.8	62.869
0.19	70.751
-0.41	78.387

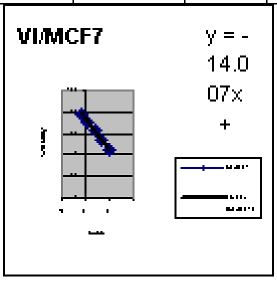

VIIa/MCF7

2	32.2
1.3979	41.69
0.7959	51.23
0.1931	62.13
-0.409	70.32

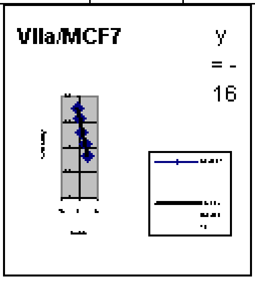

IC50=

IC50=

[redacted]

	Blank	CC	Sample No.	VIIb/MCF7				Sample No.	VIIc/MCF7			
	1	2	3	4	5	6	7	8	9	10	11	12
A	B	C	100ug	25ug	6.3ug	1.6ug	0.4ug	100ug	25ug	6.3ug	1.6ug	0.4ug
B	B	C	100ug	25ug	6.3ug	1.6ug	0.4ug	100ug	25ug	6.3ug	1.6ug	0.4ug
C	B	C	100ug	25ug	6.3ug	1.6ug	0.4ug	100ug	25ug	6.3ug	1.6ug	0.4ug

ROBONIK P2000 Eia reader

Wave length: 450 nm

Reference: 630 nm

	1	2	3	4	5	6	7	8	9	10	11	12
A	0.001	0.585	0.127	0.191	0.236	0.313	0.354	0.253	0.313	0.361	0.417	0.462
B	0.002	0.602	0.139	0.183	0.241	0.308	0.358	0.256	0.294	0.352	0.424	0.448
C	0.001	0.579	0.131	0.177	0.229	0.316	0.347	0.264	0.296	0.359	0.396	0.451
mean	0.0013	0.589	0.1323	0.184	0.2353	0.3123	0.353	0.2577	0.301	0.3573	0.4123	0.4537
% viability			22.48	31.2	39.977	53.058	59.966	43.771	51.13	60.702	70.045	77.067

VIIb/MCF7

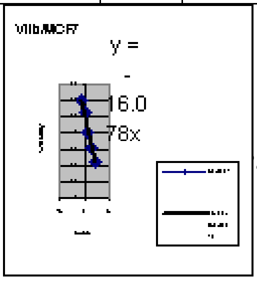

VIIc/MCF7

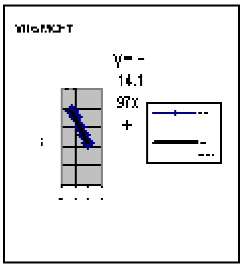

Information Classification: General

2	22.48
1.4	31.2
0.8	39.977
0.19	53.058
-0.41	59.966

2	43.77
1.3979	51.13
0.7959	60.7
0.1931	70.05
-0.409	77.07

IC50= [redacted]

IC50= [redacted]

	**Blank**	**CC**	**Sample No. VIIIa/MCF7**					**Sample No. VIIIb/MCF7**				
	**1**	**2**	**3**	**4**	**5**	**6**	**7**	**8**	**9**	**10**	**11**	**12**
A	B	C	100ug	25ug	6.3ug	1.6ug	0.4ug	100ug	25ug	6.3ug	1.6ug	0.4ug
B	B	C	100ug	25ug	6.3ug	1.6ug	0.4ug	100ug	25ug	6.3ug	1.6ug	0.4ug
C	B	C	100ug	25ug	6.3ug	1.6ug	0.4ug	100ug	25ug	6.3ug	1.6ug	0.4ug

ROBONIK P2000 Eia reader

Wave length: 450 nm

Reference: 630 nm

	**1**	**2**	**3**	**4**	**5**	**6**	**7**	**8**	**9**	**10**	**11**	**12**
A	0.003	0.564	0.242	0.286	0.324	0.366	0.409	0.181	0.245	0.298	0.343	0.379
B	0.001	0.552	0.251	0.292	0.331	0.371	0.417	0.196	0.237	0.303	0.346	0.388
C	0.001	0.538	0.258	0.294	0.336	0.379	0.412	0.185	0.242	0.306	0.352	0.394
mean	0.0017	0.551	0.2503	0.291	0.3303	0.372	0.4127	0.1873	0.241	0.3023	0.347	0.387
% viability			45.405	52.72	59.915	67.473	74.849	33.978	43.77	54.837	62.938	70.193

VIIIa/MCF7

2	45.405
1.4	52.721
0.8	59.915
0.19	67.473

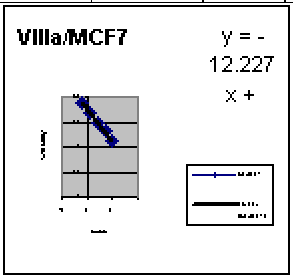

VIIIb/MCF7

2	33.98
1.3979	43.77
0.7959	54.84
0.1931	62.94

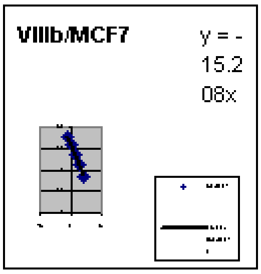

Information Classification: General

-0.41	74.849

-0.409	70.19

IC50=

IC50=

	Blank	CC	Sample No.		STA/MCF7			Sample No.				
	**1**	**2**	**3**	**4**	**5**	**6**	**7**	**8**	**9**	**10**	**11**	**12**
A	B	C	100ug	25ug	6.3ug	1.6ug	0.4ug					
B	B	C	100ug	25ug	6.3ug	1.6ug	0.4ug					
C	B	C	100ug	25ug	6.3ug	1.6ug	0.4ug					

ROBONIK P2000 Eia reader

Wave length: 450 nm

Reference: 630 nm

	**1**	**2**	**3**	**4**	**5**	**6**	**7**	**8**	**9**	**10**	**11**	**12**
A	0.001	0.515	0.185	0.232	0.284	0.326	0.363					
B	0.002	0.507	0.175	0.235	0.275	0.328	0.369					
C	0.001	0.511	0.177	0.228	0.287	0.327	0.364					
mean	0.0013	0.511	0.179	0.232	0.282	0.327	0.3653	0	0	0	0	0
% viability			35.029	45.34	55.186	63.992	71.494	0	0	0	0	0

STA/MCF7

2	35.029
1.4	45.336
0.8	55.186
0.19	63.992
-0.41	71.494

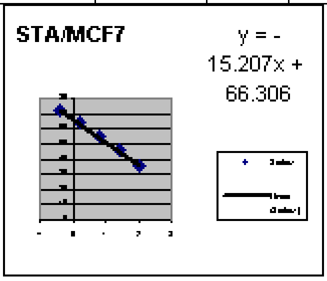

2	0
1.3979	0
0.7959	0
0.1931	0
-0.409	0

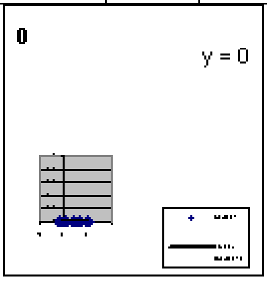

IC50=

IC50=

	Blank	CC	Sample No. Va/MCF7									
	1	2	3	4	5	6	7	8	9	10	11	12
	B	C	100ug	25ug	6.3ug	1.6ug	0.4ug	100ug	25ug	6.3ug	1.6ug	0.4ug
	B	C	100ug	25ug	6.3ug	1.6ug	0.4ug	100ug	25ug	6.3ug	1.6ug	0.4ug
	B	C	100ug	25ug	6.3ug	1.6ug	0.4ug	100ug	25ug	6.3ug	1.6ug	0.4ug

ROBONIK P2000 Ela reader

Wave length: 450 nm

Reference: 630 nm

	1	2	3	4	5	6	7

	0.001	0.526	0.085	0.245	0.298	0.343	0.379	0.437
	0.001	0.541	0.102	0.237	0.303	0.346	0.388	0.44
	0.001	0.529	0.076	0.242	0.306	0.352	0.394	0.451
	0.001	0.532	0.0877	0.2413	0.302	0.347	0.387	0.4427
y			16.479	45.363	56.83	65.226	72.744	83.208

Va/MCF7

log conc.	% viability
2	45.36
1.3979	56.83
0.7959	65.23
0.1931	72.74
-0.409	83.21

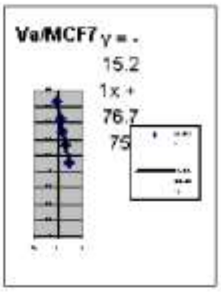

IC50=

[redacted] [redacted] [redacted]

Information Classification: General

IC$_{50}$ calculations of compounds (3b, 3c, 3d, 4c, 6b, 6b, 7a and 7b) against topoisomerase II enzymes together with doxorubicin and etoposide references.

TOPO II

[redacted]

code	IC50	conc	log	%inh
IIIb		100	2	80
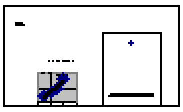		10	1	48
		1	0	30
		0.1	-1	19
EC				0

[redacted] [redacted]

code	IC50	conc	log	%inh
IIIc		100	2	89
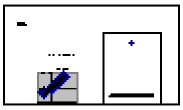		10	1	63
		1	0	45
		0.1	-1	31
EC				0

[redacted] [redacted]

code	IC50	conc	log	%inh
IIId		100	2	85
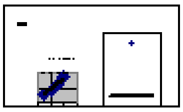		10	1	59
		1	0	41
		0.1	-1	28
EC				0

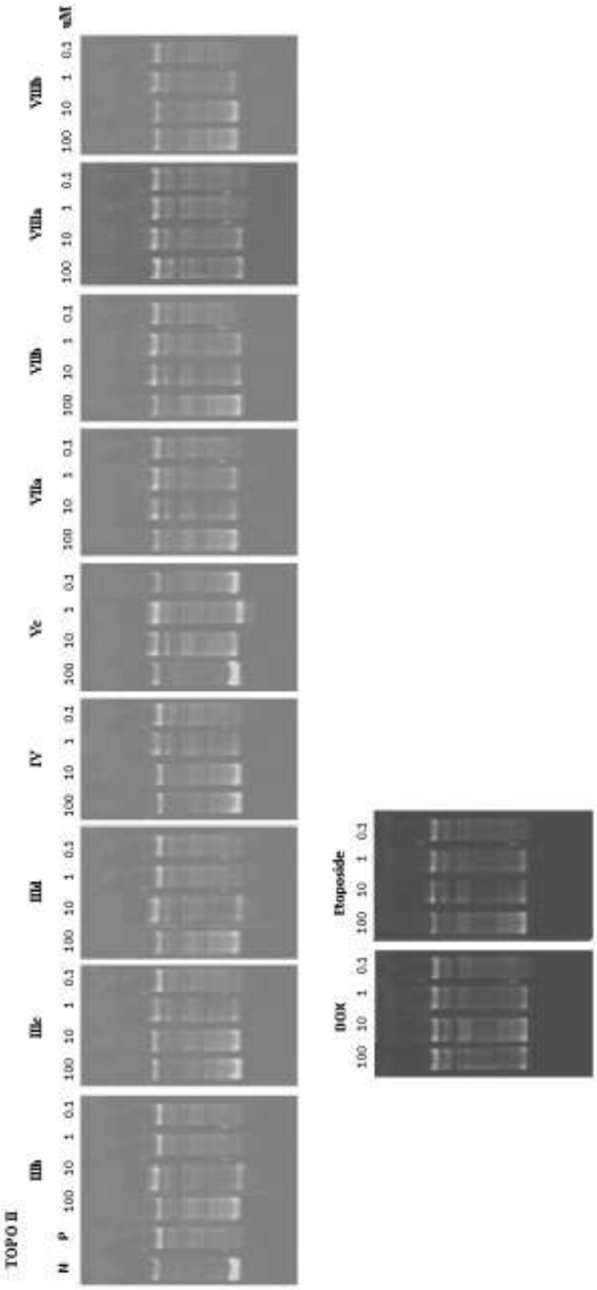

Information Classification: General

	[redacted]		[redacted]	
code	IC50	conc	log	%inh
Vc		100	2	92
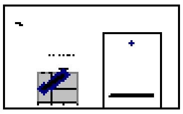		10	1	79
		1	0	60
		0.1	-1	44
EC				0

	[redacted]		[redacted]	
code	IC50	conc	log	%inh
VIIa		100	2	83
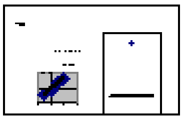		10	1	67
		1	0	42
		0.1	-1	26
EC				0

	[redacted]		[redacted]	
code	IC50	conc	log	%inh
VIIb		100	2	88
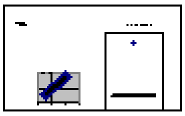		10	1	67
		1	0	44
		0.1	-1	28
EC				0

	[redacted]		[redacted]	
code	IC50	conc	log	%inh
VIIIa		100	2	84
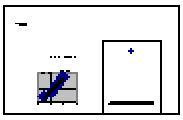		10	1	57

Information Classification: General

code	IC50	conc	log	%inh
		1	0	34
		0.1	-1	20
EC				0

[redacted]

code	IC50	conc	log	%inh
VIIIb		100	2	88

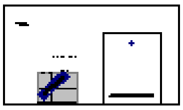

code	IC50	conc	log	%inh
		10	1	71
		1	0	52
		0.1	-1	28
EC				0

[redacted]

code	IC50	conc	log	%inh
Etoposide		100	2	92

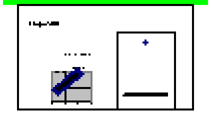

code	IC50	conc	log	%inh
		10	1	80
		1	0	60
		0.1	-1	39
EC				0

[redacted]

code	IC50	conc	log	%inh
Dox		100	2	90

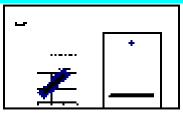

code	IC50	conc	log	%inh
		10	1	77
		1	0	51
		0.1	-1	30
EC				0

[redacted]

**IIIb**	$y = 20.282x + 34.018$
**IIIc**	$y = 19.353x + 47.242$
**IIId**	$y = 18.983x + 43.466$

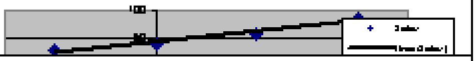

**Vc**	$y = 16.319x + 60.43$
**VIIa**	$y = 19.661x + 44.592$
**VIIb**	$y = 20.309x + 46.389$
**VIIIa**	$y = 21.635x + 38.07$
**VIIIb**	$y = 20.12x + 49.611$
**Etoposide**	$y = 18.145x + 58.917$
**Dox**	$y = 20.713x + 51.727$

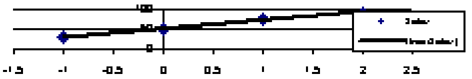

Information Classification: General

MTT assay on WI38 normal cell line for compound 4c using doxorubicin reference

researcher	assay	Date	cells
Dr.Eman Sobh	MTT	02/03/2022	WI38

	Blank	CC	Sample No.	Vc/WI38 8			
	1	2	3	4	5	6	7
A	B	C	100uM	25uM	6.3uM	1.6uM	0.4uM
B	B	C	100uM	25uM	6.3uM	1.6uM	0.4uM
C	B	C	100uM	25uM	6.3uM	1.6uM	0.4uM

ROBONIK P2000 Ela reader

Wave length: 450 nm

Reference: 630 nm

	1	2	3	4	5	6	7
A	0.001	0.528	0.184	0.239	0.294	0.359	0.411
B	0.001	0.541	0.193	0.251	0.313	0.361	0.409
C	0.001	0.526	0.181	0.246	0.318	0.372	0.425
mean	4E-04	0.532	0.186	0.245	0.3083	0.364	0.415
%			34.984	46.14	57.994	68.46	78.056

Vc/WI38

log conc.	% viability
2	34.98
1.3979	46.14
0.7959	57.99
0.1931	68.46
-0.4089	78.06

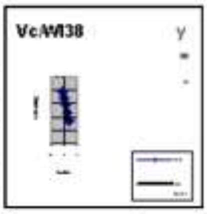

IC50= [redacted]

	Blank	CC	Sample No.	Dox/WI38			
	1	2	3	4	5	6	7
A	B	C	100uM	25uM	6.3uM	1.6uM	0.4uM
B	B	C	100uM	25uM	6.3uM	1.6uM	0.4uM
C	B	C	100uM	25uM	6.3uM	1.6uM	0.4uM

ROBONIK P2000 Ela reader

Wave length: 450 nm

Reference: 630 nm

	1	2	3	4	5	6	7
A	0.001	0.546	0.182	0.234	0.292	0.344	0.421
B	0.001	0.551	0.179	0.238	0.285	0.339	0.394
C	0.001	0.539	0.186	0.235	0.284	0.361	0.433
mean	0.001	0.545	0.182	0.2357	0.287	0.348	0.416
% viability			33.44	43.215	52.628	63.81	76.28

Dox/WI38

log conc.	% viability
2	33.44
1.398	43.22
0.796	52.63
0.193	63.81
-0.409	76.28

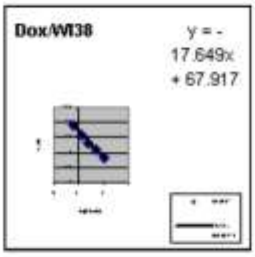

IC50= [redacted]

Information Classification: General

Information Classification: General
